# Chemoprotective antimalarials identified through quantitative high-throughput screening of *Plasmodium* blood and liver stage parasites

**DOI:** 10.1038/s41598-021-81486-z

**Published:** 2021-01-22

**Authors:** Dorjbal Dorjsuren, Richard T. Eastman, Kathryn J. Wicht, Daniel Jansen, Daniel C. Talley, Benjamin A. Sigmon, Alexey V. Zakharov, Norma Roncal, Andrew T. Girvin, Yevgeniya Antonova-Koch, Paul M. Will, Pranav Shah, Hongmao Sun, Carleen Klumpp-Thomas, Sachel Mok, Tomas Yeo, Stephan Meister, Juan Jose Marugan, Leila S. Ross, Xin Xu, David J. Maloney, Ajit Jadhav, Bryan T. Mott, Richard J. Sciotti, Elizabeth A. Winzeler, Norman C. Waters, Robert F. Campbell, Wenwei Huang, Anton Simeonov, David A. Fidock

**Affiliations:** 1 Division of Preclinical Innovation, National Center for Advancing Translational Sciences, National Institutes of Health, Rockville, MD 20850 USA; 2 Department of Microbiology and Immunology, Columbia University Irving Medical Center, New York, NY 10032 USA; 3 Experimental Therapeutics Branch, Walter Reed Army Institute of Research, Silver Spring, MD 20910 USA; 4 Palantir Technologies, Washington, DC 20007 USA; 5 Department of Pediatrics, Pharmacology and Drug Discovery, School of Medicine, University of California San Diego, La Jolla, CA 92093 USA; 6 Division of Infectious Diseases, Department of Medicine, Columbia University Irving Medical Center, New York, NY 10032 USA; 7 Present Address: Drug Discovery and Development Centre (H3D), University of Cape Town, Rondebosch, 7701 South Africa; 8 Present Address: Calibr, The Scripps Research Institute, La Jolla, CA 92037 USA; 9 Present Address: Beckman Coulter Inc., Carlsbad, CA 92010 USA; 10 Present Address: Veralox Therapeutics Inc., Frederick, MD 21704 USA

**Keywords:** High-throughput screening, Phenotypic screening, Parasitic infection

## Abstract

The spread of *Plasmodium falciparum* parasites resistant to most first-line antimalarials creates an imperative to enrich the drug discovery pipeline, preferably with curative compounds that can also act prophylactically. We report a phenotypic quantitative high-throughput screen (qHTS), based on concentration–response curves, which was designed to identify compounds active against *Plasmodium* liver and asexual blood stage parasites. Our qHTS screened over 450,000 compounds, tested across a range of 5 to 11 concentrations, for activity against *Plasmodium falciparum* asexual blood stages. Active compounds were then filtered for unique structures and drug-like properties and subsequently screened in a *P. berghei* liver stage assay to identify novel dual-active antiplasmodial chemotypes. Hits from thiadiazine and pyrimidine azepine chemotypes were subsequently prioritized for resistance selection studies, yielding distinct mutations in *P. falciparum* cytochrome b, a validated antimalarial drug target. The thiadiazine chemotype was subjected to an initial medicinal chemistry campaign, yielding a metabolically stable analog with sub-micromolar potency. Our qHTS methodology and resulting dataset provides a large-scale resource to investigate *Plasmodium* liver and asexual blood stage parasite biology and inform further research to develop novel chemotypes as causal prophylactic antimalarials.

## Introduction

Malaria, a parasitic disease spread to humans through the bite of a *Plasmodium*-infected *Anopheles* mosquito, continues to have a substantial global health impact, with an estimated 229 million cases and 409,000 deaths in 2019^1^. During an infected mosquito blood meal, *Plasmodium* parasites in the form of sporozoites travel to the host’s hepatocytes wherein they propagate as liver stages, producing thousands of merozoites. These merozoites rupture from the hepatocyte and transform into asexual blood stage (ABS) parasites that undergo successive rounds of erythrocyte invasion, replication and rupture. The cyclic ABS replication results in the characteristic symptoms of malaria, typified by bouts of fever and chills. The risk of progressing to fatal disease is due mostly to *P. falciparum*^2^. While less lethal, *P. vivax* and *P. ovale* can form quiescent liver stage forms called hypnozoites that remain dormant and later reactivate to initiate an ABS infection (relapse)^3^.

Antimalarial drugs possess varying efficacy against different stages of *Plasmodium* infection in patients. Therapeutic agents only treat the symptomatic ABS, whereas causal prophylactic agents are active against both liver and ABS stages, thereby reducing the emergence of new parasites from the liver. Quinine (QN), active against ABS parasites, was used extensively until supply shortages and the emergence of drug-resistant parasites spurred the development of novel agents, resulting in chloroquine (CQ), mefloquine (MFQ), sulfadoxine-pyrimethamine (SP), and primaquine (PMQ)^4^. These initially proved to be highly effective, with CQ, a 4-aminoquinoline, becoming the mainstay antimalarial for decades. The emergence and spread of CQ-resistant parasites across South America, Southeast Asia, India, and Africa in the 1960s to ‘80 s, however, ablated its utility^5^.

More recently, artemisinin-based combination therapies (ACTs), which combine a fast-acting derivative of the endoperoxide artemisinin (ART) with an antimalarial partner drug, have been used as first-line treatment for ABS infection across virtually all malaria-endemic regions. This approach has helped achieve a major reduction in the global disease burden, with annual malaria deaths reducing from approximately one million cases to 400,000 over the period 2000 to 2015^1^. Since then, however, malaria mortality and morbidity rates have reached a plateau. Clearly, additional strategies are required to further reduce the impact of malaria across the endemic, inter-tropical regions of the globe.

Developing additional causal prophylactic antimalarials would be an important advance in this area. Neither ARTs nor many of the ACT partner drugs are active against either the proliferative liver stage or hypnozoites in the case of *P. vivax* or *P. ovale*^6,7^. Until recently, the only approved treatments effective against the liver stage were atovaquone (ATQ)-proguanil, SP, and PMQ, with the latter also being effective against hypnozoites. In 2018, tafenoquine (TFQ), an 8-aminoquinoline compound structurally related to PMQ, was approved as both a chemoprophylactic and single-dose treatment for liver stage infection that targets both replicating and hypnozoite forms^8^. The TFQ single-dose anti-relapse treatment is an important improvement over the existing 14-day treatment regimen employed for PMQ. Both PMQ and TFQ, however, are contraindicated in patients with glucose-6-phosphate dehydrogenase (G6PD) deficiency, due to the risk of hemolytic anemia^9^. The high prevalence of G6PD deficiency in many malaria-endemic countries limits the widespread, safe usage of PMQ and TFQ^10^. This issue could be addressed through point-of-care G6PD diagnostics. While several tests are in development they still require further field evaluation and implementation^11^. Common CYP2D6 polymorphisms can also result in individuals poorly metabolizing these 8-aminoquinolines to their active form^12^.

Another limitation of the currently available chemoprophylactic antimalarials is the increasing prevalence of drug-resistant parasites^13^. For example, *P. falciparum* resistance to prophylactic drugs is ever more prevalent, and the protective effect of SP for intermittent preventive treatment (IPT) in infants is significantly reduced in regions with an elevated prevalence of resistance-conferring mutations in *P. falciparum dhfr* and *dhps*^14–16^. Similarly, trials assessing seasonal malaria chemoprotection (SMC) using SP with either amodiaquine or the ART derivative artesunate found a significant increase in the prevalence of parasites containing drug-resistant alleles in the treatment arm compared to placebo^17,18^. Additionally, some chemoprophylactic antimalarials including doxycycline, PMQ and TFQ are contraindicated while breast feeding or during pregnancy, owing to either adverse effects or because the G6PD status of the fetus cannot be ascertained^2,19^. These limitations emphasize the need for new therapeutic options, whose development has been hindered by the scarcity of novel chemotypes and established therapeutic targets effective against the liver stage^20,21^.

Inhibition of liver stage parasites would not only prevent symptomatic infection and limit transmission but should also deter the development of drug-resistant parasites. There is a significantly lower liver stage parasite burden (~ 10^3^ to 10^4^ parasites) compared to the ABS (10^8^ to 10^12^ parasites)^22,23^, which limits both the number of parasites exposed to the drug and the probability of spontaneous genetic resistance arising^24^. Previously, liver stage assays to identify active compounds were hindered by the technical difficulty of isolating sporozoites, the need for specialized imaging devices, and the lack of high-throughput formats. However, the generation of a rodent parasite strain, *P. berghei*, expressing a GFP-Luc reporter (Pb-Luc) has enabled the development of a sensitive, high-throughput, luciferase-based assay for screening chemical libraries to identify compounds with liver stage activity^25–27^.

Here, a chemoprophylactic drug discovery effort was conducted through dual phenotypic high-throughput ABS and liver stage screens, providing a robust foundation to identify liver-stage active lead chemotypes^26,28^. We combined the results obtained from our testing of 151,842 small molecules against *P. falciparum* ABS parasites with the results of our prior unpublished screen of the Molecular Libraries Small Molecule Repository (MLSMR) collection (PubChem AID 488774). This provided a quantitative high-throughput profile of 456,817 compounds, of which 994 ABS-active compounds were selected for profiling against *Plasmodium* liver stage parasites.

These screening efforts identified several novel chemotypes efficacious against both asexual blood and liver stage parasites, including subsets containing thiadiazine or pyrimidine azepine scaffolds. Further analysis via drug pressuring experiments with cultured ABS parasites identified a resistance mechanism involving the *P. falciparum* cytochrome *bc*_*1*_ complex, a validated malaria drug target and component of the mitochondrial electron transport chain^29^.

This study provides a multi-point high-throughput route for successfully identifying novel antimalarial chemotypes via triaging and clustering ABS hit compounds based on chemotype and activity, secondary screening with liver stage cultures and in vitro pharmacological profiling (testing for membrane permeability, aqueous solubility, and hepatic microsomal stability), and elucidation of mechanism of action via resistance selection experiments. Several additional medicinal chemistry campaigns can now stem from these screening efforts, emphasizing the downstream benefit of generating these screening data.

## Results

### Quantitative high-throughput screening of over 450,000 compounds tested against *P. falciparum* asexual blood stage parasites and selection of 994 validated antiplasmodial compounds

**Figure 1:**
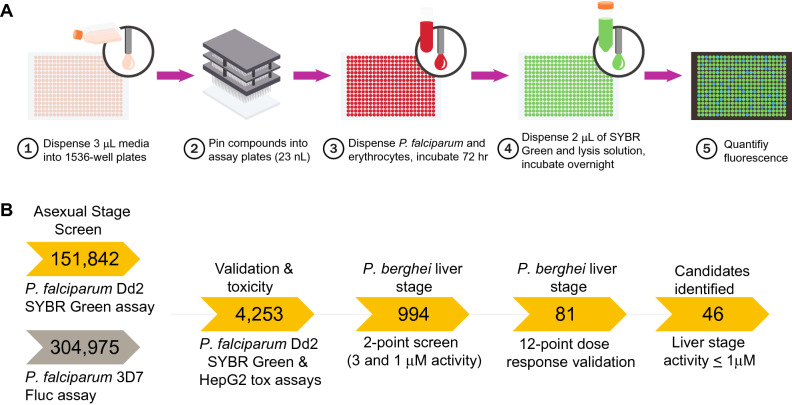
Implementation of a quantitative high-throughput (qHTS) screen against *P. falciparum* asexual blood stage parasites. (**A**) Overview of qHTS primary and secondary screens of compound libraries assayed against *P. falciparum* ABS parasites cultured in human RBCs. In our second major screen, growth inhibition was monitored via quantification of SYBR Green fluorescence, which detects parasite DNA in human DNA-deficient RBCs. Alternatively, inhibition of parasite proliferation was quantified by measuring luciferase activity (not shown). (**B**) Screening pipeline for identifying candidate causal chemoprophylactic antimalarials. Two large qHTS campaigns used either SYBR Green or luciferase to test for inhibition of parasite growth, assaying a combined total of 456,817 compounds. Following qHTS, manual triage selected 4253 synthetically tractable compounds for validation of antiplasmodial activity and mammalian toxicity. Compounds were then evaluated in vitro against *P. berghei* liver stage parasites at 3 μM and 1 μM concentrations. Selected compounds were further evaluated in a 12-point dose–response against *P. berghei* liver stage parasites, yielding 46 with submicromolar AC_50_ values.

To identify chemical compounds active against *Plasmodium* asexual blood and liver stage parasites, we started by miniaturizing a SYBR Green-I based assay^30^ into a 1536-well format. Using this primary assay, we performed multi-point screening of a diverse chemical collection of 151,842 compounds against *P. falciparum* Dd2 asexual blood stage parasites cultured in vitro. The compounds tested were arrayed as six-point titrations, at final concentrations ranging from 28 μM to 2.9 nM (Fig. 1A; PubChem AID 1347417). The assay performed well during the entire course of the whole-cell screen, with an average Z’-score of 0.63 (500 plates screened, Z’ range 0.1–0.9). In addition, the intra-plate control titration of dihydroartemisinin yielded a near-constant concentration–response curve with an average AC_50_ of 2.0 nM and a minimum significant ratio of 1.9 (the best possible ratio is 1.0; ratios of less than 4 are generally indicative of an assay with high test-to-test reproducibility of dose responses)^31^. The AC_50_ defines the relative mean concentration at which parasite growth was inhibited by 50%. We then selected a total of 2496 compounds for further validation, based on their exhibiting > 80% efficacy (defined as the normalized difference between neutral and the maximum compound inhibition response), robust concentration–response curves (exhibiting upper and lower asymptotes and curve classes of − 1.1 or − 1.2 as defined in^32^), and AC_50_ values < 2 μM.

In addition, 1757 compounds were also manually curated from our earlier unpublished screening at NCATS of 304,975 unique compounds present in the Molecular Libraries Small Molecule Repository collection. That screen employed a *P. falciparum* 3D7 recombinant Firefly luciferase (Fluc) reporter line (Fig. 1B) and detailed results are available in PubChem AID 488774. This resulted in a total set of 456,817 compounds that were tested using our qHTS with cultured ABS parasites.

Our combined set of 4253 compounds with AC_50_ values < 2 μM from the primary screens were re-screened using the SYBR Green-I proliferation assay, with 11-point intra-plate concentrations to confirm growth inhibition (PubChem AID 1347416; screening of 37 plates with a Z’-score mean of 0.5, range 0.3–0.7). Of these, 2223 (52.7%) were reconfirmed, with 968 (23.0%) yielding an inconclusive result and 1025 (24.3%) being inactive (Supplementary Table 1). Cellular toxicity against HepG2 was also assessed for all 4253 compounds using a CellTiter Glo assay (screening of 37 plates with a Z’-score mean of 0.82, range 0.7–0.9). 255 compounds exhibited some level of growth cellular toxicity against HepG2 cells, with AC_50_ values < 43 µM, and were subsequently excluded (Supplementary Table 2). Based on potency and chemical novelty, we ultimately selected 994 validated, non-toxic ABS hits for subsequent assessment against liver stage parasites.

A counter screen of these 4253 compounds was also performed against the Fluc enzyme, as both liver stage methods utilized a transgenic *P. berghei* Fluc parasite. From these compounds, 310 had a detectable level of inhibition of Fluc (7.3%), with AC_50_ values ranging from 2.7 nM to 62.2 μM, indicating that the liver stage signal for this subset of compounds related in part to their inhibition of the luciferase reporter (Supplementary Table 3).

### *P. berghei* liver stage assays identify dual-active antiplasmodial compounds

**Figure 2:**
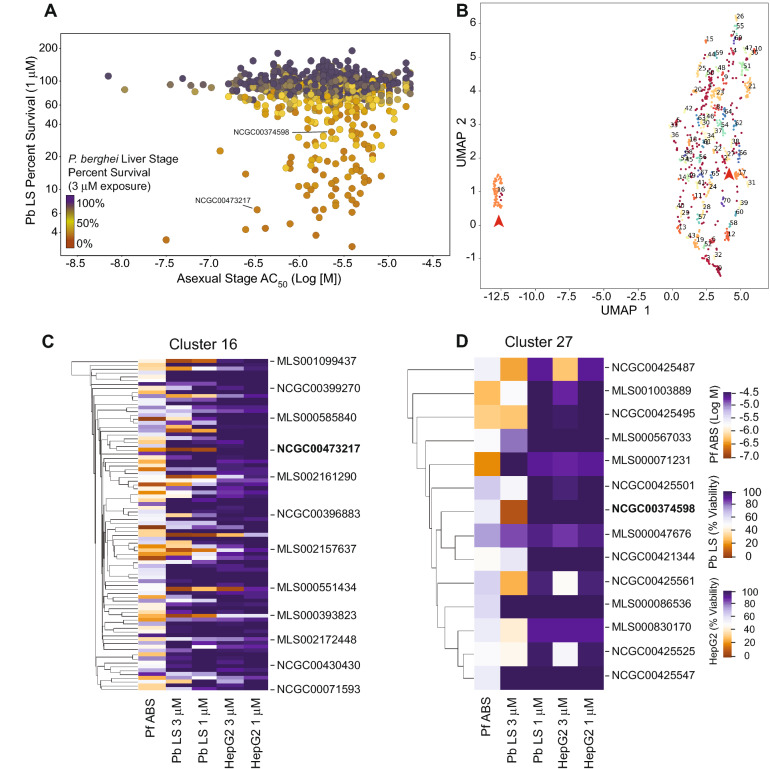
Stage-specific activity and chemical relatedness of compounds active against *P. falciparum* ABS and *P. berghei* liver stage parasites. (**A**) Multidimensional scatterplot of *P. berghei* liver stage activity compared to *P. falciparum* asexual stage inhibition. *P. berghei* liver stage percent survival (Y-axis) was assessed at a single 1 μM concentration. The color denotes the survival in the liver stage assay at a single 3 μM concentration (red, 0% survival; black 100% survival). *P. falciparum* asexual activity AC_50_ value (X-axis) was determined from qHTS 72 h in vitro growth proliferation assays. (**B**) Uniform manifold approximation and projection (UMAP) of structural similarity, based on the Tanimoto coefficient, for the 994 compounds active against *P. falciparum* ABS parasites and screened for activity against *P. berghei* liver stages. Clusters 16 and 27 are highlighted with red arrowheads. (**C**,**D**) Depiction of clusters 16 (including the thiadiazine hit NCGC00473217) and 27 (including the pyrimidine azepine hit NCGC00374598). The compounds are clustered by Tanimoto similarity, with heat map representation of the ABS activity against *P. falciparum* Dd2-B2 (shown as Log [M] concentration values), activity against *P. berghei* liver stages (Pb LS), and toxicity against HepG2 cells, tested at both 3 μM and 1 μM and represented as percentage viability. Heat maps were generated on the UMAP data projection using the software DBSCAN (version 0.24.0; https://scikit-learn.org/stable/modules/generated/sklearn.cluster.DBSCAN.html; see “Methods” section).

From the validated set, 994 ABS-active active compounds that did not demonstrate toxicity against HepG2 were manually curated and screened in an in vitro *P. berghei* liver stage assay at two concentrations (3 μM and 1 μM) in a 1536-well high-throughput assay (Figs. 1B and 2A, Supplementary Figure 1, Supplementary Table 4). These assays employed HepG2-A16-CD81EGFP cells^26^ and *P. berghei*-ANKA-GFP-Luc_CON_ parasites expressing a GFP-luciferase fusion protein^33^, and were conducted at the University of California San Diego (UCSD). Compounds were added 18 h prior to adding sporozoites, and cultures post parasite addition were then maintained an additional 48 h prior to measuring luciferase bioluminescence and inferring the percent inhibition of parasite growth. The correlation between the percent inhibition seen with compounds tested at 3 versus 1 μM was r^2^ = 0.65. This set was also screened in parallel against HepG2 cells at a concentration of 1 μM (Supplementary Table 4), with 28 compounds inhibiting HepG2 growth (above 20% compared to controls). Of the 966 Pf ABS-active compounds (with AC_50_ values < 2 μM) that did not demonstrate toxicity at 1 μM against HepG2 cells, 230 and 85 compounds demonstrated > 50% inhibition of *P. berghei* liver stage viability at 3 μM and 1 μM concentrations, corresponding to 24% and 9% of the set of 966 compounds, respectively. Based on their potency, toxicity, physicochemical properties, and novel antimalarial pharmacophore structures, we subsequently screened 81 compounds in 12-point threefold dose response assays (covering the range of 50 μM to 2.8 nM and 4 μM to 0.02 nM depending on the compound subset), with technical duplicates (Supplementary Table 5).

As a separate method of assaying for liver stage activity, we selected compounds based on their potency in the UCSD liver stage assay and their chemotype and re-screened these in a 96-well format at the Walter Reed Army Institute of Research (WRAIR). This screen used the “Inhibition of *P. berghei* Liver-Stage Development Assay” (ILSDA)^34^. A notable difference to the UCSD method is that with ILSDA the sporozoites were first added to HepG2 cells for 3 h to allow invasion, followed by washing the wells, adding the compounds, and maintaining the cultures for 48 h prior to measuring parasite luciferase levels. Unlike the UCSD assay, compounds were therefore not present at the time of sporozoite invasion. Only seven compounds were tested in both laboratories, where they demonstrated high-quality dose–response curves. We observed an overall correlation between the AC_50_ values (r^2^ = 0.67; Supplementary Fig. 2). Inter-laboratory variation of the absolute AC_50_ values for these compounds, however, was apparent. Method 1 (UCSD) yielded a large dynamic range, with a shallow dose–response slope, whereas Method 2 from WRAIR had a narrower dynamic range with a steep slope activity response (Supplementary Figure 2, Supplementary Table 5). These results were illustrated by the ATQ positive assay control and likely reflect technical differences in the respective Pb-Luc assays (Method 1 ATQ AC_50_ mean value of − 11.4 ± 0.375 Log [M], n = 11; Method 2 ATQ AC_50_ mean value of − 8.34 ± 0.01 Log [M], n = 30; Supplementary Figure 2inset). Of note, only Method 1 detected activity against sporozoites and very early and late liver stages.

### Pyrimidine azepine and thiadiazine compounds exert potent liver stage activity

**Figure 3:**
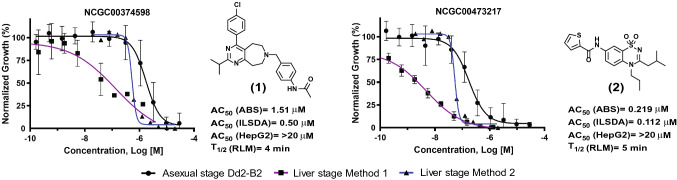
Antiplasmodial activity, toxicity and stability profiles of the prioritized hits NCGC00374598 and NCGC00473217. Compound activity profiles for NCGC00374598 (compound **1**) and NCGC00473217 (compound **2**) against *P. falciparum* asexual blood stage parasites (black line, circle), as well as *P. berghei* liver stages in either laboratory A (blue line, squares) or laboratory B (purple line, triangles). Percent inhibition is shown on the Y-axis and compound concentration (Log [M]) is shown on the X-axis. Also shown are activities against *P. falciparum* Dd2 ABS parasites, in vitro inhibition activity in the *P. berghei* liver stage development assay (ILSDA), toxicity against mammalian HepG2 cells, and half-life rat liver microsome stability (RLM) values. Mean ± SD values were derived from of at least three independent replicates.

In addition to potency and toxicity triaging of compounds representing particularly interesting chemotypes, compounds were further tested to evaluate their pharmacological properties. These tests resulted in prioritizing compounds with in vitro rat liver microsome stability greater than 4 min and parallel artificial membrane permeability assay (PAMPA) values above 500 × 10^–6^ cm/s. Additionally, preference was given to compounds exhibiting solubility above 1 μg/ml. These studies highlighted two compounds of interest for further investigation, based also on their degree of potency against asexual blood stages and liver stages. These compounds were (**1**) the pyrimidine azepine NCGC00374598, and (**2**) the thiadiazine NCGC00473217 (Fig. 3). NCGC00374598 demonstrated an AC_50_ against Dd2 ABS parasites of -5.8 ± 0.2 (Log [M] mean ± SD; corresponding to a mean AC_50_ of 1.5 µM) and liver stage AC_50_ values of − 6.2 ± 0.3 (Method 1; corresponding to a mean of 0.71 µM) and − 6.3 (Method 2; mean of 0.5 mM). NCGC00473217 had an AC_50_ against Dd2 ABS parasites of − 6.7 ± 0.4 (219 nM) against Dd2 and liver stage AC_50_ values of − 8.5 ± 0.1 (Method 1; 3 nM) and − 7.0 (Method 2; 112 nM; Fig. 3, Supplementary Tables 5 and 6). Importantly, the dose–response profiles obtained from each laboratory for these compounds mirrored their findings with the common positive control compound ATQ, confirming this drug’s activity against *Plasmodium* liver stages.

To further explore these hits, we performed chemical space clustering of compounds screened in the in vitro *P. berghei* liver stage assays. Using a UMAP projection, we identified 70 clusters with 151 singleton compounds (Fig. 2B, Supplementary Table 7)^34^. Cluster 16, consisting of 86 compounds (Fig. 2C), possessed several highly potent compounds against *P. berghei* liver stage parasites. Among these, NCGC00473217 exhibited high parasite inhibition (> 95% and > 90% inhibition at 3 μM and 1 μM, respectively), with no apparent toxicity against HepG2 in vitro (AC_50_ > 43 μM). Similarly, NCGC00374598, representing cluster 27 (Fig. 2D), displayed good potency against *P. berghei* growth in vitro (> 90% and > 60% inhibition at 3 μM and 1 μM, respectively), also with no apparent toxicity against HepG2 cells in vitro. Chemical cluster analysis and antiplasmodial activity were used to guide initial medicinal chemistry efforts to improve NCGC00473217 (see below).

We also resynthesized the pyrimidine azepine qHTS hit NCGC00374598 and confirmed its activity. However, no additional medicinal chemistry optimization was performed on this chemotype due to lack of a clear SAR from the plethora of analogs present in the primary screening set.

Assessment of the chemical space covered by compounds screened for asexual activity was compared to those triaged compounds that were selected for liver stage screening, based on the Tanimoto coefficient. As an external comparator, we also included compounds from the recent Antonova-Koch et al. study of liver stage activity (Supplementary Figure 3)^27^. Physicochemical properties were also calculated to determine differences between the chemical libraries. A trend towards decreased overall LogP profile (compound lipophilicity) was noted for our compounds selected for liver stage screening (LogP 3.0 ± 1.3; mean ± SD) compared to Antonova-Koch et al.^27^ (4.0 ± 1.3; Supplementary Figure 4).

### Resistance selection and mechanism of action studies support cytochrome b as the target of antiplasmodial pyrimidine azepine and thiadiazine compounds

In vitro selection experiments with drug-pressured cultured ABS parasites have proven effective in determining mechanisms of parasite resistance and provided important insights into the modes of action of antiplasmodial compounds of interest^35–39^. This strategy was utilized to identify the potential targets or mediators of resistance for our two prioritized hits, NCGC00473217 and NCGC00374598. Recently cloned *P. falciparum* Dd2-B2 parasites, cultured in duplicate flasks each harboring an inoculum of 2 × 10^9^ parasites, were subjected to constant drug pressure from NCGC00473217 at 5 × AC_50_ or NCGC00374598 at 3 × AC_50_. Parasites cleared in these cultures within six days. Recrudescence under pressure was observed after 14–17 days for NCGC00473217 and after 36 days for NCGC00374598, at which point we determined the concentration-responses of the recrudescent cultures. Clones were then obtained by limiting dilution and three to four clones from each flask were expanded for profiling and sequencing.

NCGC00473217 selections showed a 10 to 60-fold AC_50_ increase for clones from two independent flasks, whereas NCGC00374598-selected clones showed a threefold increase. Sequencing revealed mutations in the *cytochrome b* gene (Genbank: AAP58051.1, PlasmoDB: PfDd2_000011300) in all resistant clones from both selections. For the thiadiazine selection (NCGC00473217), whole-genome sequence (WGS) analysis of two phenotypically distinct clones from flask 2 were both found to harbor a single mutation in *cytochrome b*, encoding either F123L (> 500-fold IC_50_ shift) or V259L (60-fold IC_50_ shift). *Cytochrome b* was then PCR amplified and sequenced for the remaining clones, revealing the V259L mutation for two flask 1 clones and A122D for a separate flask 2 clone (40-fold IC_50_ shift).

**Figure 4:**
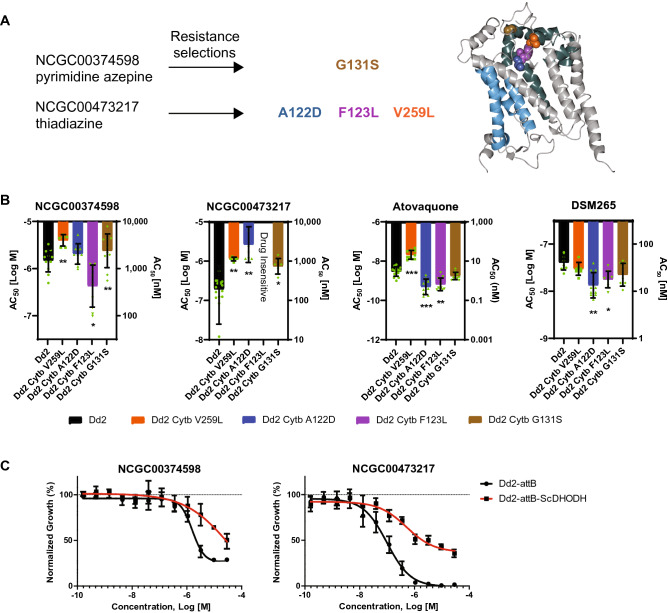
Resistance to pyrimidine azepine and thiadiazine hit compounds results in mutations in *P. falciparum cytochrome b*. (**A**) Resistance selection studies (left) with the pyrimidine azepine NCGC00374598 and the thiadiazine NCGC00473217 result in several cytochrome *b* mutations. Ribbon model (right) of the *Saccharomyces cerevisiae* cytochrome b subunit, generated using CCP4mg^78^ (version 2.10, CCP4 Molecular Graphics; https://www.ccp4.ac.uk/MG/index.html). The positions homologous to the mutations found in this study (colored spheres) cluster within or near the quinone oxidation site Qo (green^76^), and are distal to the quinone reduction site Qi (light blue^77^). Cytochrome b subunits form a dimer within the multi-component cytochrome *bc*_*1*_ complex, a mitochondrial integral membrane protein complex required for mitochondrial respiration^79^. (**B**) Comparative activity of the lead compounds NCGC00374598 and NCGC00473217 against *P. falciparum* Dd2 (B2 clone; black) and drug-selected resistant isolates (V259L (orange), A122D (blue) and F123L (purple) cytochrome b mutants were selected under NCGC00473217 pressure; cytochrome b G131S (brown) was selected under NCGC00374598 pressure). The F123L variant showed no susceptibility to NCGC00473217 up to the highest concentration tested (− 4.54 Log [M] or 29 µM). Also shown are the susceptibility responses to atovaquone, a clinically used antimalarial that targets cytochrome b and DSM265 a clinical candidate that targets the parasite dihydroorotate dehydrogenase enzyme. The y-axis denotes the AC_50_ value (shown as mean ± SD) in Log molar concentration (left axis). The right y-axis shows the AC_50_ value in nanomolar concentration. Responses to chloroquine, amodiaquine, mefloquine, ELQ-300 and artemether were similar across all parasite lines (Supplementary Table 6). Statistical variance of AC_50_ compared to Dd2 (clone B2) parental line by Student’s *t*-test; *p* < 0.05, *; *p* < 0.01, **; *p* < 0.001, ***. (**C**) *P. falciparum* parasite asexual response to NCGC00374598 or NCGC00473217 in the transgenic Dd2 strain expressing the *S. cerevisiae* dihydroorotate dehydrogenase (Dd2-attB-ScDHODH), which confers resistance to mETC and DHODH inhibitors, or the transgenic parental line Dd2-attB.

For the pyrimidine azepine (NCGC00374598) selection, genomic DNA preparations from four clones were sequenced via WGS or Sanger sequencing of PCR-amplified *cytochrome b* products. All four clones harbored a G131S mutation in *cytochrome b* (Fig. 4A). We then performed homology modeling of the *P. falciparum* cytochrome b structure, based on the known *Saccharomyces cerevisiae* cytochrome *bc*_*1*_ complex (PDB 1KYO). Based on this modeling, the mutations found in this study (colored spheres in Fig. 4A) are all suggested to reside within or near the quinone oxidation site Qo (shown in green), distal to the quinone reduction site Qi (light blue).

As resistance selection studies implicated the parasite cytochrome *bc*_*1*_ complex in mediating reduced parasite susceptibility to both NCGC00473217 and NCGC00374598, we leveraged these drug-selected parasites to test for altered drug response to a panel of antimalarial compounds. NCGC00473217-selected parasites all had significantly reduced susceptibility to the selection compound (NCGC00473217) compared to the parental Dd2-B2 lines, with the cytochrome b F123L variant parasite possessing an AC_50_ greater than the maximum concentration tested in the assay (Fig. 4B, Supplementary Table 6). The NCGC00374598-selected cytochrome b G131S variant was slightly but significantly less susceptible to this compound (− 5.5 ± 0.3 (Log [M] ± SD)) compared to the parental line (− 5.8 ± 0.2). Interestingly, there were also cross-effects between the drug-selected parasites and other compounds. For instance, the NCGC00473217 cytochrome b V259L parasite had significantly reduced susceptibility to NCGC00374598 (− 5.6 ± 0.1 vs. − 5.8 ± 0.2 for the parental line). Of note, the A122D and F123L mutant cytochrome b parasites were also significantly more susceptible to both ATQ that targets the cytochrome bc1 Q_0_ site as well as the DSM265 inhibitor that targets the functionally related mitochondria-targeted pyrimidine biosynthesis enzyme dihydroorotate dehydrogenase (DHODH)^40^. In contrast, the V259L variant parasite was significantly less susceptible to ATQ but was unchanged for DSM265 (Fig. 4B, Supplementary Table 6). There was no observed change in susceptibility to any of the other antimalarials screened, including ELQ-300 (a cytochrome bc1 Q_i_ inhibitor^41^), chloroquine, amodiaquine, mefloquine, and artemether (Supplementary Table 6).

A subset of the validated compounds was also screened against Dd2-attB and Dd2-attB-ScDHODH, the latter being a transgenic parasite line that expresses the *Saccharomyces cerevisiae* dihydroorotate dehydrogenase (ScDHODH) enzyme integrated into a genomic attB locus^42,43^. ScDHODH is a fumarate-dependent, ubiquinone-independent enzyme. Expression of ScDHODH decouples pyrimidine biosynthesis from the parasite mtETC and thus confers decreased susceptibility to compounds that target the parasite mtETC or the DHODH enzyme. In the case of NCGC00374598, we observed a shift in the parasite AC_50_ − 5.8 ± 0.1 (Log [M] ± SD) for Dd2-attB parasites compared to − 5.2 ± 0.2; *p* = 0.004 for Dd2-attB-ScDHODH parasites. A similar response shift was observed for NCGC00473217 (attB, − 7.1 ± 0.2 and attB-ScDHODH, − 6.2 ± 0.1; *p* = 0.001; Fig. 4C, Supplementary Table 8). Of the total number of compounds screened against the transgenic ScDHODH line, 27 compounds (9.4%) demonstrated a significant shift in the AC_50_ value between Dd2-attB and Dd2-attB-ScDHODH parasites, suggesting a potential mechanism of action targeting the parasite mtETC or the enzyme DHODH (Supplementary Table 8).

### Optimizing thiadiazines through medicinal chemistry

The thiadiazine hit NCGC00473217 represents an interesting chemotype in terms of antimalarial activity, ‘drug-likeness’, and what appeared to be novel chemical space based on literature searches. As such, we initiated a medicinal chemistry campaign, aimed at improving or retaining activity against ABS and liver stage *Plasmodium* parasites, while also improving the physicochemical/in vitro ADME properties (PAMPA permeability, solubility in aqueous media, and, importantly, rat liver microsomal stability). In total, 113 analogs were either synthesized or purchased (Supplementary Table 9). All compounds were analyzed in the primary screen against ABS parasites and their physicochemical properties were evaluated. The most promising compounds were also tested using the ILSDA format. From these chemical data, we identified a subset of promising thiadiazines (AC_50_ < 3 μM) (Supplementary Table 9). Most synthetic analogs, however, were either completely inactive against ABS parasites or suffered from very poor microsomal stability, a measure of host metabolic degradation of the compound.

Analogs representative of the most promising compounds, as well as other conclusions drawn from the SAR effort, are shown in Table 1. Thiadiazines maintaining the thiophenyl-amide found in the original hit were quite active against the multidrug-resistant *P. falciparum* Dd2 strain, with an AC_50_ as low as 356 nM for NCGC00532404 that contains a cyclohexylmethanamine moiety on the thiadiazine core. However, like the original hit, the compound suffered from poor microsomal stability (T_1/2_ = 3.4 min) and permeability (< 500 × 10^–6^ cm/s). A high microsomal stability (T_1/2_ > 30 min) was achieved for one thiophenyl-amide (R_1_), NCGC00521057, by truncating the thiadiazine amine (R_2_) to a methyl group. However, this resulted in decreased activity (AC_50_ = 1.85 μM) and the analog displayed low permeability. NCGC00532332, with a phenyl substituent off the thiadiazine core (R_3_), represents a thiophenyl-amide with high stability (T_1/2_ = 22.6 min), sub-micromolar activity against *P. falciparum* ABS parasites, and high permeability. A 5-fluoro-substituted thiophenyl-amides provided the most active synthetic analog in the series, NCGC00523537, with an AC_50_ of 113 nM and high permeability. This compound, however, also demonstrated poor stability. Consistent with the unsubstituted thiophene derivative, the *N*-methyl (R_2_) analog NCGC00651708 had much better stability, but again resulted in decreased activity and permeability. Changing the position or substituent appended to the thiophene-ring (at R_1_) was unsuccessful in achieving a metabolically stable and potent analog, nor was changing the connectivity to the ring. 4-Fluorophenyl amides (R_1_) were another promising subset of thiadiazines, yielding a compound (NCGC00532331) with sub-micromolar ABS activity, high microsomal stability (T_1/2_ > 30 min), and good PAMPA permeability at pH 7.4. Interestingly, all potent thiadiazines were also active against the pyrimidine azepine-resistant cytochrome b G131S mutant strain.

**Table 1:**
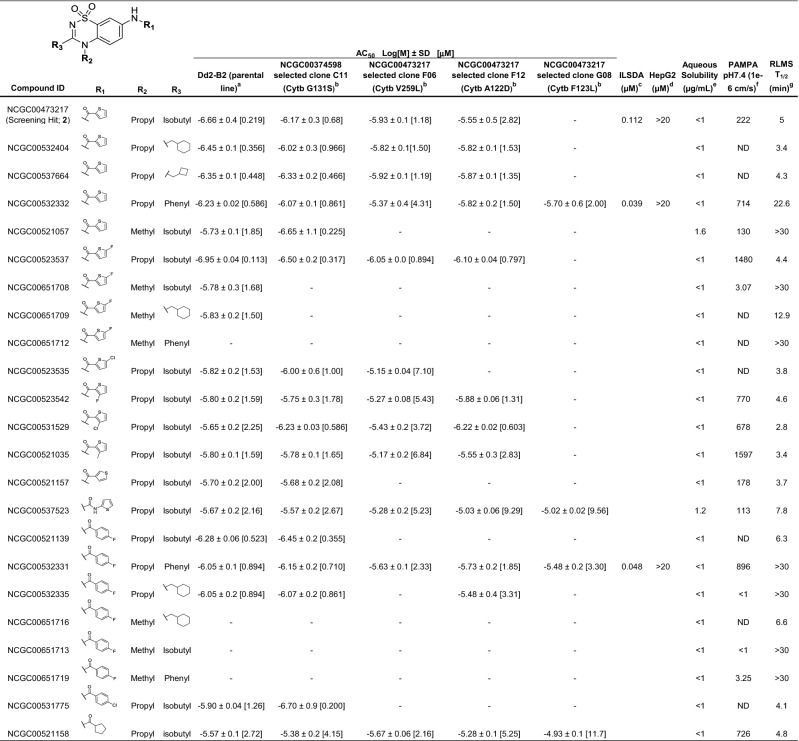
Thiadiazine analog potencies and early in vitro ADME properties.

^a^*P. falciparum* Dd2 (clone B2) activity in SYBR Green based 72 h in vitro proliferation assay; shown is the AC_50_ value expressed Log[M] ± SD, of at least three independent assays, then expressed in brackets as approximate value in µM.

^b^(-), indicates no activity or efficacy in the assay in the concentration range tested (highest concentrations of 29 µM).

^c^Inhibition of liver-stage development assay potency, 48 h assay employing a firefly luciferase reporter *P. berghei* line.

^d^Mammalian cellular toxicity assessed against HepG2 cells, 48 h compound exposure.

Pharmacokinetic modeling experiments preformed as described previously—^e^aqueous solubility^65^.

^f^Parallel artificial membrane permeability assay (PAMPA)^64^. ND (not detectable), indicates compound not detectable, could reflect insolubility, non-specific binding, compound being stuck in the lipid bi-layer, etc.

^g^Rat liver microsomal stability (RLMS) in microsomes^66^.

Aside from the original screening hit, the two most active, stable, and permeable compounds were analyzed using ILSDA for activity against liver stage parasites (Supplementary Table 5). Both optimized compounds, NCGC00532332 and NCGC00532331, were highly potent and efficacious in the ILSDA with AC_50_ values of 39 nM and 48 nM, respectively, and neither showed toxicity against HepG2 cells (AC_50_ > 20 μM). Importantly, these compounds were more stable in microsome fractions than the original thiadiazine screening hit and had similar potencies against liver stage parasites, although they were somewhat less active against ABS parasites.

## Discussion

Successes earlier this century in reducing the global burden of malaria have hit a plateau the past 5 years, highlighting the need to expand strategies and tools available for malaria treatment and control programs. A key priority is to discover and develop antimalarials that are both curative by eliminating ABS parasites and prophylactic by targeting liver stage forms. Current liver stage-active drugs are limited either by poor potency against ABS parasites and G6PD liabilities (for primaquine and tafenoquine) or can often encounter resistance (SP) or readily select for it (ATQ). Here, we undertook a large multi-point screening effort to identify potent antimalarials with activity against both *Plasmodium* ABS and liver stage parasites. This corresponds to Target Product Profile 2 as defined by the Medicines for Malaria Venture^44^.

To discover new dual-active antimalarial pharmacophores for further evaluation, we first leveraged qHTS screening against *P. falciparum* ABS parasites to identify inhibitory chemotypes. This qHTS was applied to 456,817 compounds present in our NCATS compound collection, with stringent rescreening used to identify 2223 compounds with confirmed AC_50_ values < 2 μM. Counter-screens identified 994 hits active against *P. falciparum* ABS parasites (with AC_50_ values < 2 µM) that did not show cellular toxicity against human HepG2 cells. Secondary screening utilized *P. berghei* transgenic Fluc-expressing parasites to identity compounds that also exhibited activity against liver stage parasites, with 230 and 85 compounds producing 50% growth inhibition at 3 μM and 1 μM respectively. This corresponds to 9% of our ABS-active compounds that also inhibited *P. berghei* liver stages with AC_50_ values < 1 μM. Comparable results were obtained in two prior studies that tested *P. falciparum* ABS-active compounds against *P. berghei* liver stages, both conducted at UCSD where our Method 1 assays were performed. Meister et al*.*^45^ tested 5,697 ABS-active compounds from the Novartis-GNF Malaria Box against *P. berghei* liver stages and identified 291 (5%) with IC_50_ values < 1 μM. Swann et al*.*^26^ tested 400 ABS-active compounds from the MMV Malaria Box and observed liver stage activity (with IC_50_ values < 1 μM) for 22 (5.5%). In that study, 10 of 89 (11%) ABS-active compounds from The Broad Institute DOS library were similarly potent against *P. berghei* liver stages. In a related study, Antonova-Koch et al.^27^ screened 538,273 compounds (from Charles River) against P*. berghei* liver stages, and identified 681 with an IC_50_ < 1 μM, equating to a hit rate of 0.13%. Two thirds of these compounds showed no ABS activity at concentrations up to 10 μM. Pre-screening for ABS activity therefore increases the hit rate of liver stage-active compounds by nearly two orders of magnitude.

These data are evidence of substantial differences between liver and ABS parasites in terms of their repertoires of drug targets. This is evidenced by the ABS-active, liver stage-inactive 4-aminoquinolines chloroquine, amodiaquine and piperaquine that inhibit hemoglobin degradation and heme detoxification, and artemisinin derivatives whose activation inside parasites requires heme–iron that is a product of hemoglobin degradation^5^. Conversely, the 8-aminoquinolines primaquine and tafenoquine are well known examples of drugs active against liver stages (both replicating and hypnozoite forms) and not ABS parasites.

Several established drug modes of action are nevertheless shared between liver and asexual blood stages. In a rapidly expanding list, these include folate biosynthesis targeted by the DHFR and DHPS inhibitors pyrimethamine and sulfadoxine respectively, pyrimidine biosynthesis targeted by the DHODH inhibitor DSM265, the electron transport chain targeted by atovaquone, phospholipid biosynthesis targeted by the PI4K inhibitors KDU691 and MMV390048, ER-dependent protein processing targeted by KAF156 and GN179, protein synthesis targeted by DDD107498/M5717, pre-mRNA processing targeted by the kinase inhibitor TCMDC-135051, and host cell egress and invasion events disrupted by targeting PKG^46–50^. These findings increase hope in being able to develop dual-active curative and prophylactic antimalarials. An important advance in this area is the recent development of improved methods of miniaturized liver stage cultures for both *P. falciparum* and *P. vivax*, which will expedite the analysis of liver stage-active compounds including against *P. vivax* hypnozoites^51,52^.

In our study, compounds efficacious in both the asexual blood and liver stages were prioritized for further investigation. Specifically, NCGC00374598 and NCGC00473217 were used in selection experiments with cultured *P. falciparum* Dd2 ABS parasites to identify genetic determinants of resistance, and thereby gain insight into their modes of action. NCGC00374598 selected for a G131S encoding mutation within the *cytochrome b* locus, whereas NCGC00473217 selected for parasites with the mutations A122D, F123L or V259L in this same gene. These results suggest that cytochrome b, a known antimalarial drug target, is also inhibited by these qHTS hit compounds.

This study also highlights the importance of assay design and inter-laboratory variability when assessing antiplasmodial activity, as illustrated with the two methods we employed to measure *P. berghei* liver stage inhibition. Method 1 (at UCSD) used a 1536-well format assay where the test compound is added 18 h prior to sporozoite addition, whereas Method 2 (at WRAIR) employed a 96-well format in which test compound was only added 3 h after first incubating sporozoites with hepatocytes. Only the former method would therefore identify compounds that act on sporozoites or inhibit early steps of sporozoite invasion into or initial development in HepG2 cells. The increased potency of ATQ detected in Method 1 is potentially reflective of atovaquone’s antimalarial activity during the first few hours after sporozoite invasion of the hepatocyte, a time of extensive metabolic activity during which the parasite dramatically remodels its parasitophorous vacuole membrane by degrading host proteins and inserting its own parasite-derived proteins^53^. Also, although both methods used the same original parental clone (*P. berghei* ANKA cl15cyl), they differed in the genomic site of integration of the GFP-luciferase reporter cassette and the maintenance of a selectable marker. For Method 1, the reporter and *T. gondii dhfr* selection marker were integrated into a ribosomal DNA locus, whereas for Method 2 the reporter, without an accompanying selectable marker, was integrated into the 230p locus. For both reporters, expression was driven by the constitutive *eef1α* promoter^33,34^. Nonetheless, despite these differences and the variances in the absolute AC_50_ values for the ATQ positive control and test compounds (Fig. 3, Supplementary Figure 2), there was a reasonable correlation between test compound activities (r^2^ = 0.67). This would suggest that the overall findings of reduced liver stage burden were predictive for most compounds using either methodology. Furthermore, the inclusion of the same positive control compound (ATQ) was essential to understand and rectify disparate absolute value results from each laboratory’s liver stage assays. Further studies will be important to comprehensively compare results with both assays. One benefit of using Assay 1 for the initial liver stage screen is highlighted by data with the PKG inhibitor MMV030084 that blocks parasite invasion of host cells. This compound yielded an IC_50_ of 199 nM when used to pre-treat hepatocytes 4 h prior to adding sporozoites, versus an IC_50_ > 10 μM when added 2 h post sporozoite addition^50^.

Previous work has demonstrated that the cytochrome *bc*_*1*_ complex of the mitochondrial electron transport chain is required for *Plasmodium* liver and ABS parasites^54^. This protein complex, comprised of three catalytic subunits, has been validated as a target of several antimalarial agents, including ATQ and decoquinate. Both these drugs interact with the quinone oxidation (Q_o_) binding pocket of cytochrome *bc*_*1*_^55^. Resistance selections with decoquinate earlier generated parasites with an A122T mutation in the Q_o_ binding pocket of cytochrome b^55^. Selection with NCGC00473217 resulted in parasites with an A122D mutation as well as parasites with F123L or V259L mutations, with both residues located in or near the Q_o_ binding pocket^56^. All three mutations selected with NCGC00473217 (A122D, F123L and V259L) also exhibited significantly altered susceptibility to ATQ (Fig. 4A, Supplementary Table 6).

In another study, a putative inhibitor of PfNDH2 (the quinolone RYL-552) that showed potent ABS activity was found to select for cytochrome b mutants A122T and V259L (in 106/1 parasites)^56^. The V259L mutant was also selected in Dd2 parasites pressured with ELQ-400, another potent quinolone^57^. Intriguingly, NF54-V259L clones independently selected with ATQ failed to complete mosquito development, severely impacting their mosquito infectivity or the number of oocysts produced, and thereby greatly reducing the odds of *P. falciparum* transmission to a new host^58^.

The cytochrome b G131S mutation, which also lies within the quinone oxidation site Q_o_, was obtained with NCGC00374598 in two independent selections. This mutation had previously been observed in a selection with a highly potent and selective tetracyclic benzothiazepine compound, which maintained potency against ATQ-resistant parasites^59^. In our NCGC00374598-pressured cytochrome b G131S mutant lines, ATQ potency was not compromised, suggesting a similar alteration of the Q_o_ site that perturbs NCGC00374598 binding but does not impact ATQ binding. The A122D and F123L alleles selected with NCGC00473217 also had significantly increased susceptibility to DSM265, a parasite DHODH inhibitor undergoing clinical evaluation^60^. This may reflect altered flux through the system, as the sole function of the mitochondrial respiratory chain in the asexual *Plasmodium* lifecycle is thought to be regeneration of ubiquinone for DHODH^61^. If these cytochrome b Q_o_ site mutations compromise function, this may result in reduced production of the required DHODH ubiquinone co-factor. This would decrease the relative concentration of the DHODH holoenzyme, thereby increasing parasite susceptibility to DHODH inhibitors that bind to the active enzyme conformation. Relatedly, parasites resistant to DHODH inhibitors via amplification of *pfdhodh* and nearby genes are more tolerant of inhibiting cytochrome *bc*_*1*_ function via ATQ^62^. While the exact mechanism is unknown, there is clear pharmacological/genetic cross-talk between the cytochrome *bc*_*1*_ complex and the pyrimidine biosynthesis pathway. This also suggests the possibility of combination therapies targeting both the mETC and the DHODH enzyme.

The most potent analogs against the multidrug-resistant *P. falciparum* Dd2 strain contained thiophenyl, 5-fluorothiophenyl, or 4-fluorophenyl amides at the phenyl ring (Table 1, R1) with an *N*-propyl appendage at the thiadiazine aniline-nitrogen (Table 1, R2). The substituent at the thiadiazine carbon (Table 1, R3) seemed to have less of an impact on potency for these analogs, as isobutyl, phenyl, and various aliphatic cyclic substituents were well tolerated. While these functionalities provided the most potent analogs, the majority showed very poor stability in rat liver microsome fractions. Truncating the aliphatic group at R_2_ to methyl consistently improved stability, but significantly decreased activity, suggesting the presence and location of a possible hydrophobic pocket within the currently unknown binding site. It was later shown that phenyl at R_3_ similarly increases metabolic stability without a significant loss in potency.

This study identified several novel chemotypes active against the ABS and liver stages of malaria, starting from high-throughput screening of almost half a million compounds. Hits were successfully triaged through our screening cascade, resulting in 46 non-toxic candidates with dual-stage activity. This report focused on two promising chemotypes, sharing a known mechanism of action against malaria parasites, as a proof of concept for dual-stage antimalarial drug discovery via phenotypic screening. Furthermore, we used in vitro evolution techniques to establish the drug target of our prioritized hits, for which the two cytochrome b inhibitors are reported here.

Our medicinal chemistry efforts resulted in the identification of two thiadiazines, NCGC00532331 and NCGC00532332, with mean AC_50_ values against *P. falciparum* Dd2 ABS parasites of 894 and 586 nM, respectively, and activity in a *P. berghei* liver stage model of < 50 nM, as determined in ILSDA. Neither compound was cytotoxic against HepG2 cells and both had favorable rat liver microsomal stability and PAMPA permeability. Similar to all other considerably active compounds, both suffered from poor aqueous solubility. However, we are optimistic that these compounds could be formulated for potential in vivo administration by using an appropriate salt at the slightly basic nitrogen of the thiadiazine core. Interestingly, the optimized thiadiazine compound NCGC00532331 maintained potency against the pyrimidine azepine resistant strain, cytochrome b G131S, suggesting a potentially altered binding mode compared to the hit compound NCGC00473217. Pharmacokinetic experiments are required to investigate ADME profiles, which will shed light on potential liabilities that could result from the high lipophilicity of this series. Further optimization of thiadiazines and their in vivo assessment in rodent models will be important to assess their potential as a new series of causal therapeutics.

## Methods

### Parasite quantitative high-throughput screening (qHTS)

The *P. falciparum* parasite Dd2 line was cultured in vitro using standard conditions^63^. Briefly, parasites were maintained in 2% human O^+^ erythrocytes (Interstate Blood Bank, Memphis, TN) in RPMI-1640 medium (Life Technologies, Grand Island, NY) supplemented with 0.5% Albumax II (Life Technologies), 2 mM l-glutamine, 50 mg/L hypoxanthine, 25 mM HEPES, 0.225% NaHCO_3_, 24 mM sodium bicarbonate, and 10 μg/ml gentamycin. Tissue culture flasks and assay plates were incubated at 37 °C under a gas mixture of 5% CO_2_, 5% O_2_ and 90% N_2_. Methods for the SYBR Green-I qHTS, calculation of AC_50_ (50% of the normalized compound response relative to the maximum response), and definition of curve classes have been described^30,64–66^. qHTS assays were performed in a single replicate with a 7- to 11-point dose response format, and follow-up screens with 3–11 independent 11-point response assays. Significant differences were assessed using two-sided Student’s t-tests. All HTS assays were stopped and read at 72 h. Percent response values represent relative growth as judged by SYBR Green-I fluorescence intensity values normalized to controls. In addition to our primary screening efforts, the hits from our prior unpublished ABS screening of the MLSMR collection (PubChem AID 488774), using a previously published *P. falciparum* Firefly luciferase reporter line^67^, were also used to identify novel leads for testing against *P. berghei* liver stages.

To assess toxicity against mammalian cells, 2 × 10^3^ HepG2 cells per well were dispensed into white solid-bottom 1536-well plates in DMEM assay medium using a multidrop combi dispenser in a 5 µL volume. Compounds dissolved in DMSO were transferred to the assay plate via pin tool (23 nL volume; Waco, San Diego, CA), with a final concentration range of 1.6 nM to 57 μM. Each plate included media only (no cells) and solvent-only control wells in columns 1–4. The plates were incubated for 48 h at 37 °C, and one volume of CellTiter-Glo assay reagent (Promega, Madison, WI) was added using a BioRAPTR FRD (Beckman Coulter, Brea, CA). Cell viability was measured using a ViewLux µHTS Microplate Imager (PerkinElmer, Waltham, MA).

Compound activity against the *Photinus pyralis* luciferase (Firefly luciferase, Fluc) reporter utilized for the liver stage assay was determined as previously described using 10 nM Fluc (EC 1.13.12.7) and 10 μM of each substrate (ATP and D-luciferin)^68^. Compounds (23 nL) were transferred to the plates by a pin tool in the concentration range of 1.6 nM to 57 μM, with DMSO and a no-luciferase sample as background signal controls. Luminescence was detected using ViewLux µHTS Microplate Imager.

To evaluate whether compounds inhibit the *P. falciparum* mitochondrial electron transport chain (mtETC), we utilized previously generated transgenic parasites that express the *Saccharomyces cerevisiae* dihydroorotate dehydrogenase (Dd2-attB-ScDHODH) or the isogenic parental line (Dd2-attB)^69^. The overexpression of the fumarate-dependent yeast DHODH confers resistance to compounds that inhibit the parasite mETC, or the parasite DHODH enzyme that depends on ubiquinone provided by the parasite mETC. Assays were performed in three independent assays on a subset of compounds, and differences between the Dd2-attB parental line and the ScDHODH transgenic line were analyzed using two-sided Student’s t-tests.

### *P. berghei* liver stage and HepG2 toxicity assays

The Pb-Luc high-throughput screen was performed as previously described^26^, with some modifications. Briefly, HepG2-A16-CD81EGFP cells (3 × 10^3^) in 5 μL of assay medium (DMEM without Phenol Red (Life Technologies), 5% FBS, and 5 × Penn-Strep Glutamine (Life Technologies)) were seeded into 1536-well, white, solid-bottom plates (custom GNF mold, ref# 789173-F, Greiner Bio-One). Seeding was performed 20 to 26 h prior to sporozoite inoculation. 18 h before adding sporozoites, test compounds (diluted in DMSO) were transferred into assay plates with either with a PinTool (Fujifilm Wako Automation, San Diego, CA) or an Acoustic Transfer System (EDC Biosystems, Fremont, CA). Compounds were added to desired final assay concentrations of 3 µM and 1 µM for single point screens and 50 μM or 4 μM top concentrations for dose–response reconfirmations (depending on the compound subset). Atovaquone (plated at a 12-point serial dilution, starting at a maximum of 0.5 μM) and DMSO were used as positive and negative controls respectively. *P. berghei*-ANKA-GFP-Luc_CON_ (Pb-Luc_con_^34^) sporozoites were obtained from freshly dissected, infected *Anopheles stephensi* salivary glands, filtered through 20 μm nylon net filters (Steriflip, MilliporeSigma, Burlington, MA), and counted in a hemocytometer. Their concentration was adjusted to 200 sporozoites per μL in assay medium (DMEM without Phenol Red (Life Technologies), 5% FBS, and 5 × Penn-Strep Glutamine (Life Technologies)). The HepG2-A16-CD81EGFP cells were then infected with 1 × 10^3^ sporozoites per well (5 μL) using a single tip Bottle Valve liquid handler (GNF). Plates were centrifuged for 3 min at room temperature and at 330 × *g* (Eppendorf 5810 R centrifuge) on normal acceleration and brake setting. The HepG2-A16-CD81EGFP cell designated for toxicity studies were left uninfected, with 5 μL of additional assay media added to each well to maintain equal concentrations of compounds with Pb-Luc_con_ infected plates. Plates were incubated at 37 °C for 48 h in 5% CO_2_ with high humidity to minimize media evaporation and edge effects.

After a 48 h incubation, liver stage parasite growth and the viability of HepG2-A16-CD81EGFP cells were quantified using bioluminescence as follows: medium was removed by spinning the inverted plates at 150 × *g* for 30 s, and a MicroFlo liquid handler (BioTek, Winooski, VT) used to dispense 2 μL per well of BrightGlo reagent (Promega) to quantify Pbluc liver stage parasites or CellTiterGlo reagent (Promega) (diluted 1:2 with deionized water) to quantify HepG2-A16-CD81EGFP cell viability. Immediately after adding the reagent, the luminescence was measured on an Envision Multilabel Reader (PerkinElmer). AC_50_ values were calculated using the same methodology as indicated above for the drug response with ABS parasites.

### Inhibition of liver-stage development assay (ILSDA)

Our inhibition of liver-stage development assay (ILSDA) is a modification of a previously published in vitro method^34^. In preparation for liver cell culture, we first coated sterile, white 96-well culture plates with Cell Attachment Matrix (ECL) diluted in HBSS using the Tecan Freedom EVO robotics (Tecan, Morrisville, NC) system. The plates were incubated for 30 min at 37 °C and 5% CO_2_. HepG2 cells, cultured in complete Minimal Essential Medium (MEM) supplemented with 10% FBS, were dispensed at a final concentration of 2.5 × 10^4^ HepG2 cells per well and incubated at 37 °C and 5% CO_2_ for 24 h. After incubation, each well received 1 × 10^4^ sporozoites, harvested from *An. stephensi* mosquitoes that were previously infected with a transfected luciferase-expressing *P. berghei* strain. Plates were then incubated for 3 h at 37 °C and 5% CO_2_ to allow for sporozoite invasion of the HepG2 cells. The plates were then washed 3 times with MEM media to remove any remaining extracellular sporozoites. Next, 100 μL of test compounds, serially diluted twofold, were added to each well. ATQ (Sigma) was used as the control compound. Plates were incubated for 48 h at 37 °C and 5% CO_2_. After 48 h, a luciferin solution (Perkin Elmer), diluted to 150 μg/ml, was added to each well. Plates were read in a Perkin Elmer plate reader. AC_50_ values were calculated using the same methodology as indicated above for ABS parasites.

### Resistance selection studies

To select for *P. falciparum* resistance to NCGC00473217 (a thiadiazine) and NCGC00374598 (a pyrimidine azepine), duplicate flasks containing 200 mL of 2 × 10^9^ Dd2 (clone B2) ABS parasites at 2.5% parasitemia were pressured with 3 to 5 times the ABS Dd2-B2 AC_50_ value. Freshly prepared media containing the compound of interest was refreshed daily for the first 7 days, while monitoring the cultures to confirm clearance of sensitive parasites and thereby avoiding overgrowth. Fresh uninfected RBCs (0.5% hematocrit) were added on day 7 and media was changed every other day. From day 14 onwards, the culture volume was reduced by 25% each week and fresh RBCs were added (at 1% final hematocrit) to sustain a 4% total hematocrit, until recrudescence was observed. Resistant parasites were maintained under drug media at a volume of 50 mL until a high parasitemia (> 5%) was achieved. Aliquots of the bulk resistant cultures were frozen in cryovials before cloning by limiting dilution in 96-well plates (seeded at 0.25 or 0.5 parasites/well)^70^. After 3 weeks of cloning, we screened for positive wells by flow cytometry with SYBR Green-stained cells. Individual clones (2–3 clones per original selection flask) were expanded to 100 mL volumes at > 5% parasitemia. Parasites were harvested by saponin lysis at the trophozoite stage and genomic DNA was extracted using the QIAamp DNA Blood Midi Kit (Qiagen, Germantown, MD)^71^.

### Whole-genome sequencing

This methodology, described below, has been previously reported by our group^50^. Libraries for the resistant clones and the sensitive Dd2-B2 parent were prepared using the Illumina TruSeq DNA PCR-free kit according to the manufacturer’s protocol. Briefly, two µg of genomic DNA were sheared to mean fragment sizes of 550 bp, end-repaired, adenylated on 3′ ends, and ligated with adaptors. The samples were pooled and multiplexed on a MiSeq flow cell to generate 2 × 300 bp paired end reads. Sequence data were aligned to the *P. falciparum* 3D7 genome (PlasmoDB version 11.0) using BWA (Burrow-Wheeler Alignment). Samtools and Picard were used to remove reads that did not map to the reference genome, duplicate reads flagged by MarkDuplicate (Picard Tools, Broad Institute), and low-quality reads. The reads were realigned around indels using GATK RealignerTargetCreator, and base quality scores were recalibrated using GATK Table-Recalibration. GATK UnifiedGenotyper (minimum base quality score ≥ 17) was used to identify all possible variants in clones. These variants were filtered based on quality scores (variant quality as function of depth QD > 1.5, mapping quality > 50 and read depth (depth of read > 5) to obtain high quality single nucleotide polymorphisms that were annotated using snpEFF. The list of variants from the resistant clones were compared against the Dd2-B2 parent to identify non-synonymous single nucleotide polymorphisms present exclusively in the resistant clones at > 80% alternate allele frequency. Copy number variation analysis was performed using the BicSeq package, by comparing the read counts of the resistant clones against the Dd2-B2 parent.

### Cluster analysis

Computational chemistry calculations were carried out using the Palantir Foundry platform (https://www.palantir.com/palantir-foundry; Palantir Technologies, Palo Alto, CA). Tanimoto similarities between all pairs of compounds, represented as Morgan Fingerprints, were calculated with the rdkit python package (https://www.rdkit.org/). Chemical space plots were generated first through principal component analysis (PCA) on these fingerprints using the scikit learn package (https://scikit-learn.org/stable/), and separately through uniform manifold approximation and projection (UMAP) using the umap-learn package (https://umap-learn.readthedocs.io/en/latest/). Compound clustering by chemical space was conducted on the UMAP data projection using DBSCAN (version 0.24.0; https://scikit-learn.org/stable/modules/generated/sklearn.cluster.DBSCAN.html). Physical properties of compounds were calculated using the rdkit package. Structures were de-salted and sanitized, with canonical tautomers selected (https://www.rdkit.org/docs/RDKit_Book.html#molecular-sanitization) before property calculation (including molecular weight, polar surface area, LogP and hydrogen bond donors and acceptors).

### Compound synthesis

qHTS hits NCGC00374598 and NCGC00473217 were obtained from commercially available screening libraries. The pyrimidine azepine scaffold (NCGC00374598) has been previously described as a 5-HT_2A_ antagonist^72^, while the thiadiazine hit chemotype (NCGC00473217) does not appear to have been described in the literature, although > 300 analogs were available from commercial screening libraries based on a SciFinder search (https://scifinder.cas.org).

The thiadiazine hit, NCGC00473217, was synthesized starting from commercially available 2-chloro-5-nitrobenzenesulfonamide (Supplementary Methods, Scheme 1). The syntheses of all analogs followed the same general protocol. Briefly, an aliphatic amine was added to 2-chloro-5-nitrobenzenesulfonamide via nucleophilic aromatic substitution with triethylamine in acetonitrile at reflux. The resulting amine SI-1 was then acylated in the presence of stoichiometric DMAP, giving the corresponding amide SI-2. The use of stoichiometric amounts of DMAP proved critical for high yields, as other conditions (catalytic DMAP) or bases (triethylamine, Hunig’s base) provided mixtures of amide, acylsulfonamide, and bis-acylation products that proved difficult to separate. Cyclization to the corresponding thiadiazine SI-3 was achieved using Eaton’s reagent in refluxing toluene. The nitro group was then reduced with iron powder and ammonium chloride in aqueous ethanol. The resulting aniline SI-4 was acylated with the corresponding acid chloride in the presence of pyridine in DCM, with mild heating, to afford the final compound **1** (NCGC00473217).

The pyrimidine azepine qHTS hit NCGC00374598 was resynthesized and activity confirmed using a previously reported methodology^65^. However, no additional medicinal chemistry optimization was performed on this chemotype due to lack of a clear SAR from the plethora of analogs included in the screen. Pharmacokinetic modeling experiments, parallel artificial membrane permeability assay (PAMPA), solubility, and rat liver microsome stability assessment were performed on all synthetic compounds, as previously described^73–75^.

### Molecular modeling

The *P. falciparum* cytochrome b amino acid sequence was aligned to the *Saccharomyces cerevisiae* sequences used in PDB 1KYO as shown for the Qo site^76^ and Qi site^77^. Molecular imaging used CCP4mg (version 2.10, CCP4 Molecular Graphics)^78^.

## Supplementary Information

Supplementary Information.

Supplementary Tables.

## Data Availability

Screening and validation data sets have been deposited into Pubchem AID 488774, 1347417 and 1347416.

## Requests for materials

Questions related to the compound screens and parasite cell lines should be addressed to Anton Simeonov (asimeono@mail.nih.gov) and David Fidock (df2260@cumc.columbia.edu), respectively.
