# Supplementary material for: Chemoprotective antimalarials identified through quantitative high-throughput screening of Plasmodium blood and liver stage parasites
Source: Sci Rep. 2021 Jan 22;11:2121. doi: 10.1038/s41598-021-81486-z (PMC7822874; doi:10.1038/s41598-021-81486-z)
Supplement: Supplementary file 1 — Supplementary Information. [file 41598_2021_81486_MOESM1_ESM.pdf]

Supplementary Material

Chemoprotective antimalarials identified through quantitative high-throughput screening of *Plasmodium* blood and liver stage parasites

Dorjbal Dorjsuren[1,†], Richard T. Eastman[1,†], Kathryn J. Wicht[2,a], Daniel Jansen[1], Daniel C. Talley[1], Benjamin A. Sigmon[1], Alexey V. Zakharov[1], Norma Roncal[3], Andrew T. Girvin[4], Yevgeniya Antonova-Koch[5,b], Paul M. Will[1], Pranav Shah[1], Hongmao Sun[1], Carleen Klumpp-Thomas[1], Sachel Mok[2], Tomas Yeo[2], Stephan Meister[5,c], Juan-Jose Marugan[1], Leila S. Ross[2], Xin Xu[1], Elizabeth A. Winzeler[5], David J. Maloney[1,d], Ajit Jadhav[1], Bryan T. Mott[1], Richard J. Sciotti[3], Norman C. Waters[3], Robert F. Campbell[3], Wenwei Huang[1], Anton Simeonov[1,*] & David A. Fidock[2,6,*]

[1]Division of Preclinical Innovation, National Center for Advancing Translational Sciences, National Institutes of Health, Rockville, MD 20850, USA; [2]Department of Microbiology and Immunology, Columbia University Irving Medical Center, New York, NY 10032, USA; [3]Experimental Therapeutics Branch, Walter Reed Army Institute of Research, Silver Spring, MD 20910, USA; [4]Palantir Technologies, Washington, DC 20007, USA; [5]Department of Pediatrics, Pharmacology & Drug Discovery, School of Medicine, University of California San Diego, La Jolla, CA 92093, USA; [6]Division of Infectious Diseases, Department of Medicine, Columbia University Irving Medical Center, New York, NY 10032, USA.

Current affiliations: [a]Drug Discovery and Development Centre (H3D), University of Cape Town, Rondebosch 7701, South Africa; [b]Calibr, The Scripps Research Institute, La Jolla, CA 92037, USA; [c]Beckman Coulter Inc., Carlsbad, CA 92010, USA; [d]Veralox Therapeutics Inc., Frederick, MD 21704, USA

[†]These authors contributed equally to this work.

*Co-corresponding authors.

Table of contents (note: Supplementary Tables are provided in a separate Excel file)

Supplementary Figure 1. Multidimensional scatterplot of *P. berghei* liver stage activity compared to *P. falciparum* asexual stage inhibition, limited to those compounds with >10% inhibitory activity at the 1 µM concentration.

Supplementary Figure 2. Comparison of compound activity in *P. berghei* liver stage dose-response activity in independent laboratory assays.

Supplementary Figure 3. Uniform manifold approximation and projection of chemical relatedness, based on Tanimoto distance, for all 456,817 compounds evaluated for asexual stage activity.

Supplementary Figure 4. Calculated chemical properties of all 456,817 compounds evaluated for asexual stage activity.

Chemistry general methods

Supplementary Table 1. Details of validation set of 4,253 compounds active against *P. falciparum* Dd2 asexual blood stage parasites.

Supplementary Table 2. HepG2 mammalian toxicity assessment for the validation set of asexual blood stage-active compounds.

Supplementary Table 3. Firefly luciferase counter-screen for the validation set of asexual blood stage-active compounds.

Supplementary Table 4. *P. berghei* liver stage assessment, 2 concentration points.

Supplementary Table 5. *P. berghei* liver stage inhibition assessment, dose response.

Supplementary Table 6. Drug susceptibilities against *P. falciparum* asexual blood stage parasites for parental and cytochrome b-mutated lines.

Supplementary Table 7. Compound cluster activity analysis.

Supplementary Table 8. Yeast DHODH differential activity assessment.

Supplementary Table 9. Assessment of compound activity against drug-resistant cytochrome b-mutant lines and properties of medicinal chemistry analogs.

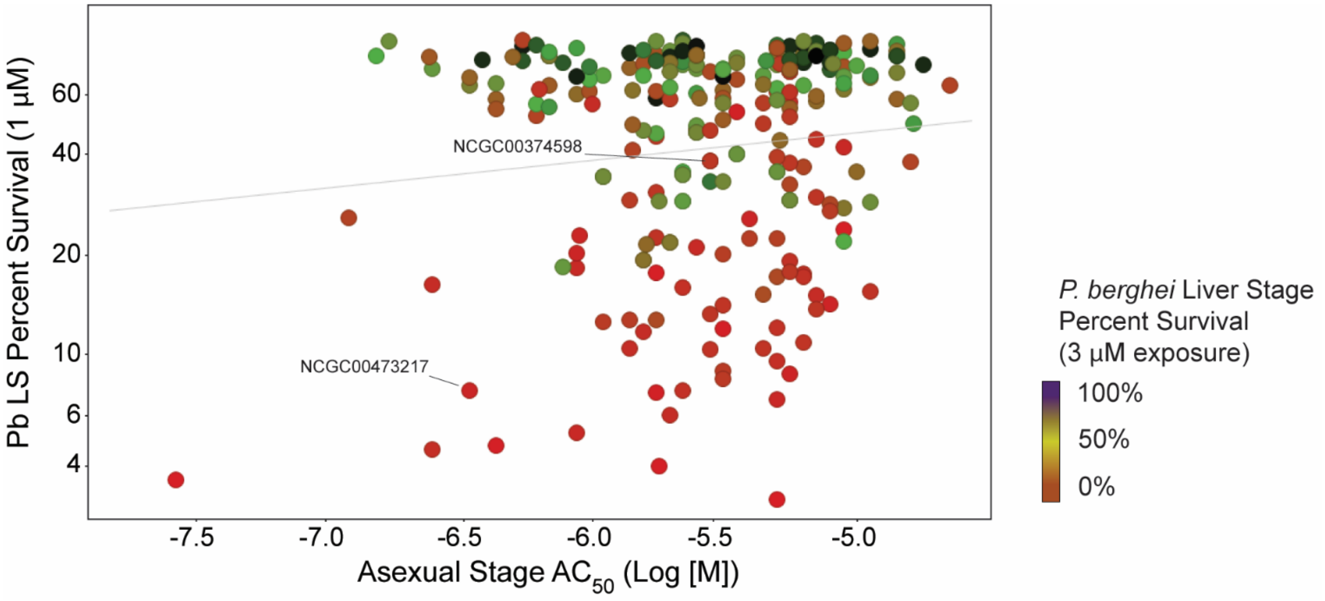

Supplementary Figure 1. Multidimensional scatterplot of *P. berghei* liver stage activity compared to *P. falciparum* asexual stage inhibition, limited to those compounds with >10% inhibitory activity at the 1 µM concentration. *P. berghei* liver stage percent survival (Y-axis) was assessed at a single 1 µM concentration. The color denotes the survival in the liver stage assay at a single 3 µM concentration (red, 0% survival; black 100 % survival). *P. falciparum* asexual activity AC_{50} value (X-axis) was determined from qHTS 72 hr *in vitro* growth proliferation assays. Compounds NCGC00374598 and NCGC00473217 are indicated.

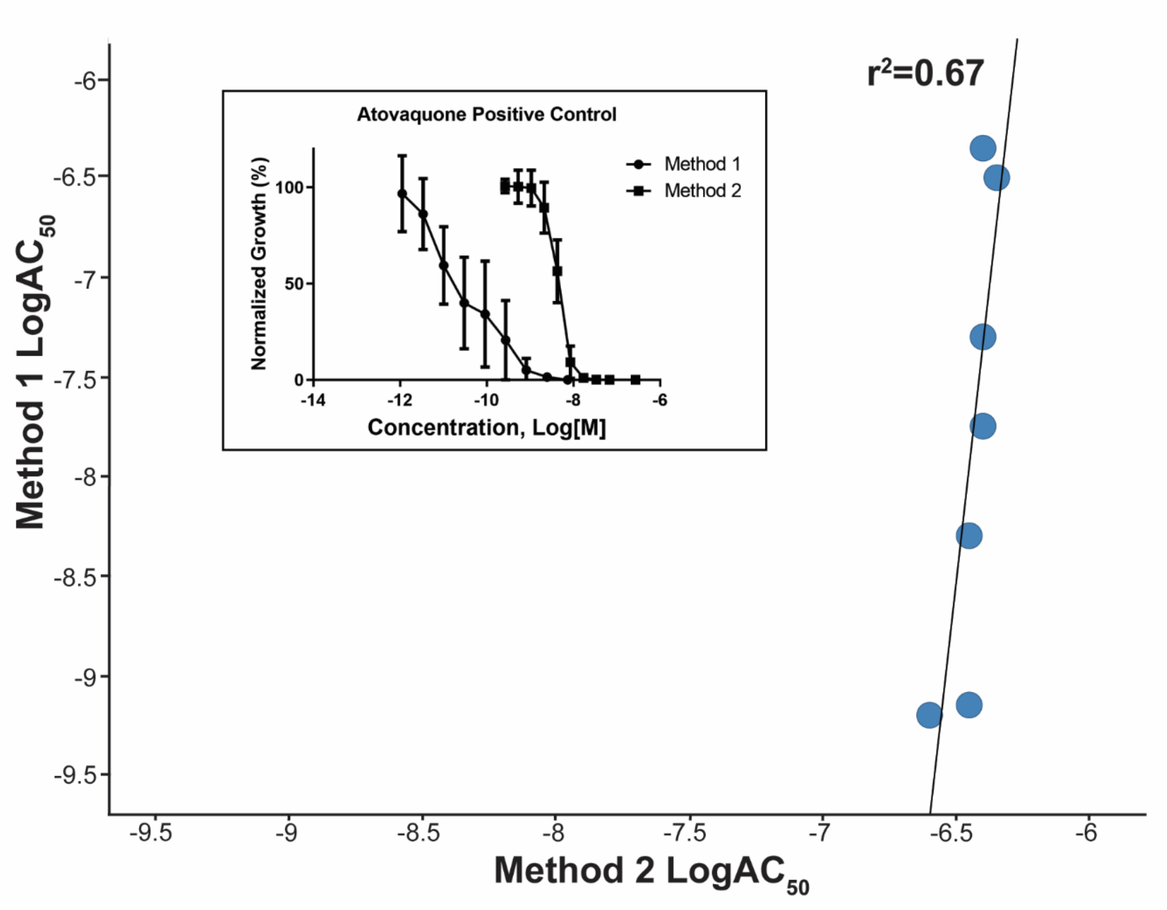

Supplementary Figure 2. Comparison of compound activity in *P. berghei* liver stage dose-response activity in independent laboratory assays. Shown are high-quality compounds, in both laboratories. Inset shows the atovaquone positive control *P. berghei* liver stage response for each independent laboratory. Shown is the mean and standard error.

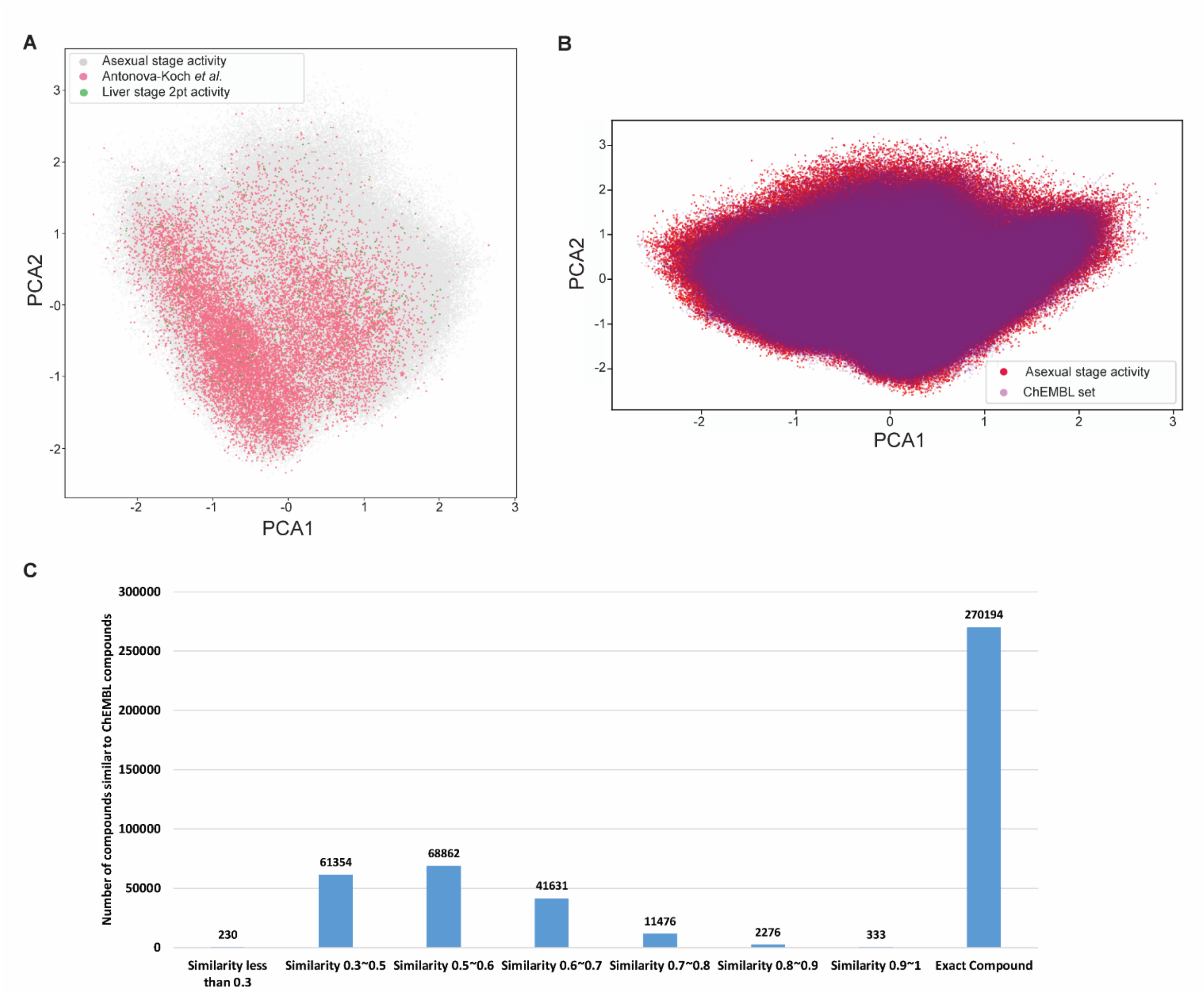

Supplementary Figure 3. (A) Principal Component Analysis (PCA) plot of chemical relatedness, based on Tanimoto distance, for all 456,817 compounds evaluated for asexual stage activity (shown in grey). For comparison, the recent compound set evaluated for *P. berghei* liver stage activity by Antonova-Koch *et al*. is shown in pink[2]. The 994 *P. falciparum* asexual stage active compound screened for activity against the *P. berghei* liver stage are shown in green. **(B)** PCA plot of the chemical relatedness, based on Tanimoto distance, for all 456,817 compounds evaluated for asexual stage activity (shown in red) and the ChEMBL compound collection (1,678,393; shown in light purple) to illustrate the chemical space covered by our collection. **(C)** Histogram breakdown of the Taminoto distance similarity between the asexual stage activity compounds and the ChEMBL compound collection.

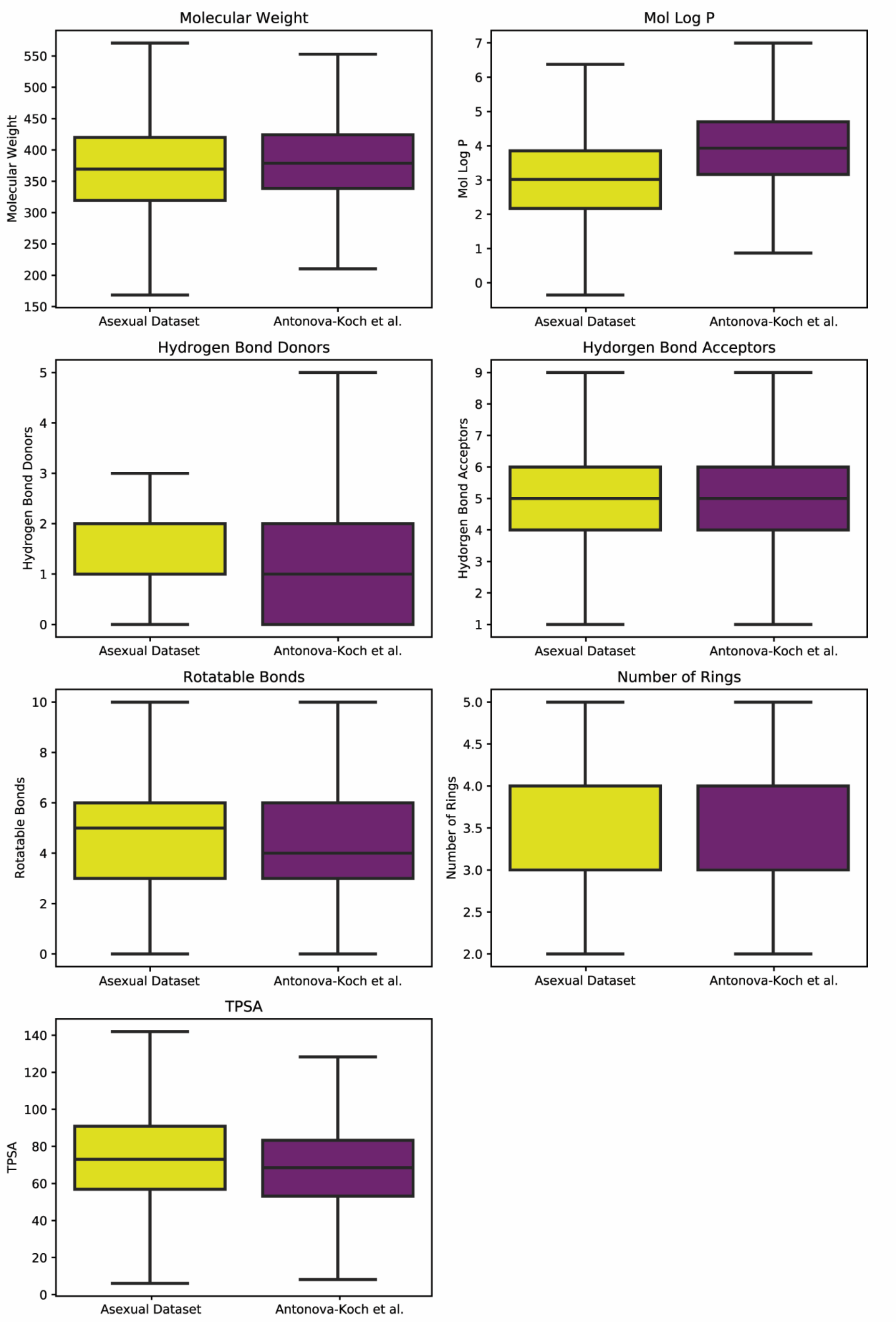

Supplementary Figure 4. Calculated chemical properties of all 456,817 compounds evaluated for asexual stage activity (shown in yellow). Compared to the recent compound set evaluated for *P. berghei* liver stage activity by Antonova-Koch *et al*. (shown in purple) [1]. Shown is median, and the first and third quartiles.

Chemistry General Methods

All air and/or moisture sensitive reactions were performed under positive pressure of nitrogen or argon with oven-dried glassware. Anhydrous solvents and bases such as dichloromethane, *N,N*-dimethylformamide (DMF), acetonitrile (CAN), ethanol (EtOH), dimethylsulfoxide (DMSO), 1,4-dioxane, diisopropylethylamine (DIPEA), and triethylamine (TEA) were purchased from Sigma-Aldrich. Palladium catalysts were purchased from Strem chemicals. All other reagents were purchased from Sigma-Aldrich or Combi-Blocks in the highest available purity and used as received. Normal phase flash chromatography was performed using an ISCO system and GOLD silica columns. Reverse phase preparative purifications were performed on a Waters semi-preparative HPLC system using an Agilent XDB C18 PrepHT column (5 micron, 30 x 100 mm) at a flow rate of 45 mL/min. The mobile phase consisted of acetonitrile and water (each containing 0.1% trifluoroacetic acid). A gradient of 10% to 50% acetonitrile over 8 minutes was used during the purification. Fraction collection was triggered by UV detection (220 nm). Analytical analysis was performed on an Agilent LCMS (Agilent Technologies, Santa Clara, CA) using either **Chemical Method 1** (for monitoring reaction progress) or **Chemical Method 2** (QC for final compounds/intermediates). We note that some of these chemistry general methods have been previously reported[1].

Chemical Method 1: A 3-minute gradient of 4% to 100% Acetonitrile (containing 0.025% trifluoroacetic acid) in water (containing 0.05% trifluoroacetic acid) was used with a 4.5 minute run time at a flow rate of 1 mL/min. An Agilent ZORBAX Eclipse XBD-C18 column (3.5 micron, 3 x 75 mm) was used at a temperature of 50° C.

Chemical Method 2: A 7-minute gradient of 4% to 100% Acetonitrile (containing 0.025% trifluoroacetic acid) in water (containing 0.05% trifluoroacetic acid) was used with an 8 minute run time at a flow rate of 1 mL/min. An Agilent ZORBAX Eclipse XBD-C18 column (3.5 micron, 3 x 75 mm) was used at a temperature of 50° C.

UV-Absorption spectra during purification were collected using an Agilent Diode Array Detector for both Method 1 and Method 2. Mass determination was performed using an Agilent 6130

mass spectrometer with electrospray ionization in positive mode. ^{1}H NMR spectra were recorded on Varian 400 MHz spectrometers. Chemical shifts for final compounds are reported in ppm with undeuterated solvent (DMSO-d_6 at 2.50 ppm) as internal standard for DMSO-d_6 solutions. Chemical shifts for intermediate compounds are reported in ppm with undeuterated solvent ($CDCl_3$ at 7.26 ppm) as internal standard or using DMSO as described for final compounds. All analogs tested in the biological assays have purity greater than 95%, based on both analytical methods. High resolution mass spectrometry was recorded on Agilent 6210 Time-of-Flight LC/MS system.

Scheme 1: Thiadiazine synthesis.

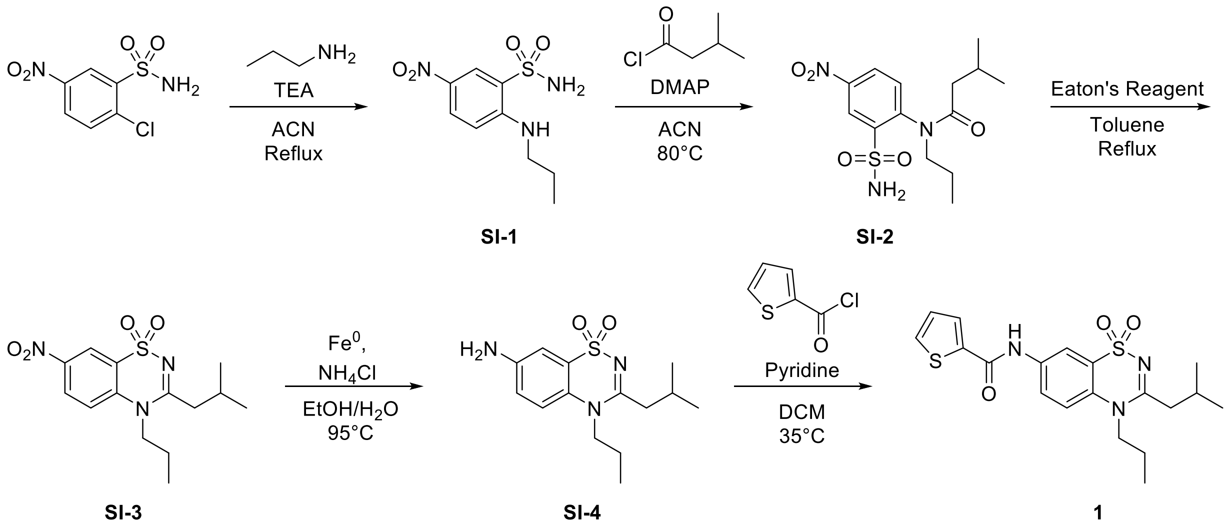

Synthesis and characterization:

All thiadiazine analogs were synthesized using the same general methodology, as outlined in Scheme 1. The detailed synthesis of representative thiadiazine 1 is described below:

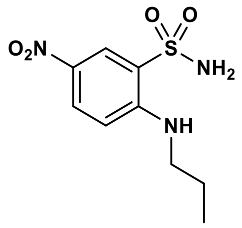

5-Nitro-2-(propylamino)benzenesulfonamide (SI-1):

A mixture of 2-chloro-5-nitrobenzenesulfonamide (9 g, 38.0 mmol, 1 eq.), propan-1-amine (4.67 ml, 56.9 mmol, 1.5 eq) *(or the appropriate amine)*, triethylamine (10.32 ml, 74.0 mmol, 1.95 eq), in Acetonitrile (90 ml) was heated to 90°C for 2 hours, until judged to be complete by LC-MS. The mixture was concentrated *in vacuo* to about 15-20 mL of yellow oil. H_2O was then added and a yellow solid formed slowly upon stirring. The solid was collected via vacuum filtration, washed with H_2O, and dried *in vacuo* to provide 9.1g (92% yield) of **SI-1**. LC-MS t1 = 3.06 min, M+H = 260 ^{1}H NMR (400 MHz, Chloroform-d) δ 8.73 (d, J = 2.7 Hz, 1H), 8.26 (dd, J = 9.4, 2.6 Hz, 1H), 6.80 – 6.74 (m, 1H), 6.66 (brs, 1H), 4.86 (s, 2H), 3.27 (td, J = 7.0, 5.1 Hz, 2H), 1.76 (m, 2H), 1.06 (t, J = 7.4, 3H).

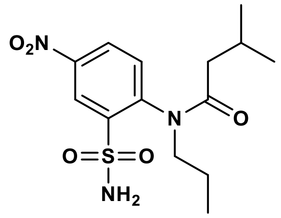

3-Methyl-N-(4-nitro-2-sulfamoylphenyl)-N-propylbutanamide (SI-2):

To a solution of 5-nitro-2-(propylamino)benzenesulfonamide (5.19 g, 20 mmol) and DMAP (2.455 g, 20.10 mmol) in Acetonitrile (200 ml), 3-methylbutanoyl chloride (2.500 ml, 20.10 mmol) (*or the appropriate acid chloride*) was added and the reaction was heated to 80°C for 4 hr. The mixture was then concentrated *in vacuo*, diluted with ethyl acetate, and the organic phase was washed with 1M HCl and brine, dried over sodium sulfate, filtered, and concentrated *in vacuo* to provide **SI-2** (6.8 g, 19.80 mmol, 99 % yield), which was used without further purification. LC-MS t1 = 3.44 min, t2 = 5.61 min. ^{1}H NMR (400 MHz, Chloroform-d) δ 8.69 (d, J = 2.6 Hz, 1H), 8.20 (dd, J = 9.5, 2.6 Hz, 1H), 7.29 (brs, 1H), 6.73 (d, J = 9.5 Hz, 1H), 3.34 – 3.23 (m, 2H), 2.15 (m, 2H), 2.06 (dt, J = 13.6, 6.9 Hz, 1H), 1.78 (sex, J = 7.3 Hz, 2H), 1.06 (t, J = 7.4 Hz, 3H), 0.92 (d, J = 6.5, 6H).

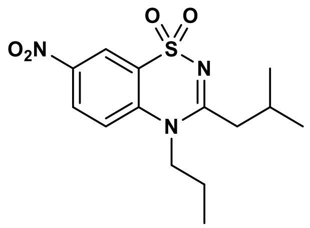

3-Isobutyl-7-nitro-4-propyl-4H-benzo[e][1,2,4]thiadiazine 1,1-dioxide (SI-3):

To a solution of 3-methyl-N-(4-nitro-2-sulfamoylphenyl)-N-propylbutanamide (5.3 g, 15.43 mmol) in Toluene (77 ml), Eaton's Reagent (8.16 ml, 51.4 mmol) was added. The reaction was then heated to 130°C for 6 hours. The reaction was cooled to RT and slowly poured into an aqueous solution of sodium bicarbonate. The mixture was extracted with ethyl acetate (3 x 100 mL), separated, and washed with brine, then dried over sodium sulfate, filtered, and concentrated *in vacuo*. The residue was then purified via automated normal phase flash chromatography using a 220g gold column with a gradient of 0-100% ethyl acetate in hexanes to provide 1.6g (32% yield) of **SI-3** as a yellow powder. LC-MS t1 = 3.33 min, t2 = 4.85 min, M+H = 326. ^{1}H NMR (400 MHz, DMSO-d6) δ 7.38 – 7.30 (m, 1H), 6.92 (m, 2H), 5.67 (s, 2H), 4.01 (t, J = 7.7 Hz, 2H), 2.58 (d, J = 7.1 Hz, 2H), 2.15 (m, 1H), 1.64 (m, 2H), 0.97 (d, J = 6.6 Hz, 6H), 0.91 (t, J = 7.3 Hz, 3H).

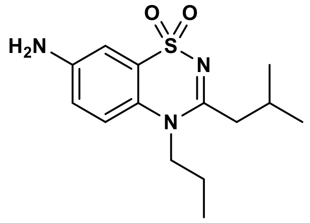

7-Amino-3-isobutyl-4-propyl-4H-benzo[e][1,2,4]thiadiazine 1,1-dioxide (SI-4):

To a solution of 3-isobutyl-7-nitro-4-propyl-4H-benzo[e][1,2,4]thiadiazine 1,1-dioxide in ethanol (100 mL) and H_2O (10 mL), iron powder (1.29 g, 23 mmol) and ammonium chloride (2.47g, 46.1 mmol) were added. The reaction was heated to 95°C for 18 hours, after which the reaction was judged complete by LC-MS. The mixture was cooled to RT, filtered through celite, and concentrated under reduced pressure to remove most of the EtOH. The residue was then diluted with water and extracted with ethyl acetate (2 x 50 mL). The organic layer was then dried over sodium sulfate and concentrated *in vacuo* to afford 1.3g of **SI-4** as a beige solid. LC-MS t1 = 2.97 min, t2 = 4.20 min, M+H = 296. ^{1}H NMR (400 MHz, DMSO-d6) δ 7.34 (m, 1H), 6.92 (m, 2H), 5.67 (s, 2H), 4.06 – 3.97 (m, 2H), 2.58 (d, J = 7.0 Hz, 2H), 2.21 – 2.09 (m, 1H), 1.64 (q, J = 7.5 Hz, 2H), 0.97 (d, J = 6.6 Hz, 6H), 0.91 (t, J = 7.3 Hz, 3H).

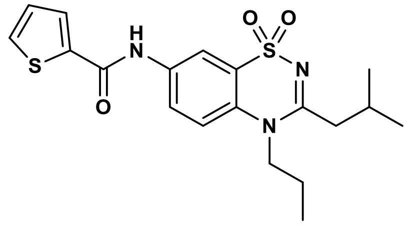

N-(3-Isobutyl-1,1-dioxido-4-propyl-4H-benzo[e][1,2,4]thiadiazin-7-yl)thiophene-2-carboxamide (1) (Initial HTS hit; NCGC00473217):

To a solution of 7-amino-3-isobutyl-4-propyl-4H-benzo[e][1,2,4]thiadiazine 1,1-dioxide (0.075g, 0.254 mmol) in dichloromethane (2 mL), triethylamine (0.042 ml, 0.305 mmol) and thiophene-2-carbonyl chloride (0.027 ml, 0.254 mmol) (*or the appropriate acid chloride*) were added. The reaction was heated to 35°C and stirred for 4 hr after which the reaction was deemed complete by LC-MS. The mixture was concentrated to ~1 mL and purified via automated normal phase flash chromatography using a 24g column and a gradient of 0-100% ethyl acetate in hexanes. The product co-elutes the starting aniline. The fractions that contained product were collected and concentrated. The resulting mixture was then dissolved in a small amount of DMSO and purified by preparative HPLC to afford **1** as a yellow solid. LC-MS t1 = 3.36 min, t2 = 5.29 min, M+H = 406. 1H NMR (400 MHz, DMSO-d6) δ 10.60 (s, 1H), 8.30 (d, J = 2.6 Hz, 1H), 8.11 (dd, J = 9.4, 2.6 Hz, 1H), 8.05 (dd, J = 3.8, 1.2 Hz, 1H), 7.91 (dd, J = 5.0, 1.1 Hz, 1H), 7.71 (d, J = 9.4 Hz, 1H), 7.26 (dd, J = 5.0, 3.8 Hz, 1H), 4.16 – 4.08 (m, 2H), 2.67 (d, J = 7.0 Hz, 2H), 2.18 (s, 1H), 1.70 (q, J = 7.5 Hz, 2H), 0.99 (d, J = 6.6 Hz, 6H), 0.94 (t, J = 7.3 Hz, 3H).

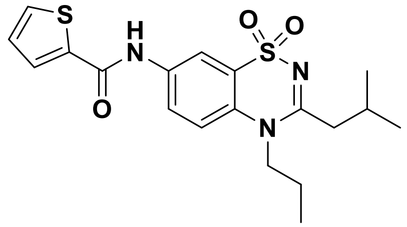

N-(3-isobutyl-1,1-dioxido-4-propyl-4H-benzo[e][1,2,4]thiadiazin-7-yl)thiophene-2-carboxamide; NCGC00473217-03:

LC-MS t2 = 5.30 min, M+H = 406. 1H NMR (400 MHz, DMSO-d6) δ 10.60 (s, 1H), 8.30 (d, J = 2.6 Hz, 1H), 8.11 (dd, J = 9.4, 2.6 Hz, 1H), 8.05 (dd, J = 3.8, 1.2 Hz, 1H), 7.91 (dd, J = 5.0, 1.1 Hz, 1H), 7.71 (d, J = 9.4 Hz, 1H), 7.26 (dd, J = 5.0, 3.8 Hz, 1H), 4.16 – 4.07 (m, 2H), 2.67 (d, J = 7.0 Hz, 2H), 2.19 (hept, J = 6.7 Hz, 1H), 1.70 (h, J = 7.4 Hz, 2H), 1.02 – 0.90 (m, 9H).

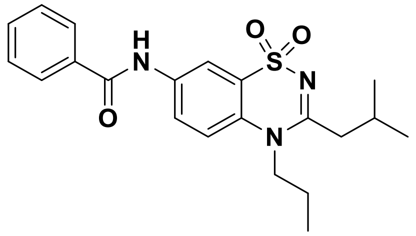

N-(3-Isobutyl-1,1-dioxido-4-propyl-4H-benzo[e][1,2,4]thiadiazin-7-yl)benzamide; NCGC00510420-01:

LC-MS t2 = 3.40 min, M+H = 400. 1H NMR (400 MHz, Chloroform-d) δ 8.53 (dd, J = 9.3, 2.6 Hz, 1H), 8.26 (s, 1H), 7.93 (dt, J = 6.9, 1.4 Hz, 2H), 7.87 (d, J = 2.6 Hz, 1H), 7.63 – 7.56 (m, 1H), 7.53 (dd, J = 8.2, 6.6 Hz, 2H), 4.02 – 3.93 (m, 2H), 2.57 (d, J = 7.0 Hz, 2H), 2.38 (dq, J = 13.5, 6.8 Hz, 1H), 1.84 (p, J = 7.5 Hz, 2H), 1.13 – 1.03 (m, 9H).

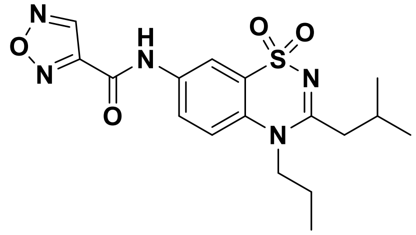

N-(3-Isobutyl-1,1-dioxido-4-propyl-4H-benzo[e][1,2,4]thiadiazin-7-yl)-1,2,5-oxadiazole-3-carboxamide NCGC00521034-01:

LC-MS t2 = 4.99 min, M+H = 392. 1H NMR (400 MHz, DMSO-d6) δ 11.46 (s, 1H), 9.45 (s, 1H), 8.39 (d, J = 2.5 Hz, 1H), 8.08 (dd, J = 9.3, 2.6 Hz, 1H), 7.76 (d, J = 9.4 Hz, 1H), 4.12 (t, J = 7.7 Hz, 2H), 2.68 (d, J = 7.1 Hz, 2H), 2.20 (m, J = 6.7 Hz, 1H), 1.70 (h, J = 7.2 Hz, 2H), 1.03 – 0.90 (m, 9H).

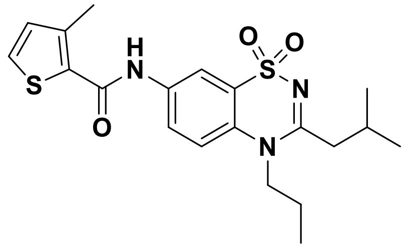

N-(3-Isobutyl-1,1-dioxido-4-propyl-4H-benzo[e][1,2,4]thiadiazin-7-yl)-3-methylthiophene -2-carboxamide; NCGC00521035-01:

LC-MS t2 = 5.40 min, M+H = 420. 1H NMR (400 MHz, DMSO-d6) δ 10.34 (s, 1H), 8.31 (d, J = 2.5 Hz, 1H), 8.00 (dd, J = 9.3, 2.6 Hz, 1H), 7.74 – 7.65 (m, 2H), 7.05 (d, J = 5.0 Hz, 1H), 4.11 (t, J = 7.7 Hz, 2H), 2.67 (d, J = 6.9 Hz, 2H), 2.47 (s, 3H), 2.19 (hept, J = 6.8 Hz, 1H), 1.69 (h, J = 7.4 Hz, 2H), 0.99 (d, J = 6.6 Hz, 6H), 0.94 (t, J = 7.3 Hz, 3H).

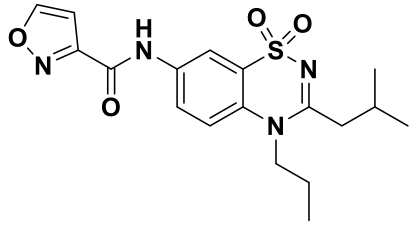

N-(3-Isobutyl-1,1-dioxido-4-propyl-4H-benzo[e][1,2,4]thiadiazin-7-yl)isoxazole-3-carboxamide; NCGC00521044-01:

LC-MS t2 = 4.87 min, M+H = 391. 1H NMR (400 MHz, DMSO-d6) δ 11.15 (s, 1H), 9.19 (d, J = 1.7 Hz, 1H), 8.43 (d, J = 2.5 Hz, 1H), 8.10 (dd, J = 9.3, 2.6 Hz, 1H), 7.73 (d, J = 9.4 Hz, 1H), 7.06 (d, J = 1.7 Hz, 1H), 4.11 (t, J = 7.7 Hz, 2H), 2.68 (d, J = 7.0 Hz, 2H), 2.19 (hept, J = 6.7 Hz, 1H), 1.69 (h, J = 7.4 Hz, 2H), 1.02 – 0.90 (m, 9H).

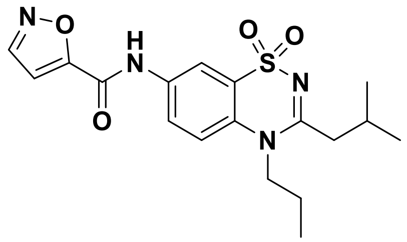

N-(3-Isobutyl-1,1-dioxido-4-propyl-4H-benzo[e][1,2,4]thiadiazin-7-yl)isoxazole-5-carboxamide; NCGC00521048-01:

LC-MS t2 = 4.81 min, M+H = 391. 1H NMR (400 MHz, DMSO-d6) δ 11.13 (s, 1H), 8.85 (d, J = 1.9 Hz, 1H), 8.37 (d, J = 2.5 Hz, 1H), 8.10 (dd, J = 9.4, 2.6 Hz, 1H), 7.74 (d, J = 9.4 Hz, 1H), 7.31 (d, J = 1.9 Hz, 1H), 4.12 (t, J = 7.7 Hz, 2H), 2.68 (d, J = 7.0 Hz, 2H), 2.19 (hept, J = 6.7 Hz, 1H), 1.70 (h, J = 7.4 Hz, 2H), 1.02 – 0.90 (m, 9H).

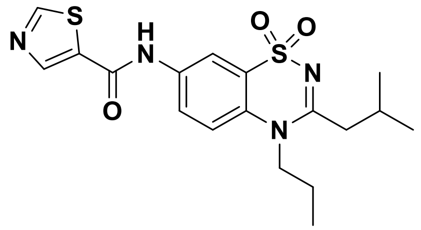

N-(3-isobutyl-1,1-dioxido-4-propyl-4H-benzo[e][1,2,4]thiadiazin-7-yl)thiazole-5-carbo-xamide; NCGC00521052-01:

LC-MS t2 = 4.73 min, M+H = 407. 1H NMR (400 MHz, DMSO-d6) δ 10.81 (s, 1H), 9.34 (s, 1H), 8.72 (s, 1H), 8.28 (d, J = 2.5 Hz, 1H), 8.08 (dd, J = 9.3, 2.6 Hz, 1H), 7.73 (d, J = 9.4 Hz, 1H), 4.12 (t, J = 7.7 Hz, 2H), 2.68 (d, J = 7.0 Hz, 2H), 2.20 (hept, J = 6.7 Hz, 1H), 1.70 (h, J = 7.4 Hz, 2H), 0.99 (d, J = 6.6 Hz, 6H), 0.95 (t, J = 7.3 Hz, 3H).

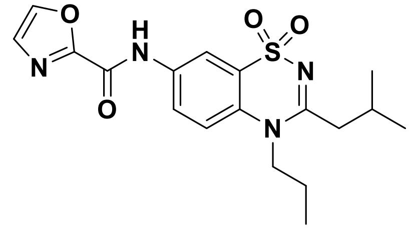

N-(3-Isobutyl-1,1-dioxido-4-propyl-4H-benzo[e][1,2,4]thiadiazin-7-yl)oxazole-2-carbo-xamide; NCGC00521053-01:

LC-MS t2 = 4.69 min, M+H = 391. 1H NMR (400 MHz, DMSO-d6) δ 11.29 (s, 1H), 8.49 – 8.42 (m, 2H), 8.13 (dd, J = 9.3, 2.6 Hz, 1H), 7.72 (d, J = 9.4 Hz, 1H), 7.58 (s, 1H), 4.11 (q, J = 6.4, 5.2 Hz, 2H), 2.67 (d, J = 7.0 Hz, 2H), 2.19 (hept, J = 6.8 Hz, 1H), 1.69 (h, J = 7.4 Hz, 2H), 1.02 – 0.90 (m, 9H).

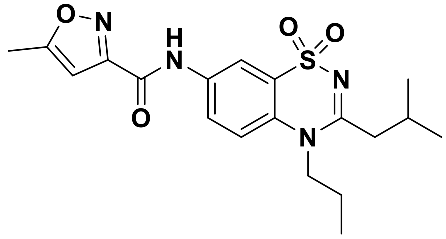

N-(3-Isobutyl-1,1-dioxido-4-propyl-4H-benzo[e][1,2,4]thiadiazin-7-yl)-5-methylisoxazole-3-carboxamide; NCGC00521054-01:

LC-MS t2 = 5.12 min, M+H = 405. 1H NMR (400 MHz, DMSO-d6) δ 11.06 (s, 1H), 8.43 (d, J = 2.5 Hz, 1H), 8.09 (dd, J = 9.4, 2.6 Hz, 1H), 7.72 (d, J = 9.4 Hz, 1H), 6.71 (d, J = 1.1 Hz, 1H),

4.11 (t, J = 7.7 Hz, 2H), 2.67 (d, J = 6.9 Hz, 2H), 2.52 (s, 2H), 2.19 (hept, J = 6.8 Hz, 1H), 1.69 (h, J = 7.4 Hz, 2H), 1.02 – 0.90 (m, 9H).

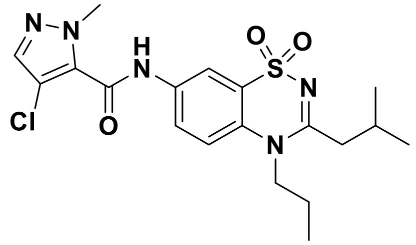

4-Chloro-N-(3-isobutyl-1,1-dioxido-4-propyl-4H-benzo[e][1,2,4]thiadiazin-7-yl)-1-methyl-1H-pyrazole-5-carboxamide; NCGC00521056-01:

LC-MS t2 = 5.20 min, M+H = 439. 1H NMR (400 MHz, DMSO-d6) δ 10.95 (s, 1H), 8.33 (d, J = 2.5 Hz, 1H), 7.96 (dd, J = 9.4, 2.6 Hz, 1H), 7.77 – 7.69 (m, 2H), 4.12 (t, J = 7.6 Hz, 2H), 3.96 (s, 3H), 2.68 (d, J = 7.2 Hz, 2H), 2.19 (dp, J = 13.4, 6.7 Hz, 1H), 1.70 (p, J = 7.5 Hz, 2H), 0.99 (d, J = 6.6 Hz, 6H), 0.94 (t, J = 7.3 Hz, 3H).

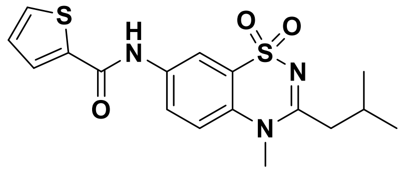

N-(3-Isobutyl-4-methyl-1,1-dioxido-4H-benzo[e][1,2,4]thiadiazin-7-yl)thiophene-2-carboxamide; NCGC00521057-01:

LC-MS t2 = 4.61 min, M+H = 378.

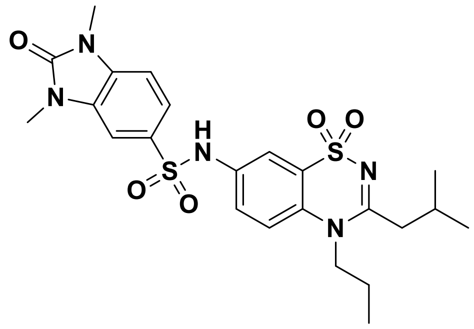

N-(3-Isobutyl-1,1-dioxido-4-propyl-4H-benzo[e][1,2,4]thiadiazin-7-yl)-1,3-dimethyl-2-oxo-2,3-dihydro-1H-benzo[d]imidazole-5-sulfonamide; NCGC00521090-01:

LC-MS t2 = 4.77 min, M+H = 520. 1H NMR (400 MHz, DMSO-d6) δ 10.67 (s, 1H), 7.60 – 7.43 (m, 6H), 7.30 (d, J = 8.2 Hz, 1H), 4.01 (t, J = 7.7 Hz, 2H), 3.33 (d, J = 4.1 Hz, 6H), 2.61 (d, J = 7.0 Hz, 2H), 2.13 (dq, J = 13.5, 6.8 Hz, 1H), 1.60 (q, J = 7.5 Hz, 2H), 0.95 (d, J = 6.6 Hz, 6H), 0.88 (t, J = 7.3 Hz, 3H).

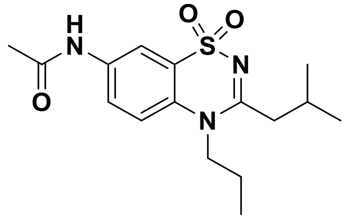

N-(3-Isobutyl-1,1-dioxido-4-propyl-4H-benzo[e][1,2,4]thiadiazin-7-yl)acetamide; NCGC00521093-01:

LC-MS t2 = 4.40 min, M+H = 338. 1H NMR (400 MHz, DMSO-d6) δ 10.34 (s, 1H), 8.22 (d, J = 2.5 Hz, 1H), 7.79 (dd, J = 9.3, 2.5 Hz, 1H), 7.64 (d, J = 9.3 Hz, 1H), 4.08 (t, J = 7.7 Hz, 2H), 2.65 (d, J = 7.1 Hz, 2H), 2.18 (hept, J = 6.8 Hz, 1H), 2.08 (s, 3H), 1.67 (h, J = 7.4 Hz, 2H), 1.01 – 0.89 (m, 9H).

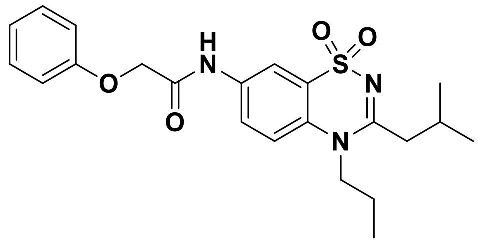

N-(3-Isobutyl-1,1-dioxido-4-propyl-4H-benzo[e][1,2,4]thiadiazin-7-yl)-2-phenoxyacetamide; NCGC00521094-01:

LC-MS t2 = 5.46 min, M+H = 430. 1H NMR (400 MHz, DMSO-d6) δ 10.49 (s, 1H), 8.27 (d, J = 2.5 Hz, 1H), 7.96 (dd, J = 9.3, 2.5 Hz, 1H), 7.68 (d, J = 9.4 Hz, 1H), 7.37 – 7.28 (m, 2H), 7.03 (d, J = 1.2 Hz, 1H), 7.04 – 6.94 (m, 2H), 4.74 (s, 2H), 4.10 (t, J = 7.7 Hz, 2H), 2.66 (d, J = 6.9 Hz, 2H), 2.18 (hept, J = 6.7 Hz, 1H), 1.68 (h, J = 7.4 Hz, 2H), 0.98 (d, J = 6.6 Hz, 6H), 0.93 (t, J = 7.3 Hz, 3H).

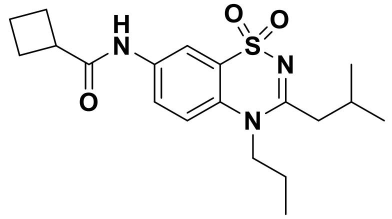

N-(3-Isobutyl-1,1-dioxido-4-propyl-4H-benzo[e][1,2,4]thiadiazin-7-yl)cyclobutenecarbo-xamide; NCGC00521095-01:

LC-MS t2 = 5.06 min, M+H = 378. 1H NMR (400 MHz, DMSO-d6) δ 10.12 (s, 1H), 8.25 (d, J = 2.5 Hz, 1H), 7.84 (dd, J = 9.3, 2.5 Hz, 1H), 7.63 (d, J = 9.4 Hz, 1H), 4.08 (t, J = 7.7 Hz, 2H), 3.22 (q, J = 8.4 Hz, 1H), 2.65 (d, J = 7.0 Hz, 2H), 2.30 – 2.07 (m, 5H), 1.96 (dq, J = 10.7, 8.5 Hz, 1H), 1.88 – 1.77 (m, 1H), 1.68 (h, J = 7.3 Hz, 2H), 1.03 – 0.94 (m, 6H), 0.92 (d, J = 7.4 Hz, 3H).

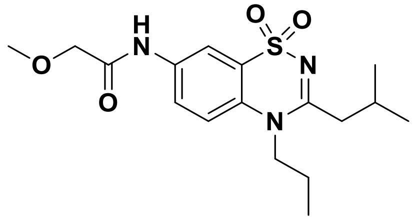

N-(3-Isobutyl-1,1-dioxido-4-propyl-4H-benzo[e][1,2,4]thiadiazin-7-yl)-2-methoxyaceta-mide NCGC00521098-01:

LC-MS t2 = 4.57 min, M+H = 368. 1H NMR (400 MHz, DMSO-d6) δ 10.21 (s, 1H), 8.33 (d, J = 2.5 Hz, 1H), 7.96 (dd, J = 9.3, 2.5 Hz, 1H), 7.66 (d, J = 9.4 Hz, 1H), 4.09 (t, J = 7.7 Hz, 2H), 4.04 (s, 2H), 3.39 (s, 2H), 2.66 (d, J = 7.1 Hz, 2H), 2.18 (hept, J = 6.7 Hz, 1H), 1.67 (h, J = 7.4 Hz, 2H), 0.98 (d, J = 6.6 Hz, 6H), 0.93 (t, J = 7.3 Hz, 3H).

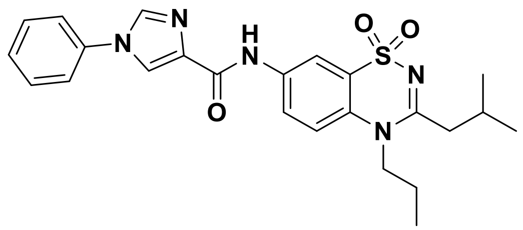

Synthesis of N-(3-isobutyl-1,1-dioxido-4-propyl-4H-benzo[e][1,2,4]thiadiazin-7-yl)-1-phenyl-1H-imidazole-4-carboxamide NCGC00521102-01:

LC-MS t2 = 5.31 min, M+H = 466.

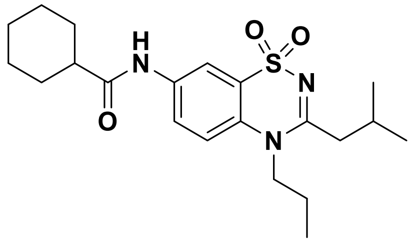

N-(3-Isobutyl-1,1-dioxido-4-propyl-4H-benzo[e][1,2,4]thiadiazin-7-yl)cyclohexanecarboxamide; NCGC00521107-01:

LC-MS t2 = 5.55 min, M+H = 406. 1H NMR (400 MHz, DMSO-d6) δ 10.21 (s, 1H), 8.26 (d, J = 2.4 Hz, 1H), 7.82 (dd, J = 9.3, 2.5 Hz, 1H), 7.63 (d, J = 9.4 Hz, 1H), 4.08 (t, J = 7.7 Hz, 2H), 2.65 (d, J = 7.0 Hz, 2H), 2.18 (hept, J = 6.7 Hz, 1H), 1.80 (t, J = 12.2 Hz, 3H), 1.75 (s, 1H), 1.67 (q, J = 7.9 Hz, 3H), 1.45 (d, J = 12.1 Hz, 1H), 1.39 (d, J = 11.7 Hz, 1H), 1.23 (tt, J = 24.3, 12.0 Hz, 3H), 1.01 – 0.88 (m, 9H).

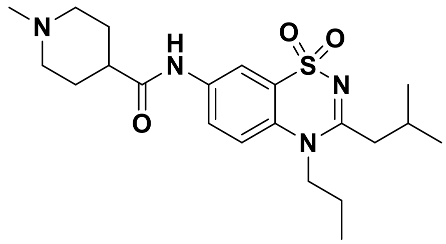

N-(3-Isobutyl-1,1-dioxido-4-propyl-4H-benzo[e][1,2,4]thiadiazin-7-yl)-1-methylpiperidine-4-carboxamide NCGC00521111-01:

LC-MS t2 = 3.81 min, M+H = 421. 1H NMR (400 MHz, DMSO-d6) δ 10.47 (s, 1H), 9.25 (s, 1H), 8.28 (s, 1H), 7.79 (dd, J = 9.3, 2.6 Hz, 1H), 7.66 (d, J = 9.4 Hz, 1H), 4.09 (t, J = 7.7 Hz, 2H), 3.49 (d, J = 12.0 Hz, 2H), 2.97 (s, 2H), 2.79 (s, 3H), 2.66 (d, J = 7.1 Hz, 2H), 2.58 (s, 1H), 2.18 (dt, J = 13.4, 6.7 Hz, 1H), 2.05 (d, J = 13.9 Hz, 2H), 1.85 (t, J = 13.3 Hz, 2H), 1.66 (p, J = 7.4 Hz, 2H), 0.98 (d, J = 6.6 Hz, 6H), 0.93 (t, J = 7.3 Hz, 3H).

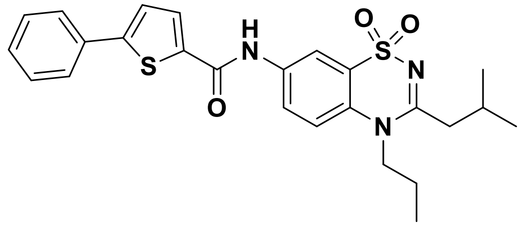

N-(3-Isobutyl-1,1-dioxido-4-propyl-4H-benzo[e][1,2,4]thiadiazin-7-yl)-5-phenylthiophene-2-carboxamide; NCGC00521113-01:

LC-MS t2 = 6.16 min, M+H = 482.

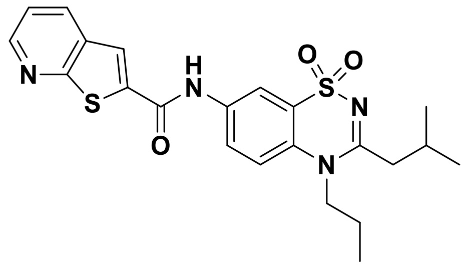

N-(3-isobutyl-1,1-dioxido-4-propyl-4H-benzo[e][1,2,4]thiadiazin-7-yl)thieno[2,3-b]pyridine-2-carboxamide; NCGC00521132-01:

LC-MS t2 = 5.26 min, M+H = 457. 1H NMR (400 MHz, DMSO-d6) δ 10.98 (s, 1H), 8.71 (dd, J = 4.6, 1.7 Hz, 1H), 8.48 (dd, J = 8.2, 1.7 Hz, 1H), 8.39 (s, 1H), 8.34 (d, J = 2.5 Hz, 1H), 8.16 (dd, J = 9.3, 2.6 Hz, 1H), 7.75 (d, J = 9.4 Hz, 1H), 7.55 (dd, J = 8.1, 4.6 Hz, 1H), 4.17 – 4.04 (m, 2H), 3.17 (d, J = 5.3 Hz, 1H), 2.68 (d, J = 7.1 Hz, 2H), 2.20 (dt, J = 13.4, 6.7 Hz, 1H), 1.71 (q, J = 7.6 Hz, 2H), 1.00 (d, J = 6.6 Hz, 6H), 0.95 (t, J = 7.3 Hz, 3H).

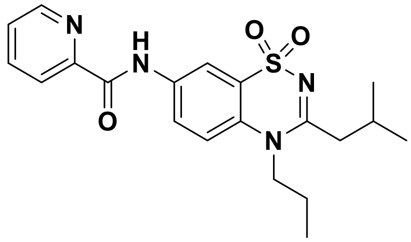

N-(3-Isobutyl-1,1-dioxido-4-propyl-4H-benzo[e][1,2,4]thiadiazin-7-yl)picolinamide; NCGC00521133-01:

LC-MS t2 = 5.20 min, 401. 1H NMR (400 MHz, DMSO-d6) δ 11.14 (s, 1H), 8.77 (dt, J = 4.5, 1.4 Hz, 1H), 8.64 (d, J = 2.5 Hz, 1H), 8.25 – 8.15 (m, 2H), 8.09 (td, J = 7.7, 1.7 Hz, 1H), 7.75 – 7.67 (m, 2H), 4.12 (t, J = 7.8 Hz, 2H), 2.68 (d, J = 6.9 Hz, 2H), 2.20 (hept, J = 6.6 Hz, 1H), 1.69 (p, J = 7.4 Hz, 2H), 1.00 (d, J = 6.6 Hz, 6H), 0.95 (t, J = 7.3 Hz, 3H).

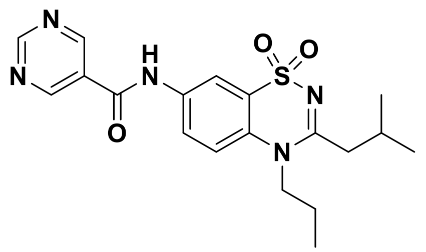

N-(3-Isobutyl-1,1-dioxido-4-propyl-4H-benzo[e][1,2,4]thiadiazin-7-yl)pyrimidine-5-carboxamide; NCGC00521134-01:

LC-MS t2 = 4.56 min, M+H = 402. 1H NMR (400 MHz, DMSO-d6) δ 10.95 (s, 1H), 9.39 (s, 1H), 9.31 (s, 2H), 8.37 (d, J = 2.5 Hz, 1H), 8.08 (dd, J = 9.3, 2.5 Hz, 1H), 7.75 (d, J = 9.4 Hz, 1H), 4.12 (t, J = 7.7 Hz, 2H), 2.68 (d, J = 7.1 Hz, 2H), 2.20 (hept, J = 6.7 Hz, 1H), 1.70 (h, J = 7.3 Hz, 2H), 1.00 (d, J = 6.6 Hz, 6H), 0.95 (t, J = 7.3 Hz, 3H).

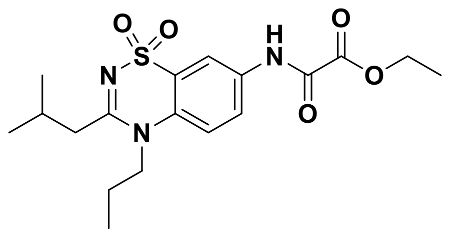

Ethyl 2-((3-isobutyl-1,1-dioxido-4-propyl-4H-benzo[e][1,2,4]thiadiazin-7-yl)amino)-2-oxoacetate; NCGC00521135-01:

LC-MS t2 = 4.90 min, M+H = 396. 1H NMR (400 MHz, DMSO-d6) δ 11.18 (s, 1H), 8.37 (d, J = 2.5 Hz, 1H), 8.08 (dd, J = 9.3, 2.6 Hz, 1H), 7.72 (d, J = 9.4 Hz, 1H), 4.33 (q, J = 7.1 Hz, 2H), 4.10 (t, J = 7.7 Hz, 2H), 2.67 (d, J = 7.0 Hz, 2H), 2.19 (hept, J = 6.7 Hz, 1H), 1.68 (h, J = 7.4 Hz, 2H), 1.33 (t, J = 7.1 Hz, 3H), 0.99 (d, J = 6.6 Hz, 6H), 0.93 (t, J = 7.3 Hz, 3H).

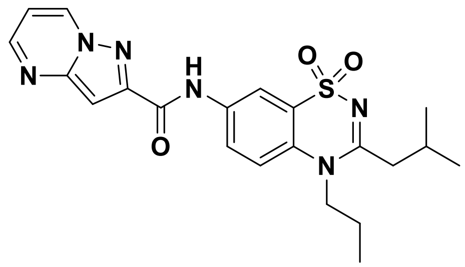

N-(3-Isobutyl-1,1-dioxido-4-propyl-4H-benzo[e][1,2,4]thiadiazin-7-yl)pyrazolo[1,5-a]pyrimidine-3-carboxamide; NCGC00521136-01:

LC-MS t2 = 4.91 min, M+H = 441. 1H NMR (400 MHz, DMSO-d6) δ 10.27 (s, 1H), 9.38 (dd, J = 7.0, 1.7 Hz, 1H), 8.93 (dd, J = 4.2, 1.7 Hz, 1H), 8.77 (s, 1H), 8.34 (d, J = 2.5 Hz, 1H), 8.05 (dd, J = 9.3, 2.6 Hz, 1H), 7.72 (d, J = 9.3 Hz, 1H), 7.36 (dd, J = 7.0, 4.2 Hz, 1H), 4.13 (t, J = 7.7 Hz, 2H), 2.68 (d, J = 7.0 Hz, 2H), 2.20 (hept, J = 6.7 Hz, 1H), 1.71 (h, J = 7.4 Hz, 2H), 1.00 (d, J = 6.6 Hz, 6H), 0.95 (t, J = 7.3 Hz, 3H).

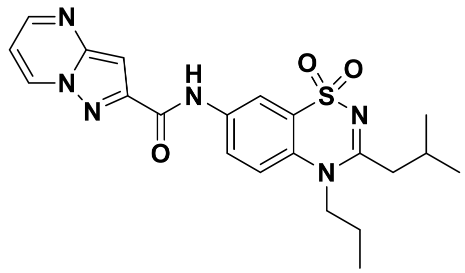

N-(3-Isobutyl-1,1-dioxido-4-propyl-4H-benzo[e][1,2,4]thiadiazin-7-yl)pyrazolo[1,5-a]pyrimidine-2-carboxamide; NCGC00521137-01:

LC-MS t2 = 4.79 min, M+H = 441. 1H NMR (400 MHz, DMSO-d6) δ 10.98 (s, 1H), 9.18 (dt, J = 7.0, 1.4 Hz, 1H), 8.70 (dd, J = 4.0, 1.7 Hz, 1H), 8.53 (d, J = 2.5 Hz, 1H), 8.19 (dd, J = 9.4, 2.5 Hz, 1H), 7.73 (d, J = 9.4 Hz, 1H), 7.30 – 7.22 (m, 2H), 4.12 (t, J = 7.8 Hz, 2H), 2.68 (d, J = 7.0 Hz, 2H), 2.20 (hept, J = 6.7 Hz, 1H), 1.70 (h, J = 7.5 Hz, 2H), 1.03 – 0.91 (m, 9H).

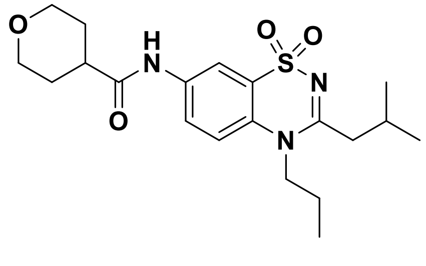

N-(3-isobutyl-1,1-dioxido-4-propyl-4H-benzo[e][1,2,4]thiadiazin-7-yl)tetrahydro-2H-pyran-4-carboxamide; NCGC00521138-01:

LC-MS t2 = 4.61 min, M+H = 408. 1H NMR (400 MHz, DMSO-d6) δ 10.29 (s, 1H), 8.25 (d, J = 2.5 Hz, 1H), 7.84 (dd, J = 9.3, 2.5 Hz, 1H), 7.64 (d, J = 9.4 Hz, 1H), 4.08 (t, J = 7.7 Hz, 2H), 3.95 – 3.87 (m, 2H), 3.36 (td, J = 11.3, 2.9 Hz, 2H), 2.65 (d, J = 7.1 Hz, 2H), 2.59 (dq, J = 10.6, 5.4, 4.4 Hz, 1H), 2.18 (hept, J = 6.6 Hz, 1H), 1.69 (dq, J = 14.2, 9.1, 6.5 Hz, 6H), 0.98 (d, J = 6.6 Hz, 6H), 0.92 (t, J = 7.3 Hz, 3H).

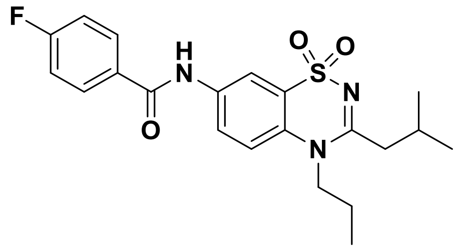

4-Fluoro-N-(3-isobutyl-1,1-dioxido-4-propyl-4H-benzo[e][1,2,4]thiadiazin-7-yl)benzamide; NCGC00521139-01:

LC-MS t2 = 5.36 min, M+H = 4.18.

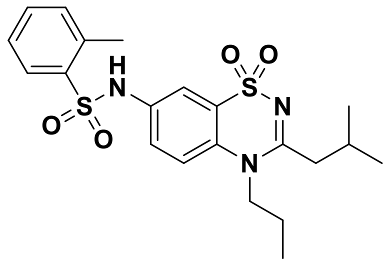

N-(3-Isobutyl-1,1-dioxido-4-propyl-4H-benzo[e][1,2,4]thiadiazin-7-yl)-2-methyl-benzenesulfonamide; NCGC00521140-01:

LC-MS t2 = 5.39 min, M+H = 450. 1H NMR (400 MHz, DMSO-d6) δ 10.76 (s, 1H), 7.63 – 7.54 (m, 3H), 7.50 – 7.42 (m, 4H), 4.02 (t, J = 7.8 Hz, 2H), 2.62 (d, J = 7.0 Hz, 2H), 2.34 (s, 3H), 2.14 (dq, J = 13.3, 6.8 Hz, 1H), 1.62 (h, J = 7.5 Hz, 2H), 0.96 (d, J = 6.6 Hz, 6H), 0.89 (t, J = 7.3 Hz, 3H).

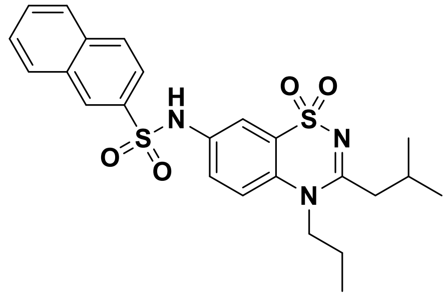

N-(3-Isobutyl-1,1-dioxido-4-propyl-4H-benzo[e][1,2,4]thiadiazin-7-yl)naphthalene-2-sulfonamide; NCGC00521141-01:

LC-MS t2 = 5.66 min, M+H = 486.

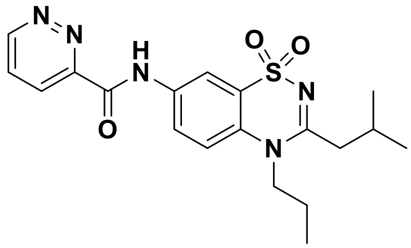

N-(3-Isobutyl-1,1-dioxido-4-propyl-4H-benzo[e][1,2,4]thiadiazin-7-yl)pyridazine-3-carboxamide; NCGC00521144-01:

LC-MS t2 = 4.78 min, M+H = 402. 1H NMR (400 MHz, DMSO-d6) δ 11.56 (s, 1H), 9.50 (dd, J = 5.0, 1.7 Hz, 1H), 8.65 (d, J = 2.5 Hz, 1H), 8.35 (dd, J = 8.5, 1.7 Hz, 1H), 8.25 (dd, J = 9.3, 2.6 Hz, 1H), 8.00 (dd, J = 8.5, 5.0 Hz, 1H), 7.74 (d, J = 9.4 Hz, 1H), 4.13 (t, J = 7.7 Hz, 2H), 2.68 (d, J = 7.2 Hz, 2H), 2.20 (dt, J = 13.5, 6.7 Hz, 1H), 1.70 (q, J = 7.5 Hz, 2H), 1.00 (d, J = 6.6 Hz, 6H), 0.95 (t, J = 7.3 Hz, 3H).

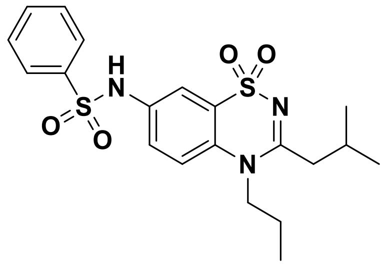

N-(3-Isobutyl-1,1-dioxido-4-propyl-4H-benzo[e][1,2,4]thiadiazin-7-yl)benzene-sulfonamide; NCGC00521146-01:

LC-MS t2 = 5.17 min, M+H = 436. 1H NMR (400 MHz, DMSO-d6) δ 10.82 (s, 1H), 7.79 (dt, J = 7.1, 1.5 Hz, 2H), 7.69 – 7.60 (m, 1H), 7.64 – 7.54 (m, 3H), 7.47 (h, J = 2.6 Hz, 2H), 4.01 (t, J = 7.8 Hz, 2H), 2.61 (d, J = 7.0 Hz, 2H), 2.14 (hept, J = 6.8 Hz, 1H), 1.61 (h, J = 7.3 Hz, 2H), 0.96 (d, J = 6.6 Hz, 6H), 0.89 (t, J = 7.3 Hz, 3H).

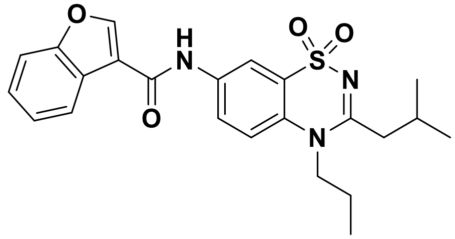

N-(3-Isobutyl-1,1-dioxido-4-propyl-4H-benzo[e][1,2,4]thiadiazin-7-yl)benzofuran-3-carboxamide; NCGC00521147-01:

LC-MS t2 = 5.68 min, M+H = 440.

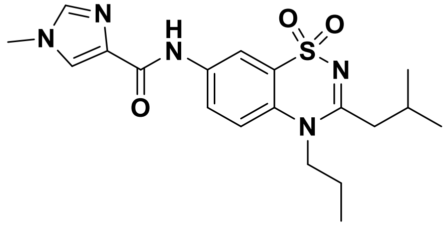

N-(3-Isobutyl-1,1-dioxido-4-propyl-4H-benzo[e][1,2,4]thiadiazin-7-yl)-1-methyl-1H-imidazole-4-carboxamide; NCGC00521148-01:

LC-MS t2 = 4.17 min, M+H = 404.

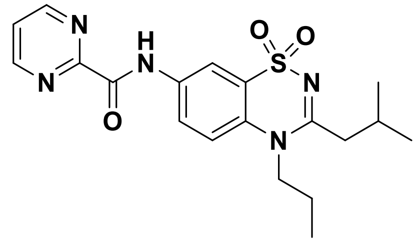

N-(3-Isobutyl-1,1-dioxido-4-propyl-4H-benzo[e][1,2,4]thiadiazin-7-yl)pyrimidine-2-carboxamide; NCGC00521149-01:

LC-MS t2 = 4.55 min, M+H = 402. 1H NMR (400 MHz, DMSO-d6) δ 11.24 (s, 1H), 9.07 (d, J = 4.9 Hz, 2H), 8.58 (d, J = 2.5 Hz, 1H), 8.19 (dd, J = 9.3, 2.5 Hz, 1H), 7.81 – 7.69 (m, 2H), 4.12 (t, J = 7.7 Hz, 2H), 2.68 (d, J = 7.1 Hz, 2H), 2.20 (hept, J = 6.6 Hz, 1H), 1.71 (p, J = 7.5 Hz, 2H), 1.00 (d, J = 6.6 Hz, 6H), 0.95 (t, J = 7.3 Hz, 3H).

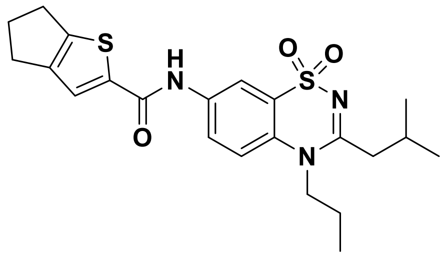

N-(3-Isobutyl-1,1-dioxido-4-propyl-4H-benzo[e][1,2,4]thiadiazin-7-yl)-5,6-dihydro-4H-cyclopenta[b]thiophene-2-carboxamide NCGC00521150-01:

LC-MS t2 = 4.85 min, M+H = 446.

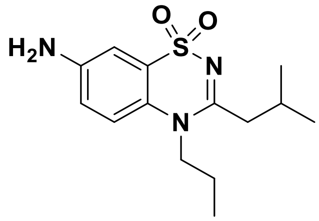

7-Amino-3-isobutyl-4-propyl-4H-benzo[e][1,2,4]thiadiazine 1,1-dioxide NCGC00521151-01:

LC-MS t2 = 4.21 min, M+H = 296. 1H NMR (400 MHz, DMSO-d6) δ 7.38 – 7.31 (m, 1H), 6.92 (dq, J = 4.8, 2.7 Hz, 2H), 5.67 (s, 2H), 4.01 (t, J = 7.7 Hz, 2H), 2.58 (d, J = 7.0 Hz, 2H), 2.15 (hept, J = 6.8 Hz, 1H), 1.64 (h, J = 7.5 Hz, 2H), 1.02 – 0.87 (m, 9H).

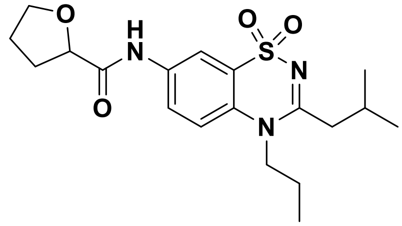

N-(3-Isobutyl-1,1-dioxido-4-propyl-4H-benzo[e][1,2,4]thiadiazin-7-yl)tetrahydrofuran-2-carboxamide; NCGC00521155-01:

LC-MS t2 = 4.78 min, M+H = 394.

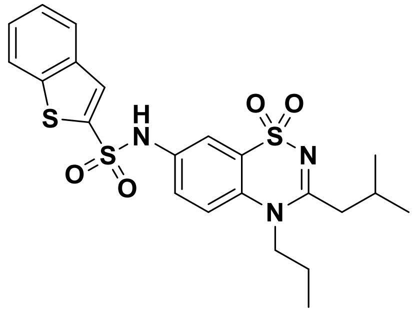

N-(3-Isobutyl-1,1-dioxido-4-propyl-4H-benzo[e][1,2,4]thiadiazin-7-yl)benzo[b]thiophene-2-sulfonamide; NCGC00521156-01:

LC-MS t2 = 5.65 min, M+H = 492. 1H NMR (400 MHz, DMSO-d6) δ 11.21 (s, 1H), 8.10 – 7.97 (m, 3H), 7.65 – 7.44 (m, 5H), 4.01 (t, J = 7.8 Hz, 2H), 2.61 (d, J = 7.0 Hz, 2H), 2.14 (hept, J = 6.8 Hz, 1H), 1.69 – 1.55 (m, 2H), 0.95 (d, J = 6.6 Hz, 6H), 0.89 (t, J = 7.3 Hz, 3H).

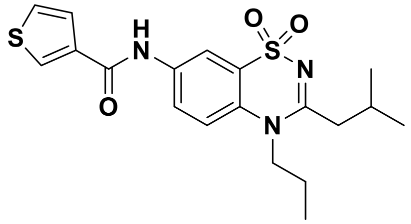

N-(3-Isobutyl-1,1-dioxido-4-propyl-4H-benzo[e][1,2,4]thiadiazin-7-yl)thiophene-3-carboxamide; NCGC00521157-01:

LC-MS t2 = 5.13 min, M+H = 406.

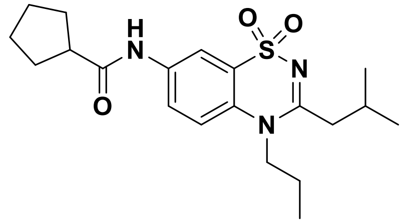

N-(3-Isobutyl-1,1-dioxido-4-propyl-4H-benzo[e][1,2,4]thiadiazin-7-yl)cyclopentane-carboxamide; NCGC00521158-01:

LC-MS t2 = 5.32 min, M+H = 392.

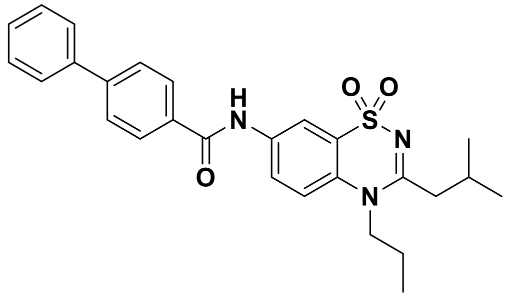

N-(3-isobutyl-1,1-dioxido-4-propyl-4H-benzo[e][1,2,4]thiadiazin-7-yl)-[1,1'-biphenyl]-4-carboxamide; NCGC00521159-01:

LC-MS t2 = 6.13 min, M+H = 476.

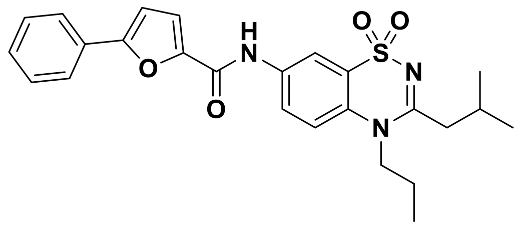

N-(3-isobutyl-1,1-dioxido-4-propyl-4H-benzo[e][1,2,4]thiadiazin-7-yl)-5-phenylfuran-2-carboxamide; NCGC00521160-01:

LC-MS t2 = 5.93 min, M+H = 466. 1H NMR (400 MHz, DMSO-d6) δ 10.53 (s, 1H), 8.34 (d, J = 2.5 Hz, 1H), 8.19 (dd, J = 9.4, 2.6 Hz, 1H), 8.02 – 7.95 (m, 2H), 7.73 (d, J = 9.4 Hz, 1H), 7.52 (td, J = 7.3, 6.5, 1.2 Hz, 2H), 7.47 (d, J = 3.7 Hz, 1H), 7.45 – 7.37 (m, 1H), 7.21 (d, J = 3.7 Hz, 1H), 4.13 (t, J = 7.7 Hz, 2H), 2.68 (d, J = 7.1 Hz, 2H), 2.20 (hept, J = 6.7 Hz, 1H), 1.78 – 1.64 (m, 2H), 1.00 (d, J = 6.6 Hz, 6H), 0.95 (t, J = 7.3 Hz, 3H).

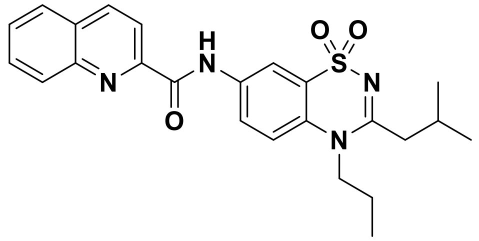

N-(3-isobutyl-1,1-dioxido-4-propyl-4H-benzo[e][1,2,4]thiadiazin-7-yl)quinoline-2-carboxamide; NCGC00521161-01:

LC-MS t2 = 6.12 min, M+H = 451. 1H NMR (400 MHz, DMSO-d6) δ 11.22 (s, 1H), 8.69 – 8.62 (m, 2H), 8.32 (dd, J = 9.3, 2.5 Hz, 1H), 8.27 (dd, J = 8.6, 6.6 Hz, 2H), 8.14 (dd, J = 8.1, 1.5 Hz, 1H), 7.94 (ddd, J = 8.5, 6.9, 1.5 Hz, 1H), 7.82 – 7.72 (m, 2H), 4.14 (t, J = 7.6 Hz, 2H), 2.69 (d, J = 7.0 Hz, 2H), 2.21 (hept, J = 6.8 Hz, 1H), 1.71 (p, J = 7.4 Hz, 2H), 1.01 (d, J = 6.6 Hz, 6H), 0.96 (t, J = 7.3 Hz, 3H).

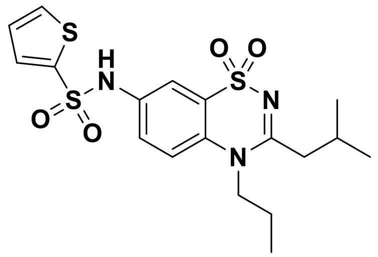

N-(3-isobutyl-1,1-dioxido-4-propyl-4H-benzo[e][1,2,4]thiadiazin-7-yl)thiophene-2-sulfonamide; NCGC00521163-01:

LC-MS t2 = 5.12 min, M+H = 442.

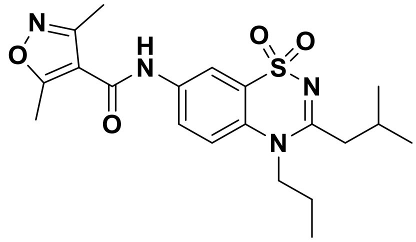

N-(3-isobutyl-1,1-dioxido-4-propyl-4H-benzo[e][1,2,4]thiadiazin-7-yl)-3,5-dimethylisoxazole-4-carboxamide; NCGC00521866-01:

LC-MS t2 = 4.98 min, M+H = 419.

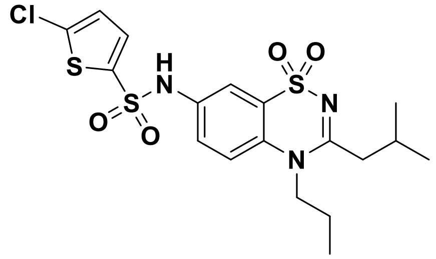

5-chloro-N-(3-isobutyl-1,1-dioxido-4-propyl-4H-benzo[e][1,2,4]thiadiazin-7-yl)thiophene-2-sulfonamide; NCGC00521874-01:

LC-MS t2 = 5.58 min, M+H = 477. 1H NMR (400 MHz, DMSO-d6) δ 11.09 (s, 1H), 7.66 (d, J = 9.2 Hz, 1H), 7.55 – 7.45 (m, 3H), 7.24 (dt, J = 4.1, 1.2 Hz, 1H), 4.05 (t, J = 7.8 Hz, 2H), 2.64 (d, J = 7.0 Hz, 2H), 2.18 (dq, J = 13.5, 6.7 Hz, 1H), 1.65 (q, J = 7.6 Hz, 2H), 0.97 (d, J = 6.6 Hz, 6H), 0.92 (t, J = 7.3 Hz, 3H).

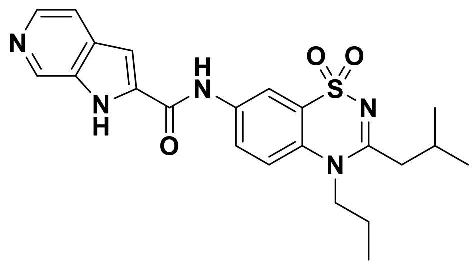

N-(3-isobutyl-1,1-dioxido-4-propyl-4H-benzo[e][1,2,4]thiadiazin-7-yl)-1H-pyrrolo[2,3-c]pyridine-2-carboxamide; NCGC00521877-01:

LC-MS t2 = 4.16 min, M+H = 440

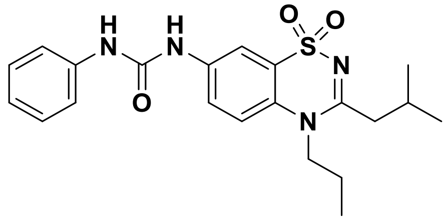

1-(3-Isobutyl-1,1-dioxido-4-propyl-4H-benzo[e][1,2,4]thiadiazin-7-yl)-3-phenylurea; NCGC00521878-01:

LC-MS t2 = 5.26 min, M+H = 415. 1H NMR (400 MHz, DMSO-d6) δ 9.14 (s, 1H), 8.80 (s, 1H), 8.11 (d, J = 2.4 Hz, 1H), 7.69 – 7.57 (m, 2H), 7.51 – 7.44 (m, 2H), 7.29 (t, J = 7.8 Hz, 2H), 7.04 – 6.95 (m, 1H), 4.13 – 4.05 (m, 2H), 2.65 (d, J = 7.0 Hz, 2H), 2.19 (dt, J = 13.5, 6.8 Hz, 1H), 1.69 (q, J = 7.6 Hz, 2H), 1.16 (s, 1H), 0.99 (d, J = 6.6 Hz, 6H), 0.94 (t, J = 7.3 Hz, 3H).

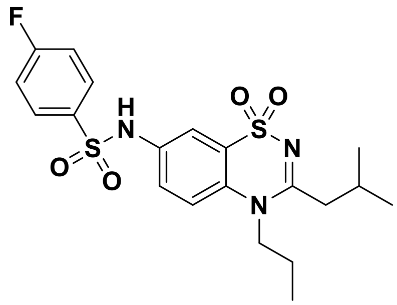

4-Fluoro-N-(3-isobutyl-1,1-dioxido-4-propyl-4H-benzo[e][1,2,4]thiadiazin-7-yl)benzene-sulfonamide; NCGC00521879-01:

LC-MS t2 = 5.28 min, M+H = 454.

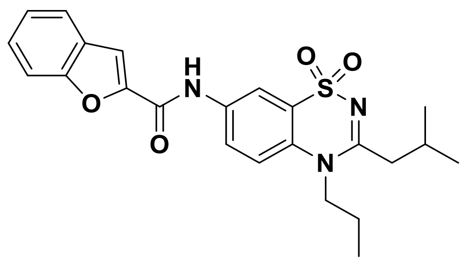

N-(3-Isobutyl-1,1-dioxido-4-propyl-4H-benzo[e][1,2,4]thiadiazin-7-yl)benzofuran-2-carboxamide; NCGC00521880-01:

LC-MS t2 = 5.64 min, M+H = 440.

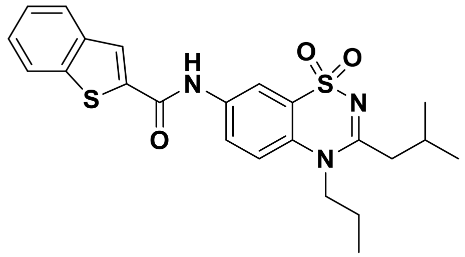

N-(3-Isobutyl-1,1-dioxido-4-propyl-4H-benzo[e][1,2,4]thiadiazin-7-yl)benzo[b]thiophene-2-carboxamide; NCGC00521881-01:

LC-MS t2 = 5.87 min, M+H = 456.

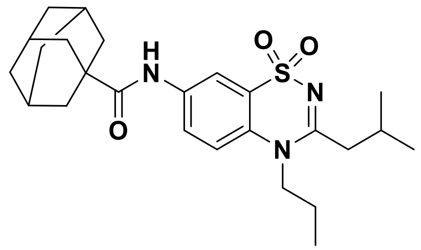

N-(3-isobutyl-1,1-dioxido-4-propyl-4H-benzo[e][1,2,4]thiadiazin-7-yl)adamantane-1-carboxamide; NCGC00521891-01:

LC-MS t2 = 6.28 min, M+H = 458.

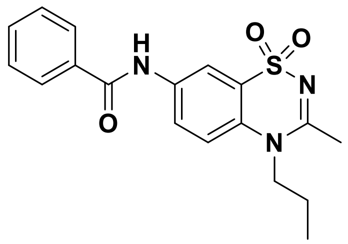

N-(3-methyl-1,1-dioxido-4-propyl-4H-benzo[e][1,2,4]thiadiazin-7-yl)benzamide; NCGC00522605-01:

LC-MS t2 = 4.40 min, M+H = 358. 1H NMR (400 MHz, DMSO-d6) δ 10.62 (s, 1H), 8.42 (d, J = 2.5 Hz, 1H), 8.13 (dd, J = 9.4, 2.5 Hz, 1H), 8.03 – 7.96 (m, 2H), 7.69 (d, J = 9.4 Hz, 1H), 7.63

(dd, J = 8.6, 6.0 Hz, 1H), 7.56 (dd, J = 8.3, 6.7 Hz, 2H), 4.08 (t, J = 7.9 Hz, 2H), 2.53 (s, 3H), 1.73 (q, J = 7.6 Hz, 2H), 0.96 (t, J = 7.3 Hz, 3H).

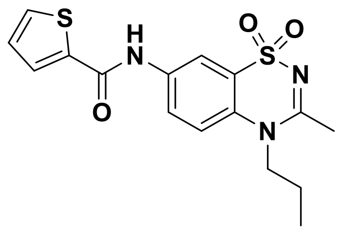

N-(3-Methyl-1,1-dioxido-4-propyl-4H-benzo[e][1,2,4]thiadiazin-7-yl)thiophene-2-carboxamide; NCGC00522606-01:

LC-MS t2 = 4.32 min, M+H = 364. 1H NMR (400 MHz, DMSO-d6) δ 10.59 (s, 1H), 8.30 (d, J = 2.5 Hz, 1H), 8.12 (dd, J = 9.4, 2.5 Hz, 1H), 8.05 (dd, J = 3.9, 1.2 Hz, 1H), 7.91 (dd, J = 5.0, 1.2 Hz, 1H), 7.69 (d, J = 9.4 Hz, 1H), 7.25 (dd, J = 5.0, 3.7 Hz, 1H), 4.08 (t, J = 7.9 Hz, 2H), 2.52 (s, 3H), 1.72 (p, J = 7.6 Hz, 2H), 0.96 (t, J = 7.3 Hz, 3H).

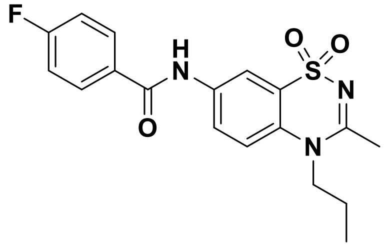

4-Fluoro-N-(3-methyl-1,1-dioxido-4-propyl-4H-benzo[e][1,2,4]thiadiazin-7-yl)benzamide; NCGC00522607-01:

LC-MS t2 = 4.52 min, M+H = 376. 1H NMR (400 MHz, DMSO-d6) δ 10.63 (s, 1H), 8.39 (d, J = 2.5 Hz, 1H), 8.15 – 8.04 (m, 3H), 7.69 (d, J = 9.4 Hz, 1H), 7.40 (t, J = 8.9 Hz, 2H), 4.08 (t, J = 7.8 Hz, 2H), 2.52 (s, 3H), 1.73 (q, J = 7.6 Hz, 2H), 0.96 (t, J = 7.4 Hz, 3H).

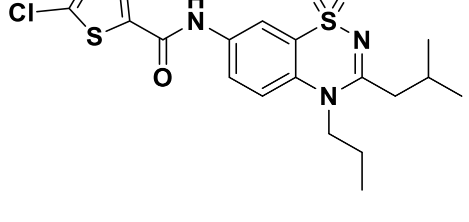

5-Chloro-N-(3-isobutyl-1,1-dioxido-4-propyl-4H-benzo[e][1,2,4]thiadiazin-7-yl)thiophene-2-carboxamide; NCGC00523535-01:

LC-MS t2 = 5.73 min, M+H = 440. 1H NMR (400 MHz, DMSO-d6) δ 10.67 (s, 1H), 8.26 (d, J = 2.6 Hz, 1H), 8.08 (dd, J = 9.3, 2.6 Hz, 1H), 7.94 (d, J = 4.2 Hz, 1H), 7.72 (d, J = 9.4 Hz, 1H), 7.30 (d, J = 4.1 Hz, 1H), 4.11 (t, J = 7.7 Hz, 2H), 2.67 (d, J = 6.9 Hz, 2H), 2.19 (hept, J = 6.7 Hz, 1H), 1.76 – 1.64 (m, 2H), 1.02 – 0.90 (m, 9H).

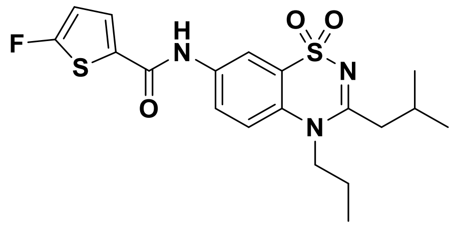

5-Fluoro-N-(3-isobutyl-1,1-dioxido-4-propyl-4H-benzo[e][1,2,4]thiadiazin-7-yl)thiophene-2-carboxamide; NCGC00523537-01:

LC-MS t2 = 5.47 min, M+H = 424. 1H NMR (400 MHz, DMSO-d6) δ 10.62 (s, 1H), 8.25 (d, J = 2.5 Hz, 1H), 8.08 (dd, J = 9.4, 2.6 Hz, 1H), 7.84 (dd, J = 4.3, 3.6 Hz, 1H), 7.71 (d, J = 9.4 Hz, 1H), 6.93 (dd, J = 4.3, 1.9 Hz, 1H), 4.11 (t, J = 7.7 Hz, 2H), 2.67 (d, J = 6.9 Hz, 2H), 2.19 (hept, J = 6.7 Hz, 1H), 1.69 (h, J = 7.5 Hz, 2H), 0.99 (d, J = 6.6 Hz, 6H), 0.94 (t, J = 7.3 Hz, 3H).

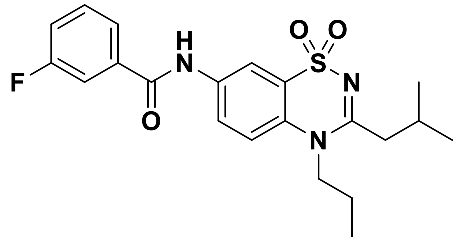

3-Fluoro-N-(3-isobutyl-1,1-dioxido-4-propyl-4H-benzo[e][1,2,4]thiadiazin-7-yl)benzamide; NCGC00523538-01:

LC-MS t2 = 5.40 min, M+H = 418. 1H NMR (400 MHz, DMSO-d6) δ 10.68 (s, 1H), 8.40 (d, J = 2.5 Hz, 1H), 8.12 (dd, J = 9.3, 2.6 Hz, 1H), 7.89 – 7.77 (m, 2H), 7.72 (d, J = 9.4 Hz, 1H), 7.62 (td, J = 8.0, 5.8 Hz, 1H), 7.48 (tdd, J = 8.3, 2.7, 1.0 Hz, 1H), 4.12 (t, J = 7.7 Hz, 2H), 2.68 (d, J = 7.0 Hz, 2H), 2.20 (hept, J = 6.7 Hz, 1H), 1.70 (h, J = 7.4 Hz, 2H), 0.99 (d, J = 6.6 Hz, 6H), 0.95 (t, J = 7.3 Hz, 3H).

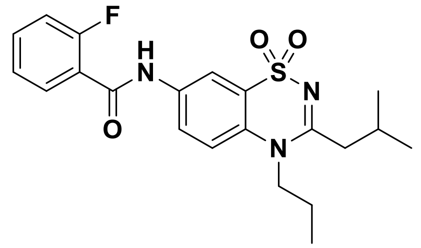

2-Fluoro-N-(3-isobutyl-1,1-dioxido-4-propyl-4H-benzo[e][1,2,4]thiadiazin-7-yl)benzamide; NCGC00523540-01:

LC-MS t2 = 5.28 min, M+H = 418. 1H NMR (400 MHz, DMSO-d6) δ 10.82 (s, 1H), 8.37 (d, J = 2.5 Hz, 1H), 7.97 (dd, J = 9.3, 2.6 Hz, 1H), 7.76 – 7.67 (m, 2H), 7.67 – 7.56 (m, 1H), 7.43 – 7.36 (m, 1H), 7.40 – 7.32 (m, 1H), 4.11 (t, J = 7.7 Hz, 2H), 3.29 (s, 0H), 2.68 (d, J = 7.0 Hz, 2H), 2.19 (hept, J = 6.8 Hz, 1H), 1.69 (hept, J = 7.2 Hz, 2H), 0.99 (d, J = 6.6 Hz, 6H), 0.94 (t, J = 7.3 Hz, 3H).

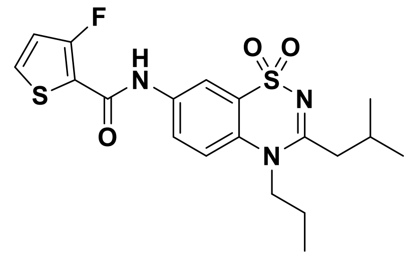

3-Fluoro-N-(3-isobutyl-1,1-dioxido-4-propyl-4H-benzo[e][1,2,4]thiadiazin-7-yl)thiophene-2-carboxamide; NCGC00523542-01:

LC-MS t2 = 5.24 min, M+H = 424. 1H NMR (400 MHz, DMSO-d6) δ 10.29 (s, 1H), 8.27 (d, J = 2.5 Hz, 1H), 8.01 (dd, J = 9.3, 2.6 Hz, 1H), 7.91 (dd, J = 5.5, 4.0 Hz, 1H), 7.70 (d, J = 9.4 Hz, 1H), 7.19 (d, J = 5.5 Hz, 1H), 4.11 (t, J = 7.7 Hz, 2H), 2.67 (d, J = 6.9 Hz, 2H), 2.19 (hept, J = 6.9 Hz, 1H), 1.68 (p, J = 7.5 Hz, 2H), 0.99 (d, J = 6.6 Hz, 6H), 0.94 (t, J = 7.4 Hz, 3H).

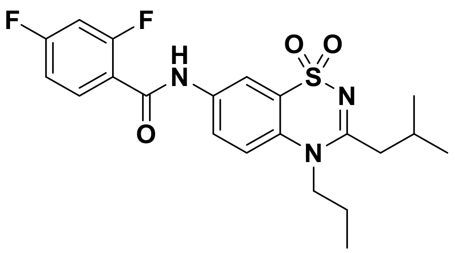

2,4-Difluoro-N-(3-isobutyl-1,1-dioxido-4-propyl-4H-benzo[e][1,2,4]thiadiazin-7-yl)benzamide; NCGC00523543-01:

LC-MS t2 = 5.36 min, M+H = 436. 1H NMR (400 MHz, DMSO-d6) δ 10.81 (s, 1H), 8.35 (d, J = 2.5 Hz, 1H), 7.96 (dd, J = 9.4, 2.5 Hz, 1H), 7.81 (td, J = 8.5, 6.5 Hz, 1H), 7.71 (d, J = 9.4 Hz, 1H), 7.46 (ddd, J = 10.6, 9.4, 2.4 Hz, 1H), 7.26 (td, J = 8.6, 2.5 Hz, 1H), 4.11 (t, J = 7.7 Hz, 2H), 2.68 (d, J = 7.0 Hz, 2H), 2.19 (hept, J = 6.8 Hz, 1H), 1.69 (q, J = 7.5 Hz, 2H), 0.99 (d, J = 6.6 Hz, 6H), 0.94 (t, J = 7.3 Hz, 3H).

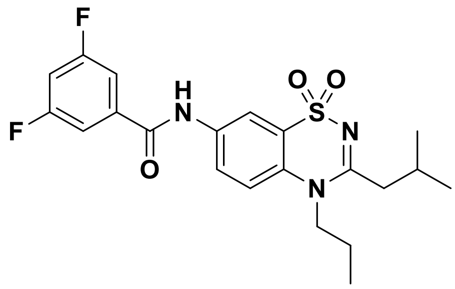

3,5-Difluoro-N-(3-isobutyl-1,1-dioxido-4-propyl-4H-benzo[e][1,2,4]thiadiazin-7-yl)benzamide; NCGC00523544-01:

LC-MS t2 = 5.55 min, M+H = 436. 1H NMR (400 MHz, DMSO-d6) δ 10.72 (s, 1H), 8.37 (d, J = 2.5 Hz, 1H), 8.10 (dd, J = 9.4, 2.5 Hz, 1H), 7.77 – 7.67 (m, 3H), 7.57 (tt, J = 9.2, 2.3 Hz, 1H), 4.12 (t, J = 7.6 Hz, 2H), 2.68 (d, J = 7.0 Hz, 2H), 2.19 (hept, J = 6.6 Hz, 1H), 1.69 (p, J = 7.5 Hz, 2H), 0.99 (d, J = 6.6 Hz, 6H), 0.94 (t, J = 7.3 Hz, 3H).

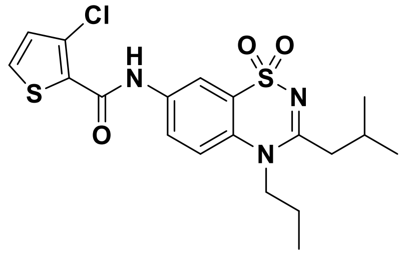

3-Chloro-N-(3-isobutyl-1,1-dioxido-4-propyl-4H-benzo[e][1,2,4]thiadiazin-7-yl)thiophene-2-carboxamide; NCGC00531529-01:

LC-MS t2 = 5.45 min, M+H = 440. 1H NMR (400 MHz, DMSO-d6) δ 10.63 (s, 1H), 8.28 (d, J = 2.6 Hz, 1H), 8.03 – 7.92 (m, 2H), 7.71 (d, J = 9.4 Hz, 1H), 7.24 (d, J = 5.2 Hz, 1H), 4.11 (t, J =

7.7 Hz, 2H), 2.68 (d, J = 6.9 Hz, 2H), 2.19 (hept, J = 6.8 Hz, 1H), 1.69 (q, J = 7.5 Hz, 2H), 0.99 (d, J = 6.6 Hz, 6H), 0.94 (t, J = 7.3 Hz, 3H).

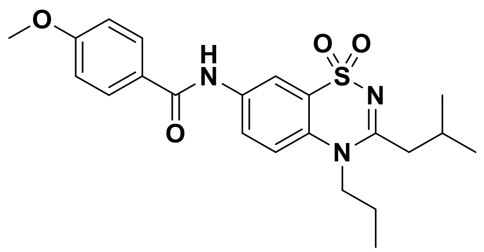

N-(3-Isobutyl-1,1-dioxido-4-propyl-4H-benzo[e][1,2,4]thiadiazin-7-yl)-4-methoxybenzamide; NCGC00531533-01:

LC-MS t2 = 5.28 min, M+H = 430. 1H NMR (400 MHz, DMSO-d6) δ 10.47 (s, 1H), 8.39 (d, J = 2.5 Hz, 1H), 8.13 (dd, J = 9.3, 2.5 Hz, 1H), 8.03 – 7.96 (m, 2H), 7.70 (d, J = 9.4 Hz, 1H), 7.12 – 7.05 (m, 2H), 4.11 (t, J = 7.7 Hz, 2H), 3.85 (s, 3H), 2.67 (d, J = 7.0 Hz, 2H), 2.19 (dq, J = 13.4, 6.7 Hz, 1H), 1.70 (q, J = 7.5 Hz, 2H), 0.99 (d, J = 6.6 Hz, 6H), 0.94 (t, J = 7.3 Hz, 3H).

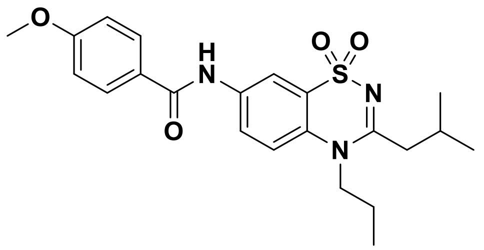

N-(3-Isobutyl-1,1-dioxido-4-propyl-4H-benzo[e][1,2,4]thiadiazin-7-yl)-4-methoxybenzamide; NCGC00531533-02:

LC-MS t2 = 5.34 min, M+H = 430. 1H NMR (400 MHz, DMSO-d6) δ 10.46 (s, 1H), 8.39 (d, J = 2.5 Hz, 1H), 8.13 (dd, J = 9.4, 2.5 Hz, 1H), 8.00 (d, J = 8.7 Hz, 2H), 7.69 (d, J = 9.4 Hz, 1H), 7.12 – 7.06 (m, 2H), 4.11 (t, J = 7.7 Hz, 2H), 3.85 (s, 3H), 2.67 (d, J = 6.7 Hz, 2H), 2.20 (dt, J = 13.4, 6.7 Hz, 1H), 1.70 (q, J = 7.6 Hz, 2H), 0.99 (d, J = 6.6 Hz, 6H), 0.94 (t, J = 7.3 Hz, 3H).

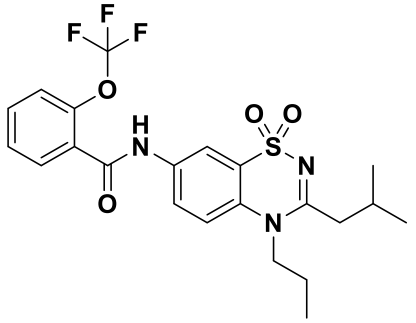

N-(3-Isobutyl-1,1-dioxido-4-propyl-4H-benzo[e][1,2,4]thiadiazin-7-yl)-2-(trifluoromethoxy)benzamide; NCGC00531535-01:

LC-MS t2 = 5.58 min, M+H = 484. 1H NMR (400 MHz, DMSO-d6) δ 10.91 (s, 1H), 8.35 (d, J = 2.5 Hz, 1H), 7.93 (dd, J = 9.4, 2.5 Hz, 1H), 7.77 (dd, J = 7.6, 1.7 Hz, 1H), 7.74 – 7.64 (m, 2H), 7.61 – 7.51 (m, 2H), 4.11 (t, J = 7.7 Hz, 2H), 2.68 (d, J = 7.0 Hz, 2H), 2.19 (hept, J = 6.7 Hz, 1H), 1.68 (p, J = 7.6 Hz, 2H), 0.99 (d, J = 6.6 Hz, 6H), 0.94 (t, J = 7.3 Hz, 3H).

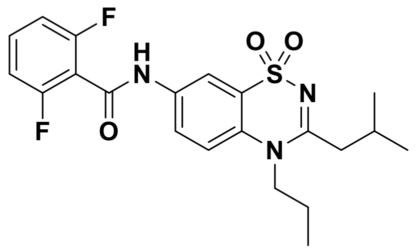

2,6-Difluoro-N-(3-isobutyl-1,1-dioxido-4-propyl-4H-benzo[e][1,2,4]thiadiazin-7-yl)benzamide; NCGC00531536-01:

LC-MS t2 = 5.22 min, M+H = 436. 1H NMR (400 MHz, DMSO-d6) δ 11.23 (s, 1H), 8.34 (d, J = 2.5 Hz, 1H), 7.89 (dd, J = 9.3, 2.5 Hz, 1H), 7.73 (d, J = 9.4 Hz, 1H), 7.63 (tt, J = 8.4, 6.7 Hz, 1H), 7.29 (t, J = 8.1 Hz, 2H), 4.11 (t, J = 7.7 Hz, 2H), 2.68 (d, J = 7.1 Hz, 2H), 2.19 (hept, J = 6.7 Hz, 1H), 1.68 (p, J = 7.3 Hz, 2H), 0.99 (d, J = 6.6 Hz, 6H), 0.94 (t, J = 7.3 Hz, 3H).

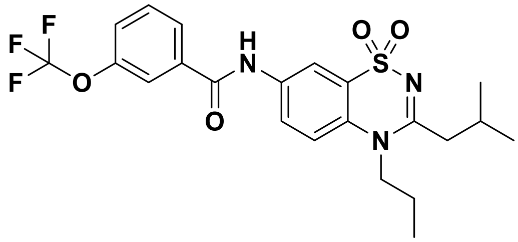

N-(3-Isobutyl-1,1-dioxido-4-propyl-4H-benzo[e][1,2,4]thiadiazin-7-yl)-3-(trifluoromethoxy)benzamide; NCGC00531537-01:

LC-MS t2 = 5.88 min, M+H = 484. 1H NMR (400 MHz, DMSO-d6) δ 10.74 (s, 1H), 8.38 (d, J = 2.5 Hz, 1H), 8.12 (dd, J = 9.3, 2.5 Hz, 1H), 8.05 (dt, J = 7.7, 1.4 Hz, 1H), 7.96 (s, 1H), 7.77 – 7.68 (m, 2H), 7.68 – 7.61 (m, 1H), 4.12 (t, J = 7.7 Hz, 2H), 2.68 (d, J = 7.1 Hz, 2H), 2.20 (hept, J = 6.7 Hz, 1H), 1.70 (q, J = 7.5 Hz, 2H), 1.00 (d, J = 6.6 Hz, 6H), 0.95 (t, J = 7.3 Hz, 3H).

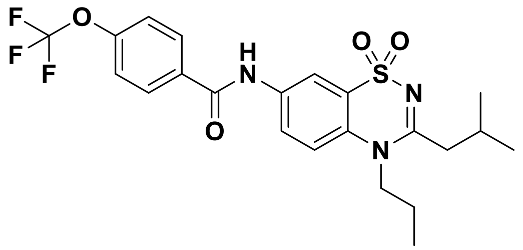

N-(3-Isobutyl-1,1-dioxido-4-propyl-4H-benzo[e][1,2,4]thiadiazin-7-yl)-4-(trifluoromethoxy)benzamide; NCGC00531538-01:

LC-MS t2 = 5.88 min, M+H = 484. 1H NMR (400 MHz, DMSO-d6) δ 10.73 (s, 1H), 8.39 (d, J = 2.5 Hz, 1H), 8.16 – 8.07 (m, 3H), 7.72 (d, J = 9.4 Hz, 1H), 7.56 (d, J = 8.3 Hz, 2H), 4.12 (t, J = 7.7 Hz, 2H), 2.68 (d, J = 7.0 Hz, 2H), 2.20 (hept, J = 6.7 Hz, 1H), 1.70 (q, J = 7.5 Hz, 2H), 0.99 (d, J = 6.6 Hz, 6H), 0.94 (t, J = 7.3 Hz, 3H).

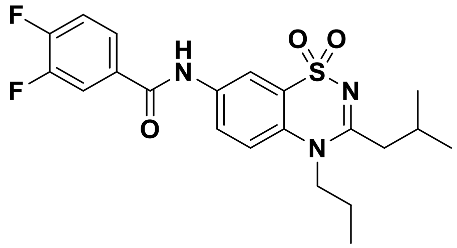

3,4-Difluoro-N-(3-isobutyl-1,1-dioxido-4-propyl-4H-benzo[e][1,2,4]thiadiazin-7-yl)benzamide; NCGC00531540-01:

LC-MS t2 = 5.48 min, M+H = 436. 1H NMR (400 MHz, DMSO-d6) δ 10.68 (s, 1H), 8.37 (d, J = 2.5 Hz, 1H), 8.14 – 8.07 (m, 1H), 8.11 – 8.03 (m, 1H), 7.90 (s, 1H), 7.73 (d, J = 9.4 Hz, 1H), 7.66 (dt, J = 10.4, 8.2 Hz, 1H), 4.12 (t, J = 7.8 Hz, 2H), 2.68 (d, J = 7.1 Hz, 2H), 2.19 (p, J = 6.7 Hz, 1H), 1.70 (q, J = 7.5 Hz, 2H), 0.99 (d, J = 6.6 Hz, 6H), 0.94 (t, J = 7.3 Hz, 3H).

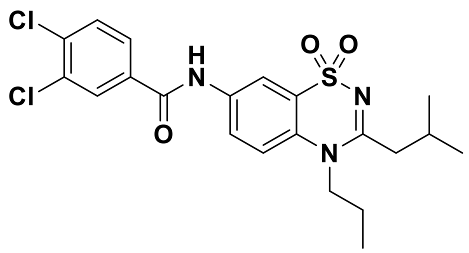

3,4-Dichloro-N-(3-isobutyl-1,1-dioxido-4-propyl-4H-benzo[e][1,2,4]thiadiazin-7-yl)benzamide; NCGC00531756-01:

LC-MS t2 = 6.05 min, M+H = 469. 1H NMR (400 MHz, DMSO-d6) δ 10.74 (s, 1H), 8.37 (d, J = 2.5 Hz, 1H), 8.26 (d, J = 2.0 Hz, 1H), 8.11 (dd, J = 9.3, 2.5 Hz, 1H), 7.97 (dd, J = 8.5, 2.1 Hz, 1H), 7.85 (d, J = 8.4 Hz, 1H), 7.73 (d, J = 9.4 Hz, 1H), 4.12 (t, J = 7.7 Hz, 2H), 2.68 (d, J = 7.0 Hz, 2H), 2.20 (dq, J = 13.4, 6.8 Hz, 1H), 1.70 (q, J = 7.6 Hz, 2H), 0.99 (d, J = 6.6 Hz, 6H), 0.95 (t, J = 7.3 Hz, 3H).

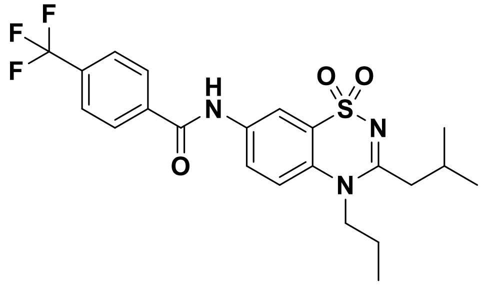

N-(3-Isobutyl-1,1-dioxido-4-propyl-4H-benzo[e][1,2,4]thiadiazin-7-yl)-4-(trifluoromethyl)benzamide; NCGC00531759-01:

LC-MS t2 = 5.89 min, M+H = 468. 1H NMR (400 MHz, DMSO-d6) δ 10.83 (s, 1H), 8.40 (d, J = 2.4 Hz, 1H), 8.19 (d, J = 8.1 Hz, 2H), 8.12 (dd, J = 9.3, 2.5 Hz, 1H), 7.95 (d, J = 8.1 Hz, 2H), 7.73 (d, J = 9.4 Hz, 1H), 4.12 (t, J = 7.7 Hz, 2H), 2.68 (d, J = 7.1 Hz, 2H), 2.20 (hept, J = 6.7 Hz, 1H), 1.70 (q, J = 7.6 Hz, 2H), 1.03 – 0.91 (m, 9H).

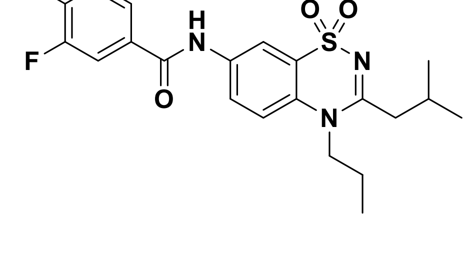

4-Bromo-3-fluoro-N-(3-isobutyl-1,1-dioxido-4-propyl-4H-benzo[e][1,2,4]thiadiazin-7-yl)benzamide; NCGC00531761-01:

LC-MS t2 = 5.88 min, M+H = 497. 1H NMR (400 MHz, DMSO-d6) δ 10.72 (s, 1H), 8.37 (d, J = 2.4 Hz, 1H), 8.11 (dd, J = 9.4, 2.5 Hz, 1H), 8.02 – 7.90 (m, 2H), 7.80 (dd, J = 8.2, 2.0 Hz, 1H), 7.73 (d, J = 9.4 Hz, 1H), 4.16 – 4.08 (m, 2H), 2.68 (d, J = 7.0 Hz, 2H), 2.19 (dq, J = 13.4, 6.7 Hz, 1H), 1.70 (q, J = 7.5 Hz, 2H), 1.03 – 0.90 (m, 9H).

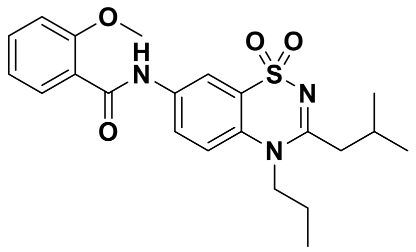

N-(3-Isobutyl-1,1-dioxido-4-propyl-4H-benzo[e][1,2,4]thiadiazin-7-yl)-2-methoxybenzamide; NCGC00531762-01:

LC-MS t2 = 5.55 min, M+H = 430. 1H NMR (400 MHz, DMSO-d6) δ 10.49 (s, 1H), 8.40 (d, J = 2.4 Hz, 1H), 7.98 (dd, J = 9.4, 2.5 Hz, 1H), 7.68 (d, J = 9.4 Hz, 1H), 7.62 (dd, J = 7.6, 1.8 Hz, 1H), 7.52 (ddd, J = 9.0, 7.3, 1.8 Hz, 1H), 7.19 (d, J = 8.4 Hz, 1H), 7.08 (td, J = 7.5, 1.0 Hz, 1H), 4.11 (t, J = 7.6 Hz, 2H), 3.89 (s, 3H), 2.67 (d, J = 6.9 Hz, 2H), 2.19 (hept, J = 6.8 Hz, 1H), 1.69 (q, J = 7.6 Hz, 2H), 1.02 – 0.90 (m, 9H).

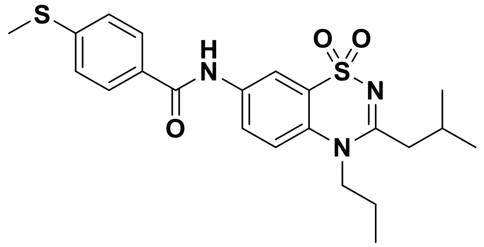

N-(3-Isobutyl-1,1-dioxido-4-propyl-4H-benzo[e][1,2,4]thiadiazin-7-yl)-4-(methylthio)benzamide; NCGC00531764-01:

LC-MS t2 = 5.64 min, M+H = 446. 1H NMR (400 MHz, DMSO-d6) δ 10.55 (s, 1H), 8.39 (d, J = 2.5 Hz, 1H), 8.13 (dd, J = 9.4, 2.5 Hz, 1H), 7.99 – 7.92 (m, 2H), 7.70 (d, J = 9.4 Hz, 1H), 7.41 (d, J = 8.4 Hz, 2H), 4.12 (t, J = 7.8 Hz, 2H), 2.67 (d, J = 6.7 Hz, 2H), 2.55 (s, 3H), 2.20 (dt, J = 13.5, 6.7 Hz, 1H), 1.70 (q, J = 7.6 Hz, 2H), 1.03 – 0.90 (m, 9H).

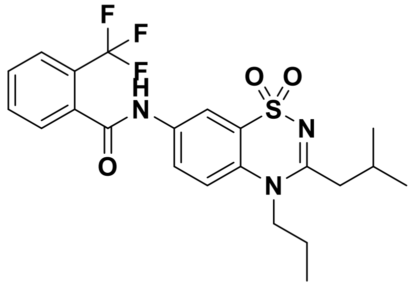

N-(3-Isobutyl-1,1-dioxido-4-propyl-4H-benzo[e][1,2,4]thiadiazin-7-yl)-2-(trifluoromethyl)benzamide:

LC-MS t2 = 5.49 min, M+H = 468. 1H NMR (400 MHz, DMSO-d6) δ 10.98 (s, 1H), 8.34 (d, J = 2.4 Hz, 1H), 7.94 – 7.86 (m, 2H), 7.86 – 7.79 (m, 1H), 7.79 – 7.68 (m, 3H), 4.11 (t, J = 7.7 Hz, 2H), 2.68 (d, J = 7.0 Hz, 2H), 2.20 (hept, J = 6.6 Hz, 1H), 1.69 (q, J = 7.6 Hz, 2H), 1.02 – 0.90 (m, 9H).

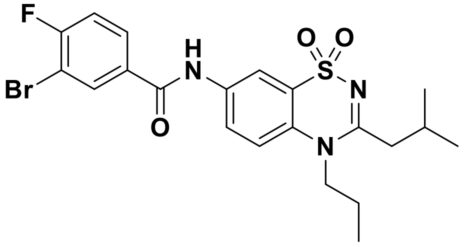

3-Bromo-4-fluoro-N-(3-isobutyl-1,1-dioxido-4-propyl-4H-benzo[e][1,2,4]thiadiazin-7-yl)benzamide; NCGC00531767-01:

LC-MS t2 = 5.83 min, M+H = 497.

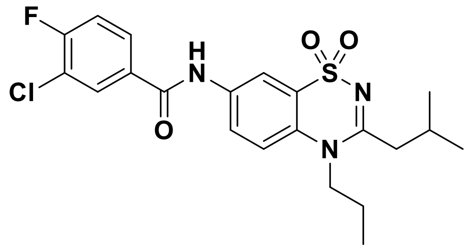

3-Chloro-4-fluoro-N-(3-isobutyl-1,1-dioxido-4-propyl-4H-benzo[e][1,2,4]thiadiazin-7-yl)benzamide; NCGC00531769-01:

LC-MS t2 = 5.77 min, M+H = 452. 1H NMR (400 MHz, DMSO-d6) δ 10.70 (s, 1H), 8.36 (d, J = 2.5 Hz, 1H), 8.25 (dd, J = 7.1, 2.2 Hz, 1H), 8.10 (dd, J = 9.4, 2.5 Hz, 1H), 8.03 (ddd, J = 8.5,

4.7, 2.2 Hz, 1H), 7.72 (d, J = 9.4 Hz, 1H), 7.63 (t, J = 8.9 Hz, 1H), 4.12 (t, J = 7.7 Hz, 2H), 2.68 (d, J = 7.0 Hz, 2H), 2.20 (dt, J = 13.4, 6.7 Hz, 1H), 1.70 (q, J = 7.6 Hz, 2H), 0.99 (d, J = 6.6 Hz, 6H), 0.95 (t, J = 7.3 Hz, 3H).

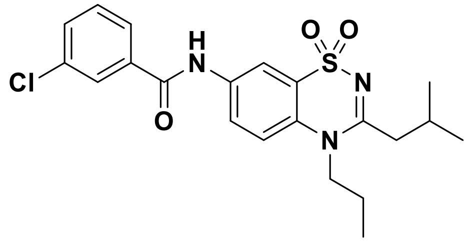

3-Chloro-N-(3-isobutyl-1,1-dioxido-4-propyl-4H-benzo[e][1,2,4]thiadiazin-7-yl)benzamide; NCGC00531770-01:

LC-MS t2 = 5.70 min, M+H = 434. 1H NMR (400 MHz, DMSO-d6) δ 10.71 (s, 1H), 8.39 (d, J = 2.5 Hz, 1H), 8.11 (dd, J = 9.3, 2.5 Hz, 1H), 8.06 (t, J = 1.9 Hz, 1H), 7.95 (dt, J = 7.8, 1.3 Hz, 1H), 7.76 – 7.66 (m, 2H), 7.60 (t, J = 7.9 Hz, 1H), 4.12 (t, J = 7.6 Hz, 2H), 2.68 (d, J = 7.0 Hz, 2H), 2.20 (hept, J = 6.8 Hz, 1H), 1.70 (q, J = 7.6 Hz, 2H), 1.03 – 0.90 (m, 9H).

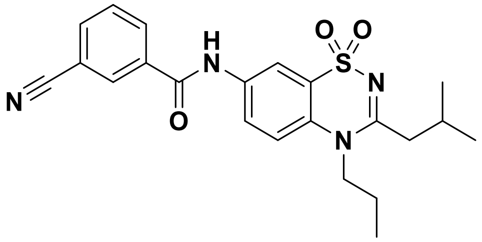

3-Cyano-N-(3-isobutyl-1,1-dioxido-4-propyl-4H-benzo[e][1,2,4]thiadiazin-7-yl)benzamide; NCGC00531772-01:

LC-MS t2 = 5.26 min, M+H = 425. 1H NMR (400 MHz, DMSO-d6) δ 10.78 (s, 1H), 8.45 (s, 1H), 8.38 (d, J = 2.5 Hz, 1H), 8.32 – 8.25 (m, 1H), 8.10 (ddd, J = 8.0, 3.9, 1.9 Hz, 2H), 7.83 – 7.70 (m, 2H), 4.12 (t, J = 7.8 Hz, 2H), 2.68 (d, J = 7.1 Hz, 2H), 2.20 (dt, J = 13.5, 6.8 Hz, 1H), 1.70 (q, J = 7.5 Hz, 2H), 1.03 – 0.91 (m, 9H).

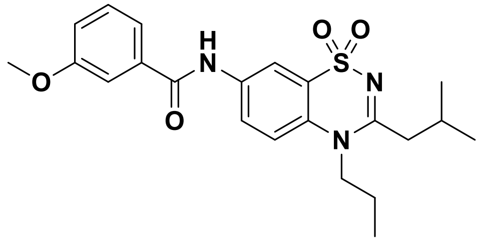

N-(3-Isobutyl-1,1-dioxido-4-propyl-4H-benzo[e][1,2,4]thiadiazin-7-yl)-3-methoxybenzamide; NCGC00531773-01:

LC-MS t2 = 5.40 min, M+H = 430. 1H NMR (400 MHz, DMSO-d6) δ 10.58 (s, 1H), 8.39 (d, J = 2.5 Hz, 1H), 8.13 (dd, J = 9.4, 2.5 Hz, 1H), 7.71 (d, J = 9.4 Hz, 1H), 7.61 – 7.55 (m, 1H), 7.53 (dd, J = 2.7, 1.5 Hz, 1H), 7.47 (t, J = 7.9 Hz, 1H), 7.19 (ddd, J = 8.1, 2.7, 1.0 Hz, 1H), 4.12 (t, J = 7.8 Hz, 2H), 3.85 (s, 3H), 2.68 (d, J = 6.9 Hz, 2H), 2.20 (dt, J = 13.5, 6.7 Hz, 1H), 1.70 (q, J = 7.6 Hz, 2H), 1.00 (d, J = 6.6 Hz, 6H), 0.95 (t, J = 7.3 Hz, 3H).

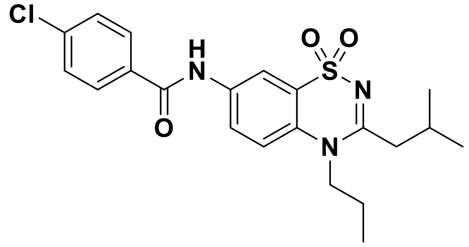

4-Chloro-N-(3-isobutyl-1,1-dioxido-4-propyl-4H-benzo[e][1,2,4]thiadiazin-7-yl)benzamide; NCGC00531775-01:

LC-MS t2 = 5.67 min, M+H = 434. 1H NMR (400 MHz, DMSO-d6) δ 10.68 (s, 1H), 8.39 (d, J = 2.5 Hz, 1H), 8.11 (dd, J = 9.4, 2.5 Hz, 1H), 8.06 – 7.99 (m, 2H), 7.72 (d, J = 9.4 Hz, 1H), 7.64 (d, J = 8.4 Hz, 2H), 4.12 (t, J = 7.7 Hz, 2H), 2.68 (d, J = 7.0 Hz, 2H), 2.20 (hept, J = 6.8 Hz, 1H), 1.70 (q, J = 7.6 Hz, 2H), 1.01 – 0.92 (m, 9H).

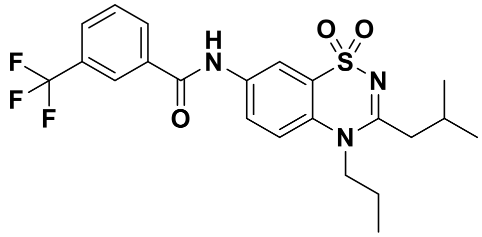

N-(3-isobutyl-1,1-dioxido-4-propyl-4H-benzo[e][1,2,4]thiadiazin-7-yl)-3-(trifluoromethyl)benzamide; NCGC00531777-01:

LC-MS t2 = 5.86 min, M+H = 468. 1H NMR (400 MHz, DMSO-d6) δ 10.82 (s, 1H), 8.40 – 8.36 (m, 1H), 8.36 – 8.27 (m, 2H), 8.14 (dd, J = 9.4, 2.5 Hz, 1H), 8.00 (d, J = 7.8 Hz, 1H), 7.82 (t, J = 7.9 Hz, 1H), 7.74 (d, J = 9.4 Hz, 1H), 4.12 (t, J = 7.6 Hz, 2H), 2.68 (d, J = 7.0 Hz, 2H), 2.20 (hept, J = 6.8 Hz, 1H), 1.71 (q, J = 7.5 Hz, 2H), 1.03 – 0.91 (m, 9H).

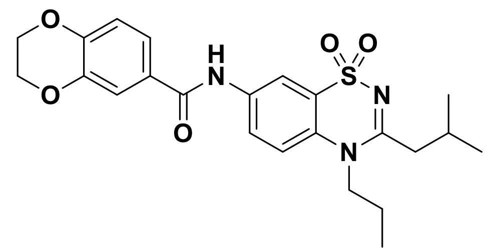

N-(3-Isobutyl-1,1-dioxido-4-propyl-4H-benzo[e][1,2,4]thiadiazin-7-yl)-2,3-dihydrobenzo[b][1,4]dioxine-6-carboxamide; NCGC00531778-01:

LC-MS t2 = 5.28 min, M+H = 458. 1H NMR (400 MHz, DMSO-d6) δ 10.42 (s, 1H), 8.39 (d, J = 2.5 Hz, 1H), 8.11 (dd, J = 9.3, 2.6 Hz, 1H), 7.71 (dd, J = 15.5, 9.4 Hz, 1H), 7.64 – 7.50 (m, 2H), 7.10 – 6.98 (m, 1H), 4.40 – 4.28 (m, 4H), 4.11 (t, J = 7.6 Hz, 2H), 2.67 (d, J = 7.0 Hz, 2H), 2.18 (dq, J = 13.6, 6.9 Hz, 1H), 1.69 (q, J = 7.6 Hz, 2H), 0.99 (d, J = 6.6 Hz, 6H), 0.94 (t, J = 7.3 Hz, 3H).

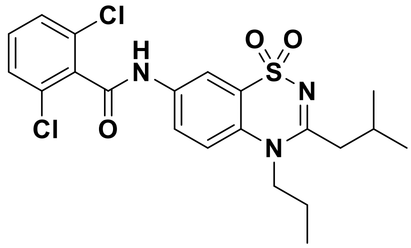

2,6-Dichloro-N-(3-isobutyl-1,1-dioxido-4-propyl-4H-benzo[e][1,2,4]thiadiazin-7-yl)benzamide; NCGC00531779-01:

LC-MS t2 = 5.51 min, M+H = 469. 1H NMR (400 MHz, DMSO-d6) δ 11.20 (s, 1H), 8.36 (d, J = 2.5 Hz, 1H), 7.87 (dd, J = 9.3, 2.5 Hz, 1H), 7.73 (d, J = 9.3 Hz, 1H), 7.65 – 7.59 (m, 2H), 7.54 (dd, J = 9.2, 7.0 Hz, 1H), 4.12 (t, J = 7.7 Hz, 2H), 2.68 (d, J = 7.1 Hz, 2H), 2.20 (hept, J = 6.7 Hz, 1H), 1.69 (q, J = 7.5 Hz, 2H), 0.99 (d, J = 6.6 Hz, 6H), 0.93 (t, J = 7.3 Hz, 3H).

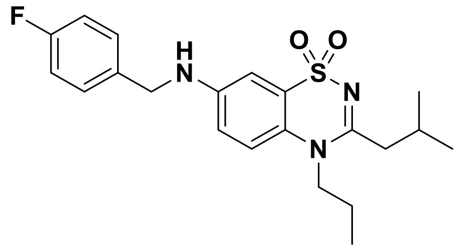

7-((4-Fluorobenzyl)amino)-3-isobutyl-4-propyl-4H-benzo[e][1,2,4]thiadiazine 1,1-dioxide; NCGC00531784-01:

LC-MS t2 = 5.64 min, M+H = 404. 1H NMR (400 MHz, DMSO-d6) δ 7.43 – 7.32 (m, 3H), 7.21 – 7.12 (m, 2H), 6.99 – 6.82 (m, 3H), 4.32 (d, J = 5.9 Hz, 2H), 4.00 (t, J = 7.7 Hz, 2H), 2.57 (d, J = 7.0 Hz, 2H), 2.14 (dt, J = 13.4, 6.6 Hz, 1H), 1.62 (q, J = 7.4 Hz, 2H), 1.08 – 0.79 (m, 11H).

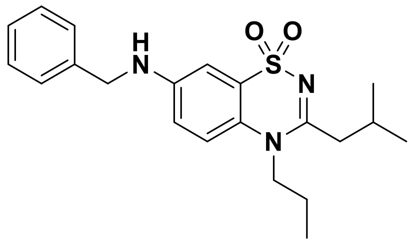

7-(Benzylamino)-3-isobutyl-4-propyl-4H-benzo[e][1,2,4]thiadiazine 1,1-dioxide; NCGC00531800-01:

LC-MS t2 = 5.58 min, M+H = 386.

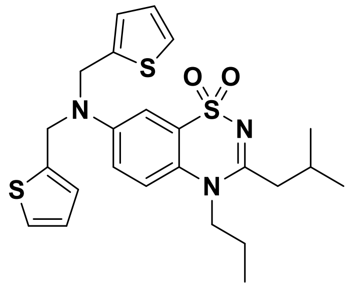

7-(Bis(thiophen-2-ylmethyl)amino)-3-isobutyl-4-propyl-4H-benzo[e][1,2,4]thiadiazine 1,1-dioxide; NCGC00531812-01:

LC-MS t2 = 6.25 min, M+H = 488. 1H NMR (400 MHz, DMSO-d6) δ 7.48 (d, J = 9.5 Hz, 1H), 7.40 (dd, J = 5.1, 1.3 Hz, 2H), 7.25 (dd, J = 9.5, 3.1 Hz, 1H), 7.12 (d, J = 3.1 Hz, 1H), 7.06 (dd, J = 3.4, 1.2 Hz, 2H), 6.99 (dd, J = 5.1, 3.4 Hz, 2H), 4.87 (s, 4H), 4.03 (t, J = 7.7 Hz, 2H), 2.59

(d, J = 7.0 Hz, 2H), 2.15 (hept, J = 6.8 Hz, 1H), 1.63 (h, J = 7.4 Hz, 2H), 0.96 (d, J = 6.6 Hz, 6H), 0.90 (t, J = 7.3 Hz, 3H).

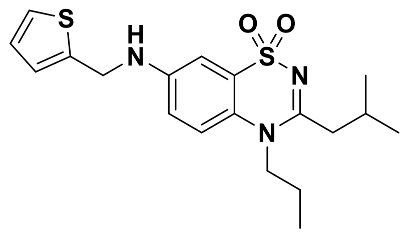

3-Isobutyl-4-propyl-7-((thiophen-2-ylmethyl)amino)-4H-benzo[e][1,2,4]thiadiazine 1,1-dioxide; NCGC00531814-01:

LC-MS t2 = 5.45 min, M+H = 392.

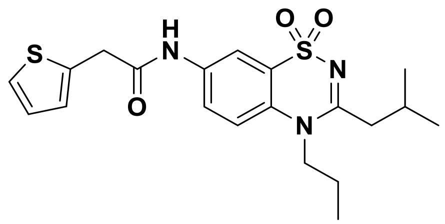

N-(3-Isobutyl-1,1-dioxido-4-propyl-4H-benzo[e][1,2,4]thiadiazin-7-yl)-2-(thiophen-2-yl)acetamide; NCGC00531815-01:

LC-MS t2 = 5.16 min, M+H = 420. 1H NMR (400 MHz, DMSO-d6) δ 10.62 (s, 1H), 8.23 (d, J = 2.5 Hz, 1H), 7.82 (dd, J = 9.3, 2.5 Hz, 1H), 7.66 (d, J = 9.4 Hz, 1H), 7.41 (dd, J = 5.0, 1.5 Hz, 1H), 6.99 (dd, J = 8.0, 2.9 Hz, 2H), 4.09 (t, J = 7.7 Hz, 2H), 3.91 (s, 2H), 2.65 (d, J = 7.3 Hz, 2H), 2.18 (dt, J = 13.5, 6.6 Hz, 1H), 1.67 (q, J = 7.6 Hz, 2H), 0.98 (d, J = 6.6 Hz, 6H), 0.93 (t, J = 7.3 Hz, 3H).

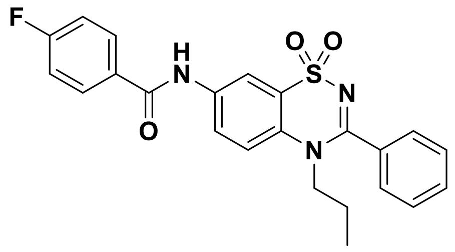

N-(1,1-dioxido-3-phenyl-4-propyl-4H-benzo[e][1,2,4]thiadiazin-7-yl)-4-fluorobenzamide; NCGC00532331-01:

LC-MS t2 = 5.27 min, M+H = 438. 1H NMR (400 MHz, DMSO-d6) δ 10.70 (s, 1H), 8.45 (d, J = 2.5 Hz, 1H), 8.20 (dd, J = 9.3, 2.5 Hz, 1H), 8.15 – 8.05 (m, 2H), 7.88 (d, J = 9.3 Hz, 1H), 7.73 – 7.66 (m, 2H), 7.69 – 7.55 (m, 3H), 7.47 – 7.36 (m, 2H), 4.13 (t, J = 6.6 Hz, 2H), 1.53 (h, J = 7.0 Hz, 2H), 0.63 (t, J = 7.3 Hz, 3H).

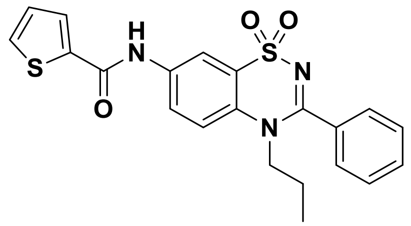

N-(1,1-Dioxido-3-phenyl-4-propyl-4H-benzo[e][1,2,4]thiadiazin-7-yl)thiophene-2-carboxamide; NCGC00532332-01:

LC-MS t2 = 5.07 min, M+H = 426. 1H NMR (400 MHz, DMSO-d6) δ 10.66 (s, 1H), 8.37 (d, J = 2.5 Hz, 1H), 8.19 (dd, J = 9.3, 2.5 Hz, 1H), 8.08 (dd, J = 3.8, 1.1 Hz, 1H), 7.92 (dd, J = 5.0, 1.1 Hz, 1H), 7.88 (d, J = 9.3 Hz, 1H), 7.72 – 7.67 (m, 2H), 7.67 – 7.56 (m, 3H), 7.27 (dd, J = 5.0, 3.8 Hz, 1H), 4.13 (t, J = 6.6 Hz, 2H), 1.53 (h, J = 7.1 Hz, 2H), 0.63 (t, J = 7.3 Hz, 3H).

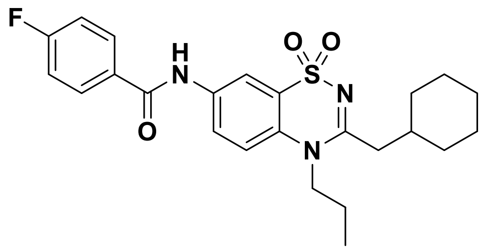

N-(3-(Cyclohexylmethyl)-1,1-dioxido-4-propyl-4H-benzo[e][1,2,4]thiadiazin-7-yl)-4-fluorobenzamide; NCGC00532335-01:

LC-MS t2 = 5.95 min, M+H = 458. 1H NMR (400 MHz, DMSO-d6) δ 10.63 (s, 1H), 8.39 (d, J = 2.5 Hz, 1H), 8.09 (ddd, J = 14.3, 9.1, 4.1 Hz, 3H), 7.71 (d, J = 9.4 Hz, 1H), 7.45 – 7.36 (m, 2H), 4.11 (t, J = 7.7 Hz, 2H), 2.66 (d, J = 6.8 Hz, 2H), 1.95 – 1.58 (m, 7H), 1.32 – 0.98 (m, 6H), 0.94 (t, J = 7.3 Hz, 3H).

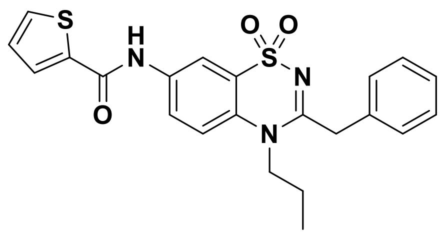

N-(3-Benzyl-1,1-dioxido-4-propyl-4H-benzo[e][1,2,4]thiadiazin-7-yl)thiophene-2-carboxamide; NCGC00532337-01:

LC-MS t2 = 5.25 min, M+H = 440. 1H NMR (400 MHz, DMSO-d6) δ 10.61 (s, 1H), 8.33 (d, J = 2.5 Hz, 1H), 8.11 (dd, J = 9.3, 2.6 Hz, 1H), 8.05 (dd, J = 3.8, 1.2 Hz, 1H), 7.91 (dd, J = 5.0, 1.1 Hz, 1H), 7.71 (d, J = 9.4 Hz, 1H), 7.38 (d, J = 5.2 Hz, 4H), 7.30 (ddt, J = 6.4, 4.5, 3.1 Hz, 1H), 7.26 (dd, J = 5.0, 3.8 Hz, 1H), 4.19 (s, 2H), 4.08 (t, J = 7.8 Hz, 2H), 1.53 (q, J = 7.5 Hz, 2H), 0.79 (t, J = 7.3 Hz, 3H).

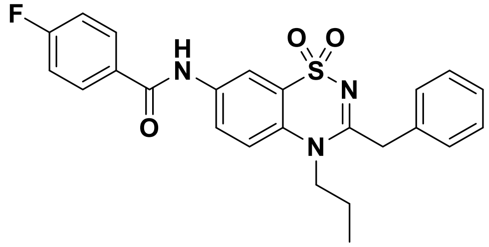

N-(3-Benzyl-1,1-dioxido-4-propyl-4H-benzo[e][1,2,4]thiadiazin-7-yl)-4-fluorobenzamide; NCGC00532338-01:

LC-MS t2 = 5.43 min, M+H = 452. 1H NMR (400 MHz, DMSO-d6) δ 10.65 (s, 1H), 8.42 (d, J = 2.5 Hz, 1H), 8.15 – 8.04 (m, 3H), 7.71 (d, J = 9.4 Hz, 1H), 7.45 – 7.36 (m, 5H), 7.39 – 7.26 (m, 1H), 4.19 (s, 2H), 4.08 (t, J = 7.8 Hz, 2H), 1.52 (p, J = 7.7 Hz, 2H), 0.79 (t, J = 7.3 Hz, 3H).

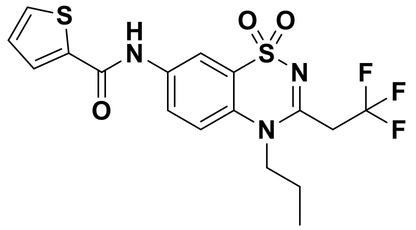

N-(1,1-Dioxido-4-propyl-3-(2,2,2-trifluoroethyl)-4H-benzo[e][1,2,4]thiadiazin-7-yl)thiophene-2-carboxamide; NCGC00532340-01:

LC-MS t2 = 4.92 min, M+H = 432.

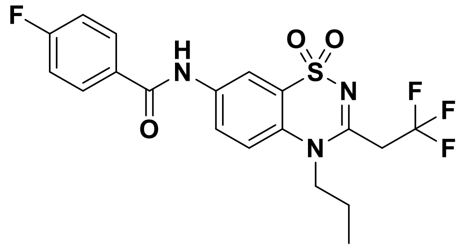

N-(1,1-Dioxido-4-propyl-3-(2,2,2-trifluoroethyl)-4H-benzo[e][1,2,4]thiadiazin-7-yl)-4-fluorobenzamide; NCGC00532346-01:

LC-MS t2 = 5.10 min, M+H = 444. 1H NMR (400 MHz, DMSO-d6) δ 10.68 (s, 1H), 8.43 (d, J = 2.5 Hz, 1H), 8.15 (dd, J = 9.3, 2.5 Hz, 1H), 8.11 – 8.04 (m, 2H), 7.77 (d, J = 9.4 Hz, 1H), 7.41 (t, J = 8.8 Hz, 2H), 4.25 (d, J = 9.9 Hz, 1H), 4.20 (d, J = 10.0 Hz, 1H), 4.13 (t, J = 7.8 Hz, 2H), 1.70 (q, J = 7.5 Hz, 2H), 0.94 (t, J = 7.3 Hz, 3H).

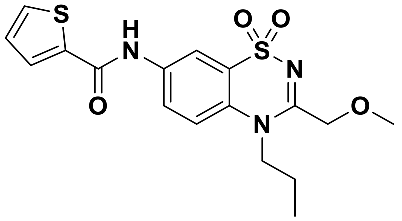

N-(3-(Methoxymethyl)-1,1-dioxido-4-propyl-4H-benzo[e][1,2,4]thiadiazin-7-yl)thiophene-2-carboxamide; NCGC00532347-01:

LC-MS t2 = 4.47 min, M+H = 394. 1H NMR (400 MHz, DMSO-d6) δ 10.62 (s, 1H), 8.33 (d, J = 2.5 Hz, 1H), 8.14 (dd, J = 9.4, 2.5 Hz, 1H), 8.06 (dd, J = 3.8, 1.2 Hz, 1H), 7.92 (dd, J = 4.9, 1.1 Hz, 1H), 7.77 (d, J = 9.4 Hz, 1H), 7.26 (dd, J = 5.0, 3.8 Hz, 1H), 4.50 (s, 2H), 4.12 (t, J = 7.6 Hz, 2H), 3.38 (s, 3H), 1.70 (h, J = 7.5 Hz, 2H), 0.91 (t, J = 7.3 Hz, 3H).

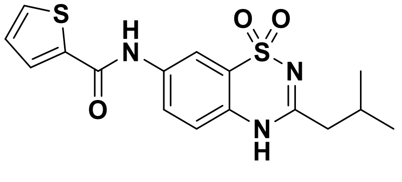

N-(3-Isobutyl-1,1-dioxido-4H-benzo[e][1,2,4]thiadiazin-7-yl)thiophene-2-carboxamide; NCGC00532401-01:

LC-MS t2 = 4.29 min, M+H = 364.

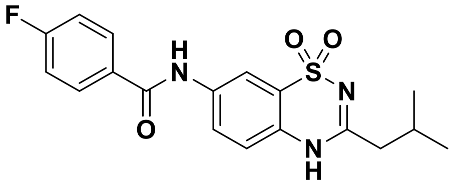

4-Fluoro-N-(3-isobutyl-1,1-dioxido-4H-benzo[e][1,2,4]thiadiazin-7-yl)benzamide; NCGC00532402-01:

LC-MS t2 = 4.51 min, M+H = 376.

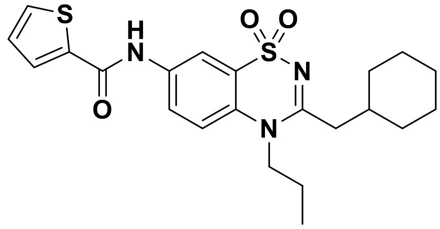

N-(3-(Cyclohexylmethyl)-1,1-dioxido-4-propyl-4H-benzo[e][1,2,4]thiadiazin-7-yl)thiophene-2-carboxamide; NCGC00532404-01:

LC-MS t2 = 5.81 min, M+H = 446. 1H NMR (400 MHz, DMSO-d6) δ 10.60 (s, 1H), 8.30 (d, J = 2.5 Hz, 1H), 8.11 (dd, J = 9.4, 2.5 Hz, 1H), 8.05 (dd, J = 3.9, 1.1 Hz, 1H), 7.91 (dd, J = 5.0, 1.1 Hz, 1H), 7.71 (d, J = 9.4 Hz, 1H), 7.25 (dd, J = 5.0, 3.8 Hz, 1H), 4.11 (t, J = 7.7 Hz, 2H), 2.66 (d, J = 6.6 Hz, 2H), 1.89 (ddd, J = 11.0, 7.4, 3.8 Hz, 1H), 1.75 (t, J = 11.6 Hz, 2H), 1.73 – 1.64 (m, 4H), 1.61 (s, 0H), 1.20 (dt, J = 27.1, 12.3 Hz, 3H), 1.11 – 0.98 (m, 2H), 0.94 (t, J = 7.3 Hz, 3H).

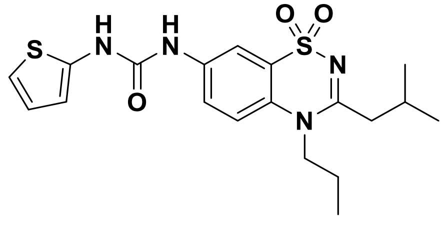

1-(3-Isobutyl-1,1-dioxido-4-propyl-4H-benzo[e][1,2,4]thiadiazin-7-yl)-3-(thiophen-2-yl)urea; NCGC00537523-01:

LC-MS t2 = 5.12 min, M+H = 421. 1H NMR (400 MHz, DMSO-d6) δ 9.82 (s, 1H), 9.22 (s, 1H), 8.11 (d, J = 2.6 Hz, 1H), 7.69 (dd, J = 9.3, 2.6 Hz, 1H), 7.61 (d, J = 9.4 Hz, 1H), 6.91 (dd, J = 5.5, 1.4 Hz, 1H), 6.87 – 6.78 (m, 1H), 6.61 (dd, J = 3.7, 1.5 Hz, 1H), 4.09 (t, J = 7.8 Hz, 2H), 2.66 (d, J = 7.3 Hz, 2H), 2.18 (p, J = 6.7 Hz, 1H), 1.68 (q, J = 7.6 Hz, 2H), 0.99 (d, J = 6.6 Hz, 6H), 0.93 (t, J = 7.3 Hz, 3H).

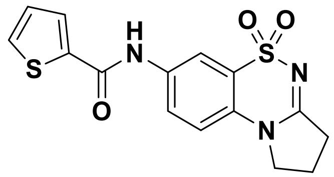

N-(5,5-Dioxido-2,3-dihydro-1H-benzo[e]pyrrolo[2,1-c][1,2,4]thiadiazin-7-yl)thiophene-2-carboxamide; NCGC00537660-01:

LC-MS t2 = 3.80 min, M+H = 348.

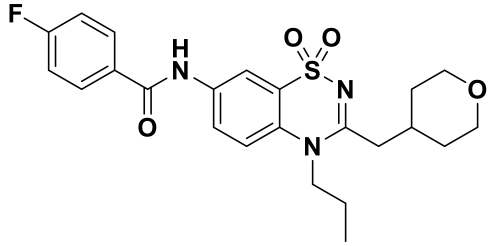

N-(1,1-Dioxido-4-propyl-3-((tetrahydro-2H-pyran-4-yl)methyl)-4H-benzo[e][1,2,4]thiadiazin-7-yl)-4-fluorobenzamide; NCGC00537661-01:

LC-MS t2 = 4.83 min, M+H = 460. 1H NMR (400 MHz, DMSO-d6) δ 10.64 (s, 1H), 8.39 (d, J = 2.5 Hz, 1H), 8.15 – 8.03 (m, 3H), 7.71 (d, J = 9.4 Hz, 1H), 7.40 (dd, J = 9.9, 7.9 Hz, 2H), 4.12 (t, J = 7.8 Hz, 2H), 3.84 (dd, J = 11.2, 4.0 Hz, 2H), 2.75 (d, J = 6.9 Hz, 2H), 2.21 – 2.09 (m, 1H), 1.69 (t, J = 10.4 Hz, 4H), 1.40 – 1.22 (m, 3H), 0.95 (t, J = 7.3 Hz, 3H).

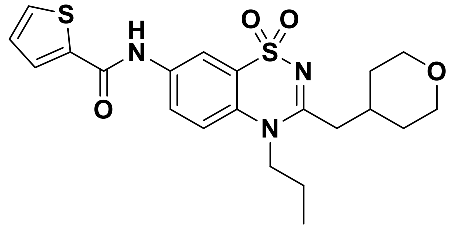

N-(1,1-Dioxido-4-propyl-3-((tetrahydro-2H-pyran-4-yl)methyl)-4H-benzo[e][1,2,4]thiadiazin-7-yl)thiophene-2-carboxamide; NCGC00537663-01:

LC-MS t2 = 4.66 min, M+H = 448. 1H NMR (400 MHz, DMSO-d6) δ 10.60 (s, 1H), 8.30 (d, J = 2.5 Hz, 1H), 8.11 (dd, J = 9.3, 2.6 Hz, 1H), 8.05 (dd, J = 3.8, 1.2 Hz, 1H), 7.94 – 7.88 (m, 1H), 7.71 (d, J = 9.4 Hz, 1H), 7.25 (dd, J = 5.0, 3.7 Hz, 1H), 4.12 (t, J = 7.7 Hz, 2H), 3.89 – 3.80 (m, 2H), 2.74 (d, J = 6.9 Hz, 2H), 2.14 (s, 2H), 1.69 (t, J = 10.8 Hz, 4H), 1.40 – 1.22 (m, 3H), 0.95 (t, J = 7.3 Hz, 3H).

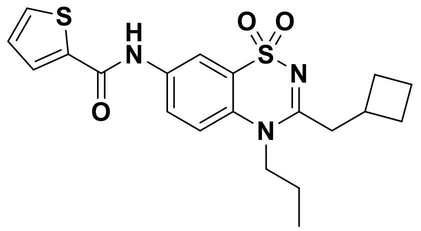

N-(3-(Cyclobutylmethyl)-1,1-dioxido-4-propyl-4H-benzo[e][1,2,4]thiadiazin-7-yl)thiophene-2-carboxamide; NCGC00537664-01:

LC-MS t2 = 5.28 min, M+H = 418. 1H NMR (400 MHz, DMSO-d6) δ 10.59 (s, 1H), 8.29 (d, J = 2.5 Hz, 1H), 8.14 – 8.07 (m, 1H), 8.05 (dd, J = 3.9, 1.2 Hz, 1H), 7.91 (dd, J = 4.9, 1.1 Hz, 1H), 7.69 (d, J = 9.4 Hz, 1H), 7.25 (dd, J = 5.0, 3.8 Hz, 1H), 4.08 (t, J = 7.7 Hz, 2H), 2.94 (d, J = 7.2 Hz, 2H), 2.76 (p, J = 7.6 Hz, 1H), 2.16 – 2.05 (m, 2H), 1.93 – 1.64 (m, 6H), 0.95 (t, J = 7.3 Hz, 3H).

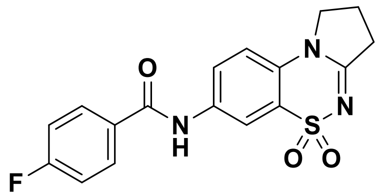

N-(5,5-Dioxido-2,3-dihydro-1H-benzo[e]pyrrolo[2,1-c][1,2,4]thiadiazin-7-yl)-4-fluorobenzamide; NCGC00537667-01:

LC-MS t2 = 4.07 min, M+H = 360. 1H NMR (400 MHz, DMSO-d6) δ 8.26 (s, 1H), 7.71 (d, J = 9.0 Hz, 1H), 7.43 (s, 1H), 7.18 – 7.11 (m, 1H), 3.82 (s, 2H), 2.63 (s, 2H), 2.18 (s, 2H).

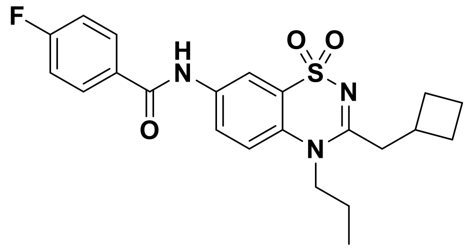

N-(3-(Cyclobutylmethyl)-1,1-dioxido-4-propyl-4H-benzo[e][1,2,4]thiadiazin-7-yl)-4-fluorobenzamide; NCGC00538288-01:

LC-MS t2 = 5.55 min, M+H = 430. 1H NMR (400 MHz, DMSO-d6) δ 10.62 (s, 1H), 8.38 (d, J = 2.5 Hz, 1H), 8.14 – 8.03 (m, 3H), 7.69 (d, J = 9.4 Hz, 1H), 7.40 (t, J = 8.9 Hz, 2H), 4.08 (t, J = 7.8 Hz, 2H), 2.95 (d, J = 7.2 Hz, 2H), 2.76 (p, J = 7.7 Hz, 1H), 2.11 (d, J = 7.1 Hz, 2H), 1.78 (ddq, J = 37.9, 22.2, 8.3, 7.4 Hz, 6H), 0.95 (t, J = 7.3 Hz, 3H).

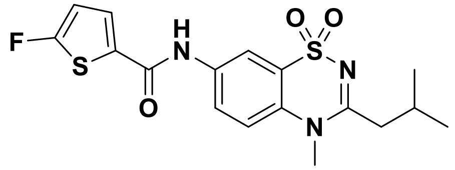

5-Fluoro-N-(3-isobutyl-4-methyl-1,1-dioxido-4H-benzo[e][1,2,4]thiadiazin-7-yl)thiophene-2-carboxamide; NCGC00651708-01:

LC-MS t2 = 4.87 min, M+H = 396.

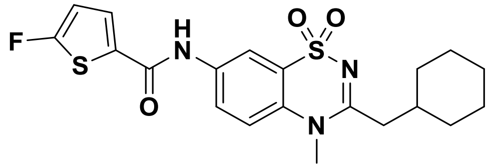

N-(3-(Cyclohexylmethyl)-4-methyl-1,1-dioxido-4H-benzo[e][1,2,4]thiadiazin-7-yl)-5-fluorothiophene-2-carboxamide; NCGC00651709-01:

LC-MS t2 = 5.46 min, M+H = 436.

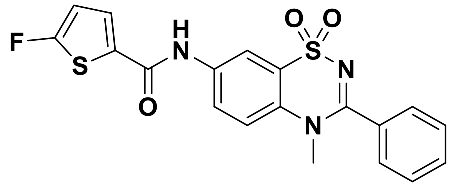

5-Fluoro-N-(4-methyl-1,1-dioxido-3-phenyl-4H-benzo[e][1,2,4]thiadiazin-7-yl)thiophene-2-carboxamide; NCGC00651712-01:

LC-MS t2 = 4.83 min, M+H = 416.

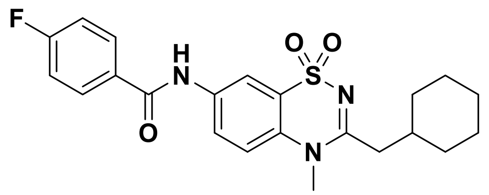

N-(3-(Cyclohexylmethyl)-4-methyl-1,1-dioxido-4H-benzo[e][1,2,4]thiadiazin-7-yl)-4-fluorobenzamide; NCGC00651716-01:

LC-MS t2 = 5.36 min, M+H = 430.

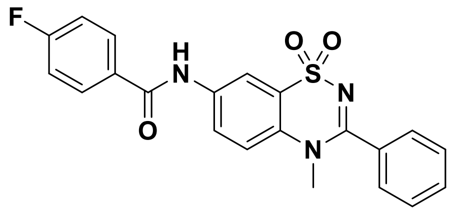

4-Fluoro-N-(4-methyl-1,1-dioxido-3-phenyl-4H-benzo[e][1,2,4]thiadiazin-7-yl)benzamide; NCGC00651719-01:

LC-MS t2 = 4.70 min, M+H = 410.

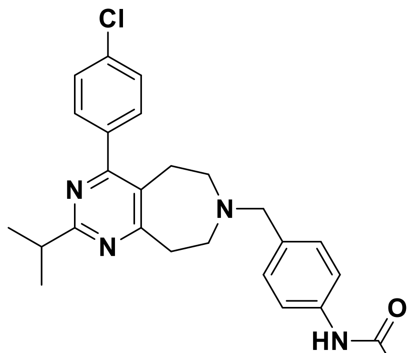

N-(4-((4-(4-Chlorophenyl)-2-isopropyl-5,6,8,9-tetrahydro-7H-pyrimido[4,5-d]azepin-7-yl)methyl)phenyl)acetamide; NCGC00374598:

LC-MS t2= 4.45 min, M+H=449.2. 1H NMR (400 MHz, DMSO-d6) δ 10.09 (s, 1H), 9.92 (s, 1H), 7.69 – 7.51 (m, 6H), 7.43 (d, J = 8.3 Hz, 2H), 4.31 (d, J = 4.7 Hz, 2H), 3.68 (s, 1H), 3.57 (s, 1H), 3.20 (s, 5H), 3.18 – 3.07 (m, 2H), 2.54 (s, 3H), 2.06 (s, 3H), 1.28 (d, J = 6.9 Hz, 6H).

References

1. Rai G *et al*. Pyrazole-based lactate dehydrogenase inhibitors with optimized cell activity and pharmacokinetic properties. *J Med Chem* **63**, 10984-11011 (2020).
2. Antonova-Koch Y *et al.* Open-source discovery of chemical leads for next-generation chemoprotective antimalarials. *Science* **362**, eaat9446 (2018).
